# *Berrembi Jarragboo‐Boorroo Wajawoorroo Men'Gawoom Gijam* (Gija Healthy Skin Story): Two‐Way Learning for Healthy Skin

**DOI:** 10.1002/hpja.70111

**Published:** 2025-10-02

**Authors:** Stephanie L. Enkel, Hannah M. M. Thomas, Rebecca Famlonga, Madeline Purdie, Cherylene Nocketta, Shirley Purdie, Eileen Bray, Tracy McRae, Abbey Ford, Tammy Gibbs, Cheryl Bridge, Asha C. Bowen

**Affiliations:** ^1^ Wesfarmers Centre of Vaccines and Infectious Diseases, The Kids Research Institute Australia (Formerly Telethon Kids Institute) University of Western Australia Nedlands Western Australia Australia; ^2^ Warmun Community Perth Wetsern Australia Australia; ^3^ Department of Infectious Diseases Perth Children's Hospital Nedlands Western Australia Australia

**Keywords:** health promotion, indigenous health, skin health, *Streptococcus pyogenes*, two‐way learning

## Abstract

**Issue Addressed:**

Remote‐living Aboriginal children in Australia contend with higher rates of skin infections than non‐Indigenous children. This work was embedded within a stepped‐wedge, cluster randomised controlled trial aiming to halve the rate of skin infections in remote Kimberley communities. It outlines and reflects upon the co‐development of a health promotion resource in partnership with the East Kimberley community of Warmun, whilst understanding community perceptions of its impact.

**Methods:**

Through a community participatory action research methodology over several years, relationship building and consultation identified there was a community preference for health promotion resources that documented both traditional and Western ways of supporting skin health. Two‐way learning was prioritised throughout the development process. Yarning methodology informed evaluation activities, with data analysed thematically.

**Results:**

The resulting resource *Berrembi Jarragboo‐boorroo Wajawoorroo Men'gawoom Gijam* (*Gija Healthy Skin Story*) was launched in March 2023. To date, over 500 hard copies of this and the subsequent Kriol version—*Dijan Wen Wi Tokin Bela Propa Good Wan Skin Gota Gija*—have been distributed. Eight Warmun community members and service providers participated in the evaluation yarns with themes specific to the development of the book, an assessment of impact and community‐identified strengths.

**Conclusion:**

*Berrembi Jarragboo‐boorroo Wajawoorroo Men'gawoom Gijam* (*Gija Healthy Skin Story*) exemplifies the elements of co‐design emphasized across the Kimberley and Australia; opportunities for two‐way learning, preference for community priorities and highlighting culture above all else.

**So What?:**

Health promotion activities completed within remote Aboriginal communities should prioritize two‐way understanding, authentic relationships, Aboriginal‐led, local language inclusion, equitable resourcing and ongoing evaluation to ensure that the results and outcomes are impactful for the communities involved.

## 1 Introduction

Aboriginal culture is underpinned by kinship and connection to Country, critical for holistic health and wellbeing [1]. However, remote‐living Aboriginal children in Australia continue to contend with high rates of skin infections, with up to 45% living with impetigo at any time [2]. Also known as skin sores, impetigo is a skin infection driven by *Staphylococcus aureus* and *Streptococcus pyogenes* [3] (Strep A), which, when left untreated, can progress to severe downstream diseases affecting the blood (sepsis) [4], bones and joints [5], kidneys (acute post‐streptococcal glomerulonephritis) [6] and heart (acute rheumatic fever and rheumatic heart disease) [7]. Skin sores can often also be a result of scabies, a pruritic skin infection caused by the mite *Sarcoptes scabiei* var. *hominis*, which transmits from person‐to‐person [8]. Experienced by up to 35% of remote‐living Aboriginal children at any time [9], scabies can be a precursor to impetigo, with scratching breaking the skin barrier and facilitating entry of bacteria [10]. The social determinants of health, namely poor housing and lack of healthy home hard‐ and soft‐ware [11], and the ongoing impacts of colonisation, all contribute to this high burden of skin infections in remote Aboriginal communities [12].

The See, Treat and Prevent (SToP) skin sores and scabies Trial aimed to reduce the burden of skin infections in Aboriginal children in the Kimberley, working across nine remote communities [13]. Whilst focusing on improving the diagnosis (See) and treatment (Treat) of skin infections through school‐based surveillance and clinical initiatives, this is the first trial to go beyond the biomedical model for skin health and incorporate activities aimed at preventing skin infections (Prevent). Prevention projects were incorporated into the study to empower community members to design, develop and implement skin health promotion messaging and resources in a place‐based, community‐led way [14]. Central to this approach was valuing Aboriginal cultural knowledge alongside biomedical strategies, recognising that health is sustained through both ways of working. As an example, many Aboriginal communities across Australia today retain the use of traditional medicinal practices—bush medicine—often aligning such practices alongside biomedicine for a holistic approach to prevention and treatment [15]. These practices have transcended millennia. Bush medicine knowledge is sacred to communities [16] and was shared with us as researchers across the lifetime of the Trial as a two‐way and holistic approach to keeping skin healthy.

The SToP Trial researchers prioritized a Community Participatory Action Research (CPAR) approach, viewing the community as equal partners in the research process [17, 18], with Indigenous research methodologies underpinning and informing practice. The format, design and outputs of any Prevention activities were not established until co‐researchers in each community became involved, consultations were completed and partnerships between all parties were firmly established. In this manner, these activities were aligned with the Five Qualities of Co‐design as outlined by the Kimberley Aboriginal Law and Cultural Centre (KALACC); two‐way understanding, authentic relationships, Aboriginal‐led, equitable resourcing and ongoing evaluation [19]. In this study, we aim to outline and reflect upon the development of a skin health promotion resource in partnership with an East Kimberley community and understand community perceptions of its impact.

## 2 Methods

### 2.1 Study Setting

**FIGURE 1:**
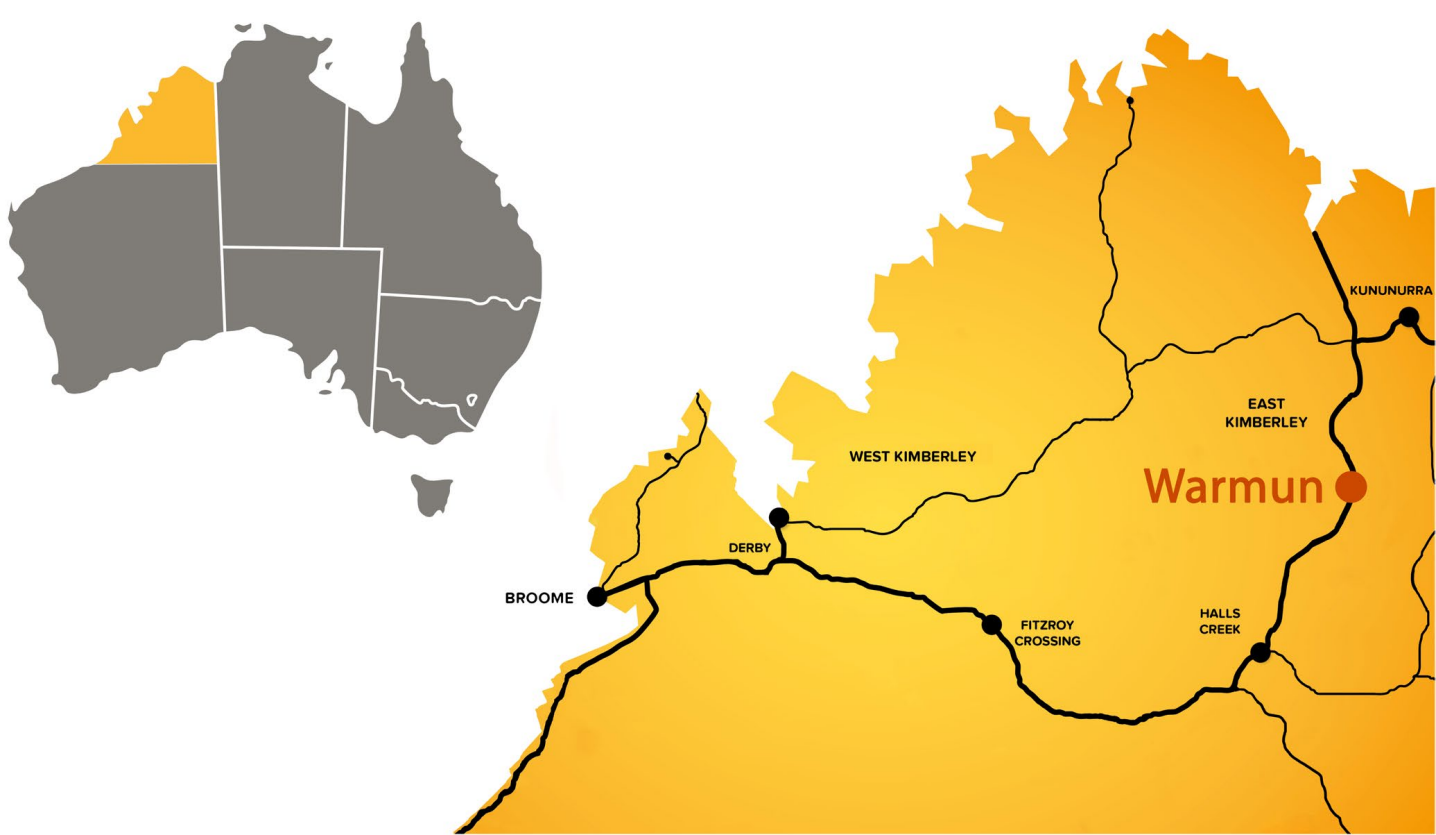
A map of Australia, with the Kimberley region highlighted in yellow. Warmun community is highlighted.

The community of Warmun (formerly known as Turkey Creek) is 160 kilometres (km) north of Halls Creek and 200 km south of Kununurra in the East Kimberley region of Western Australia (Figure 1). At the 2021 census, Warmun had a population of 457, with most identifying as either Aboriginal or Torres Strait Islander [20]. The traditional owners are the Gija people who have maintained strong links to culture and language despite the significant challenges posed by colonisation. During Warmun's involvement in the SToP Trial, Trial researchers visited the community 13 times between 2019 and 2022 [13]. During these visits, relationships were established with key community members, and many co‐design conversations were had about potential prevention activities to reduce the incidence of skin infections [14]. At the end of 2021, community members in Warmun decided that a two‐way skin health promotion book documenting both traditional and western approaches to supporting skin health would be beneficial.

### 2.2 Community Consultation

**FIGURE 2:**
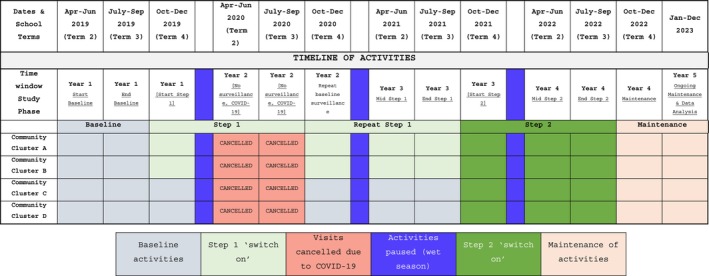
‘See’, ‘Treat’ and ‘Prevent’ activity delivery details and timeline.

Building relationships within participating SToP Trial communities was emphasised before and during the data collection phase of the study, underpinned by the CPAR approach [17]. From 2017, community visits occurred semi‐regularly as Trial establishment was underway, with a local Aboriginal Community Controlled Organization (ACCO)—Nirrumbuk Environmental Health and Services (NEHS)—familiar with the community, engaged to consent families prior to data collection commencing in 2019 [21]. As guided by the Trial framework [13], emphasis on building connections focused initially on the school and clinic as sites for surveillance and treatment activities. As Trial staff continued visiting Warmun, opportunities to engage with community members beyond these settings naturally evolved, and these connections were maintained during 2020 when visits were disrupted by COVID‐19 and associated community closures [22] (Figure 2).

In 2021 the SToP Trial team was able to begin visiting Warmun again. A community barbecue was hosted by a community‐based co‐researcher, the research team and NEHS to discuss the project with residents. Through yarns with attending children and Elders over a meal and many cups of tea about the work of the SToP Trial, perspectives on skin infections in Warmun and suggestions on how best to spread Healthy Skin messaging were explored. Yarning is a recognised Indigenous qualitative research method that privileges storytelling, relationships and reciprocity as a way of generating knowledge in a culturally safe context [23]. As the Warmun community was in the process of launching a Gija Dictionary [24], this yarning involved a significant exchange of language with a focus on healthy skin words. Throughout discussions, it was clear that community members preferred a resource that documented both traditional and Western ways of supporting skin health. Collectively, researchers and community members decided to co‐develop a storybook with an emphasis on bush medicine. This decision built on more than 2 years of sustained engagement between the Trial team and Warmun community members, during which a community co‐researcher and several Elders had been actively involved in ongoing discussions about prevention activities.

The Community Chairperson at the time approached the SToP Trial, expressing interest in being involved to help facilitate the inclusion of traditional knowledge, whilst guiding the Trial team through future discussions with Elders. A school health teacher was also engaged, being both interested and well‐placed to engage children in the project.

A visit to Warmun was planned for early 2022 with three key aims: to further plan the development of the storybook; to aid community co‐researchers with gathering community consents for the resource; and to facilitate healthy skin workshops at the school to involve children in the storybook illustrations. However, this visit was cancelled due to a community outbreak of COVID‐19 [22]. The SToP Trial team delivered the planned school‐based element virtually by attending a health lesson via Microsoft Teams. Children identified different types of skin infections, discussed the steps they would take if they had a skin infection, and developed illustrations on how they keep their skin healthy for inclusion in the storybook. In August 2022, members of the SToP Trial team were invited to attend the local annual Women's Two‐way Healing camp, held on sacred Gija Women's Country, to continue work on the storybook.

### 2.3 Co‐Development of the Book

Discussions during the Women's Camp further developed the initial concept of the storybook, including which information should be contained and what imagery would be best. A key component of these discussions was the knowledge translation of bush medicines into the book. In accordance with the two‐way theme, elders and women shared stories of traditional bush medicines for skin health and the SToP Trial team shared information about skin infection diagnosis, treatment and prevention. The Community Chairperson also invited a photographer and videographer to attend the camp to document events and take photos and video that could later be used in the healthy skin storybook and supporting materials. During the camp, women painted ochre artworks for use in the storybook and recommended that resources include Gija, Kriol and English language, as all are used in the Warmun community.

The Community Chairperson was asked how she and other community members would like to be involved and opted to provide feedback on a first draft produced by the Trial team. Between August and October 2022, SToP Trial staff worked on a draft resource clinically aligned with the first edition of the National Healthy Skin Guidelines [25] and incorporating all elements requested by community members during the Women's Camp. This proved to be an iterative process over an extended period. In October 2022, the first draft was printed and presented at an Elders meeting for feedback to ensure community voices were heard and incorporated early. Another community barbeque supported by NEHS was held with approximately 25 adults and 50 children in attendance and community members were encouraged to write directly on the draft pages of the book to inform improvements. The draft resource was also taken to Elders, the clinic, school and early‐learning centre to capture further feedback, with a local Aboriginal Health Worker trainee providing significant input to the draft resource. To ensure that health promotion is founded on accurate clinical details, the resource was reviewed clinically. All edits were reviewed and incorporated where possible to reflect community voice.

### 2.4 Evaluation

During development and following the launch of the resource, community members were approached to take part in yarning activities to evaluate the storybook using convenience sampling methods. All yarns occurred at a time and place convenient and comfortable for the participants and were completed by SToP Trial team members familiar to the interviewee. Questions pertained to the process of development, thoughts on the final resource and opportunities for improvement. All participants provided informed, written consent to take part in yarns and have them recorded. Recordings were transcribed verbatim and analysed using Reflexive Thematic Analysis [26], a flexible coding process in conjunction with NVivo (QSR International) software analysis. This analysis informed the major themes that created the narrative of the development process of this health promotion resource and the perceptions of its impact within the Warmun community.

### 2.5 Ethics Statement

Ethical approval was granted by the health ethics review committees at the Child and Adolescent Health Service (RGS0000000584) and the WA Aboriginal Health Ethics Committee (Ref819). Approval was also provided by the University of WA (RA/4/20/4123), Catholic Education WA (RP2017/57) and WA Department of Education (D18/0281633). Reciprocal support was provided from the University of Notre Dame (Reference: 2021‐128F) and Murdoch University (2022/196).

## 3 Results

*Berrembi Jarragboo‐boorroo Wajawoorroo Men'gawoom Gijam (Gija Healthy Skin Story)* book (Appendix 1) [27] was launched in Warmun in March 2023 with a curry dinner (as requested by Elders) at the council building. Over 75 people attended the launch in the community. The Kriol version of the book—*Dijan Wen Wi Tokin Bela Propa Good Wan Skin Gota Gija* (Appendix 2) [28]—was completed later in 2023. Following community member edits, a graphic designer was involved to arrange the images, concepts and layout with iterative clinical review to ensure scientific validity and shared with the community when Trial results were delivered in August 2023.

To date, 300 books have been printed and provided to the Warmun community, with over 200 additional books distributed via 14 stakeholders working in the region. Eight Warmun community members and service providers participated in the yarning sessions, with all having knowledge of the development process of the *Berrembi Jarragboo‐boorroo Wajawoorroo Men'gawoom Gijam* (*Gija Healthy Skin Story*) and having exposure to the resource following its launch. Three primary themes were identified: those specific to the development of the book, an assessment of impact and community‐identified strengths.

### 3.1 Development of the Book

#### 3.1.1 The Process Was Positive and Created Pride

Most participants communicated that the book provoked feelings of pride in either themselves or in other community or family members.

Well, your resource; your journey, that you've followed with the Gija people, to make the book, has been incredible. And I think congratulations to all of you, because I'm really proud of the book, and you must all be so proud of the book! (Participant 5)

And I came to the dinner, the launch and it was really fantastic to see all the smiling faces and people so proud of the book. (Participant 6)

Participants were asked their thoughts on the community‐driven development process. The responses were overwhelmingly positive, with most responding with positive feedback and general satisfaction.

Researcher: And have you heard from anyone, anything that we could've done better in the process, like we're really keen to learn from the experience and grow, so if you've heard anything that, you know, we could've built on or done better, we'd love to know.

Participant: I haven't heard anything like that, no, no. All positive news.

(Participant 6)

As quoted by one participant who had been involved in the production of the book from inception;

I wasn't expecting it to look this flash…It's really good. You got words but you got a lot of pictures too, so people can thing, read it. But yeah, it's easy to understand I reckon. Plus we got our language in there. So people, the schools can learn them kids more about the language as well. (Participant 1)

#### 3.1.2 Suggested Improvements to the Process and Future Work

Half of the participants suggested minor improvements for the book. This included a male participant making several references to involving more men in the process next time. Other suggestions for improvements included linking the language coding to established colour codes already used in the school, making a video or digital resource with audio recordings of Gija language being spoken or sung and including more photos of bush medicine plants.

And so that then they're learning a little song, and we can video it, and they can have it on their iPads, so that they can watch it, and it has a bit of fun, and learning about skin–healthy skin, and what to do if you have sores, and what to look for. (Participant 5)

### 3.2 Impact

#### 3.2.1 Understanding Skin Health and Infections

Most participants viewed *Berrembi Jarragboo‐boorroo Wajawoorroo Men'gawoom Gijam* (*Gija Healthy Skin Story*) as a significant resource for teaching families the importance of skin health and indicated that the visuals within the book were useful in helping community members recognize skin sores and scabies and prompt people to seek medical attention from the clinic with confidence. Two participants also mentioned that the book helps to make the link between healthy skin and the prevention of more serious downstream health complications such as RHD. The book was also identified as an important educational tool for teaching about skin health within the school and the clinic.

Yeah, I think they'll learn, and they'll be more aware. There's is a sore there, sometimes you think oh is that bad enough to go see that clinic, because they don't wanna, at least they know it's getting worse. They'll look at the picture and they'll go down to the clinic. (Participant 2)

#### 3.2.2 Understanding Traditional Bush Medicine Use

All participants referenced the book contributing to the community's understanding of the use of traditional bush medicines in caring for skin to prevent skin disease and treat skin infections. Traditional bush medicine knowledge that the community is willing to share has been captured within the book and will remain a valuable resource for current and future generations.

So when we're talking to them out bush when we're doing all our bush medicine thing and when we're talking to them about which plant or tree to get for the bush medicine, our kids will know. Our future generation. (Participant 1)

#### 3.2.3 The Book as a Resource to Teach the Whole Community About Skin Health and Bush Medicine

Several participants viewed the book as a resource that the whole community, including outside service providers and clinic staff who work in the community, can use to learn about the importance of skin health and the use of bush medicine to maintain and treat skin infections.

I've worked in communities for over eight years, and this is the first book I have seen that is to do with health, and it's involved the Community, the children, service providers and I think it's a good way to go, and in the future there should be more of it. (Participant 8)

Some participants highlighted that the book provides valuable health promotion messages about washing people and linen and keeping the community clean in general.

And even for washing all the linen and stuff and hanging it out in the sun to kill(s) all the germs. We tell our young people that. Say you've got to wash it, good wash and then hang it out in the sun long time, like six or seven hours. It will kill all the bugs. (Participant 1)

#### 3.2.4 Strengthening Gija Language Use and Literacy

Over half of participants stated that the book would also encourage the use of Gija language, including the use of language names for bush medicine plants. Participants focused on the importance of this, particularly for young people, and additionally, the potential to improve general literacy via the book's two‐way presentation of Standard Australian English and Gija.

Participant: …Just say we having dinner with the kids, and you say that, in Gija, then the kid might ask you ‘what did you say,’ then you'll say it in English, you know?

Researcher: So it could be good for building language skills as well as skin and health.

Participant: Yeah. Yeah, and then you can come back again, ‘I'll say it again!’ you know? And you'll say the actual word, yes, you know? In Gija. (Participant 3)

#### 3.2.5 Intergenerational Skin Health Knowledge Transfer

Participants described the book as being a tool that could prompt intergenerational knowledge transfer between family members. Examples of this knowledge transfer included grandparents and parents passing knowledge onto children and grandchildren, and young people sharing this story with Elders.

And, yeah, the Gija side of all that, yeah it's very good, that way if we read it to our old people, they'll, yeah, ‘Oh, yeah, that's good,’ you know. And then we teach our kids, like teach them the thing about scabies and what they need to do, in, yeah, in our language. (Participant 8)

#### 3.2.6 The Audience for Berrembi Jarragboo‐Boorroo Wajawoorroo Men'gawoom Gijam (Gija Healthy Skin Story)

Participants listed family homes, the school, the clinic, the Council, the Women's and Art Centres and the Men's Shed as being places they had either seen the book or thought it would be a valuable resource to be placed within.

Having this book I think, like we have medical things everywhere, like even having this in our men's shed, having it in our art centre, [organization name], you know, even just talk about skin as well, like we can talk about it everywhere, not just the school and the women camp, you know what I mean? (Participant 3)

### 3.3 Community Identified Strengths of the Book

During the evaluation of the process, the community members also identified many strengths of the book. Notably, the use of Gija language was described by most participants as being an asset of the story and encouraging the use of the language within the community. In 2023, the Warmun community launched a Gija Dictionary [24] which has been seen as a way to sustain the Gija language at risk of being lost in the community. Participants spoke highly about the resource to re‐engage children—frequently speaking Kriol—with Gija.

Plus we got our language in there. So people, the schools can learn them kids more about the language as well. (Participant 1)

Many positive comments were made by participants about the visual aids used within the story. The photos of bush medicine, community members—including children—and the artwork were all referred to as making the book easier to read and more attractive. The photos of skin infections were also deemed useful in providing community members with confidence in identifying skin problems and then subsequently seeking treatment.

People they know helps, but any visuals really good, doesn't matter who it is. They see the picture, it will make them more, I reckon, the parents, ah I should go to the clinic, this sore is looking like this. They might'nt think ah ee right this sore. Make them more confident to go down to the clinic and get it checked. (Participant 2)

The use of simple language in addition to the pictures helped with understanding the content.

It's really good. You got words but you got a lot of pictures too, so people can thing, read it…But yeah, it's easy to understand I reckon. (Participant 1)

## 4 Discussion

The most frequently used word to describe *Berrembi Jarragboo‐boorroo Wajawoorroo Men'gawoom Gijam* (*Gija Healthy Skin Story*) was ‘deadly’. In Aboriginal English, the term deadly means ‘good’ or ‘fantastic’ and is used as praise [29]. Findings from this study reflected participants' views of this resource being ‘deadly’ with overwhelmingly positive feedback and strong reiteration of its strengths and the impacts it will have in the community. Several suggestions for minor improvements or future work were also received. The process of development reflected the two‐way learning and strengths‐based approach taken by all participants to share their respective knowledges about healthy skin, with respect and reciprocity.

In the context of communities facing competing health priorities, it is common for skin infections and sores to be perceived as ‘benign’ and thus become normalised [30]. SToP Trial communities highlighted this during the lifetime of the Trial, with frequent reference to the lack of awareness of the potential downstream consequences of skin sores [31, 32]. *Berrembi Jarragboo‐boorroo Wajawoorroo Men'gawoom Gijam* (*Gija Healthy Skin Story*) was noted by yarning participants to be a way of raising awareness of the importance of acting quickly should a skin sore arise—specifically among children—whilst retaining relevance to the community. From a clinical perspective, the baseline messaging of the resource will not change should alternative therapeutic guidelines become apparent; observe hygiene at home, do not share health software (i.e., towels) and visit the clinic if you have a sore.

Whilst *Berrembi Jarragboo‐boorroo Wajawoorroo Men'gawoom Gijam* (*Gija Healthy Skin Story*) retains specificity to a very small part of Australia, the journey embarked upon by SToP Trial researchers and the Warmun community reflects an example of best‐practice health promotion in an Aboriginal context [18]. This project was resourced mostly by time; considerations of the utmost importance but too frequently overlooked in the academic world by which this work was funded. Fortunately for the SToP Trial, the extended timeline of the project due to COVID‐19 disruptions [22] allowed for these elements; a method endorsed by The Kids Research Institute Australia Guidelines for the Standards for the Conduct of Aboriginal Health Research [33] and more often acknowledged in Aboriginal health research today. Relationship building, trust, shared experiences and time are all strongly interrelated in Aboriginal worldviews that demonstrate resilience and connection. These foundational principles must continue to be built into health promotion activities designed to impact the genuine issues maintaining gaps in life expectancy for Aboriginal people in Australia. The Four Priorities for the Coalition of Peaks [34] are also reflected here.

Throughout this process it was an honour for SToP Trial team members to have the knowledge of bush medicine shared with them and painted in local ochre by the rightful traditional owners during the Women's Camp. This example of place‐based translation emphasised the CPAR approach the team prioritised. Participants reflected on the importance of their community, their families and images of both being beautifully presented in the book—examples of the importance of place‐based and culturally relevant resources.

*Berrembi Jarragboo‐boorroo Wajawoorroo Men'gawoom Gijam* (*Gija Healthy Skin Story*), along with other resources co‐designed with Kimberley communities during the lifetime of the SToP Trial [35] has been highlighted as a practical example of excellence in health promotion in a new chapter included in the updated National Healthy Skin Guidelines [10]. This resource—intended for clinical use—emphasises the need for an appreciation of cultural context alongside biomedicine, noting that this will vary from community to community. As emphasised in the Guidelines, visual aids and cultural translation of resources can facilitate greater and more meaningful conversations between clinicians and individuals, empowering individuals and communities to be actively involved in their own health outcomes [10].

A limitation noted by yarning participants was the lack of involvement by Warmun men, which could have an influence on the reach of *Berrembi Jarragboo‐boorroo Wajawoorroo Men'gawoom Gijam* (*Gija Healthy Skin Story*). It is possible that the demographic of the visiting SToP Trial team members (all female) was a constraint in accessing more community members and is an important consideration in the context of Aboriginal health research, equity, inclusivity and health promotion. Despite best intentions to work face‐to‐face with school children, the team was kept from visiting by COVID‐19 outbreaks and resulting community closures; thus, the voices of children are not captured as comprehensively within the resource as first envisioned.

*Berrembi Jarragboo‐boorroo Wajawoorroo Men'gawoom Gijam* (*Gija Healthy Skin Story*) exemplifies the elements of co‐design emphasised across the Kimberley and Australia; opportunities for two‐way learning, preferencing community priorities and highlighting culture above all else. As articulated by the participants of this project, it is anticipated that *Berrembi Jarragboo‐boorroo Wajawoorroo Men'gawoom Gijam* (*Gija Healthy Skin Story*) will continue to be used in Warmun as a strategy to support healthy skin among children and families in the years to come.

## Conflicts of Interest

The authors declare no conflicts of interest.

## Supporting information


**Appendix 1:** Berrembi Jarragboo‐boorroo Wajawoorroo Men'gawoom Gijam (Gija Healthy Skin Story).

**Appendix 2:** Dijan Wen Wi Tokin Bela Propa Good Wan Skin Gota Gija (Gija Healthy Skin Story).

## Data Availability

Research data are not shared.
