# Supplementary material for: Berrembi Jarragboo‐Boorroo Wajawoorroo Men'Gawoom Gijam (Gija Healthy Skin Story): Two‐Way Learning for Healthy Skin
Source: Health Promot J Austr. 2025 Oct 2;36(4):e70111. doi: 10.1002/hpja.70111 (PMC12491125; doi:10.1002/hpja.70111)

Berrembi jarragboo-boorroo wajawoorroo men'gawoom Gijam

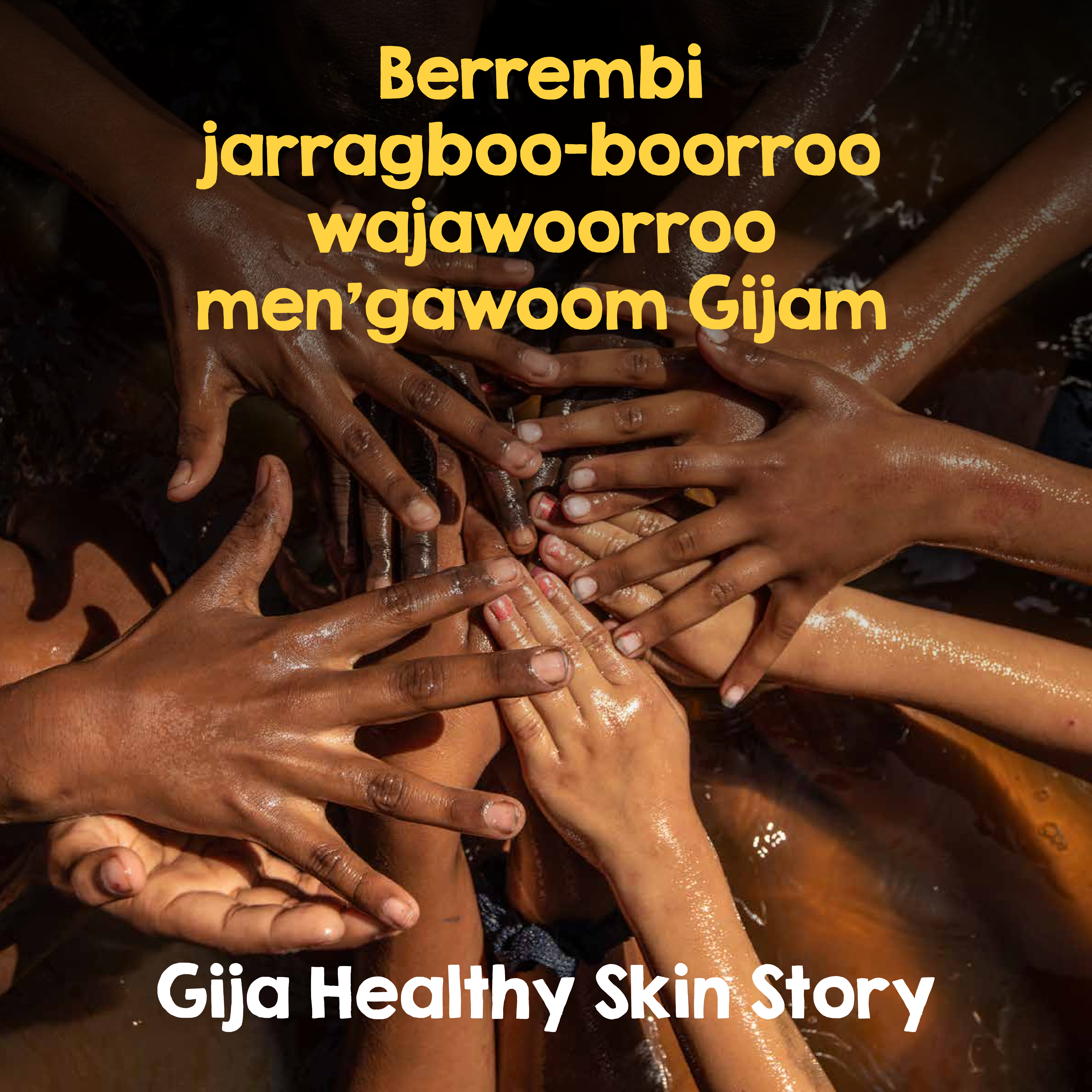

Gija Healthy Skin Story

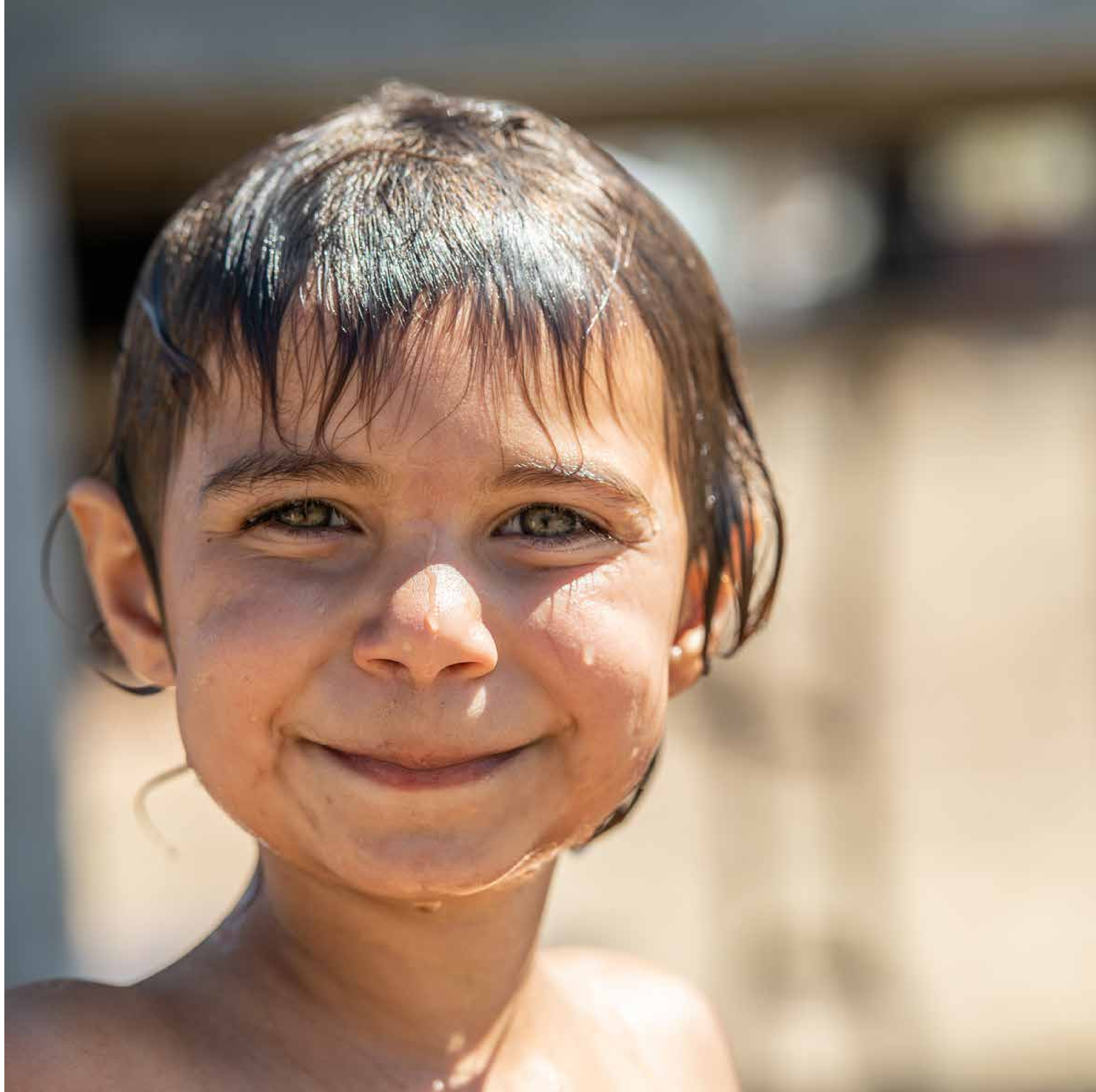

The Gija people are the Traditional Owners of the land and waterways on which this resource was produced. They have a strong, continuous connection to Country and Culture, especially traditional healing practices, and have shared their knowledge and wisdom in this resource so it may be celebrated for generations to come.

The following people were involved in making this resource:

Madeline Purdie and Cherylene Nocketta, who supported the project in Warmun community.

Mary Thomas, Mabel Juli, Shirley Purdie and Eileen Bray, who shared the traditional knowledge included in the resource.

Carol Johnson and Lauren Stone, who supported the project at Ngalangangpum Catholic school.

Madeline Purdie, Karen Nungatcha, Carol Juli, Sylvia Thomas, Lorraine Daylight, Jane Yalunga and Rochelle Peris, who provided the artwork.

Eileen Bray and Frances Kofod, who provided the translation.

Rose Malgil and Sonia Bray, who provided the content for the story.

The children of Warmun community, who provided the drawings.

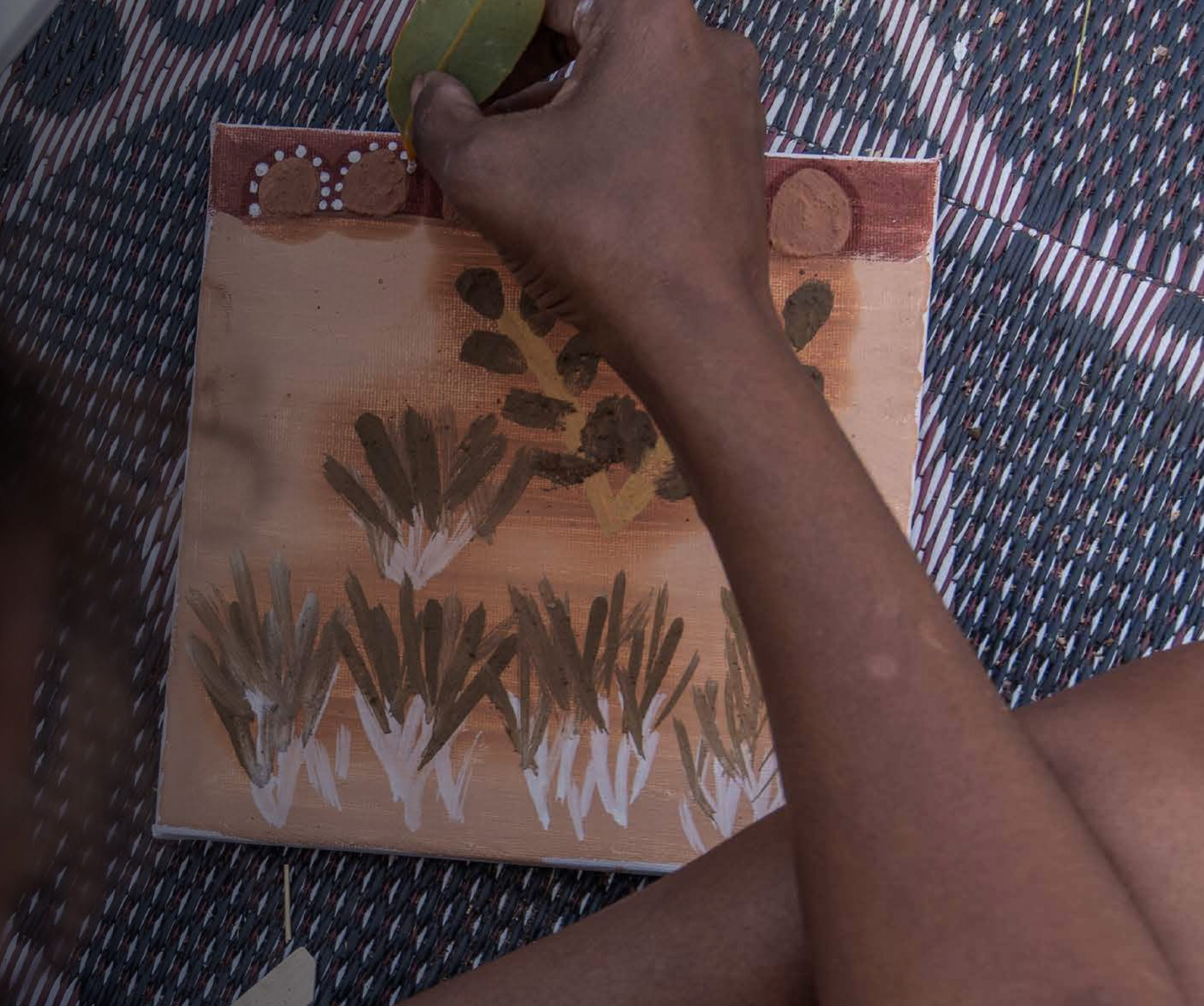

Our skin protects our bodies.

If our skin is sick, our bodies, kidneys and hearts can get sick.

Strong skin means strong bodies, strong kidneys and strong hearts.

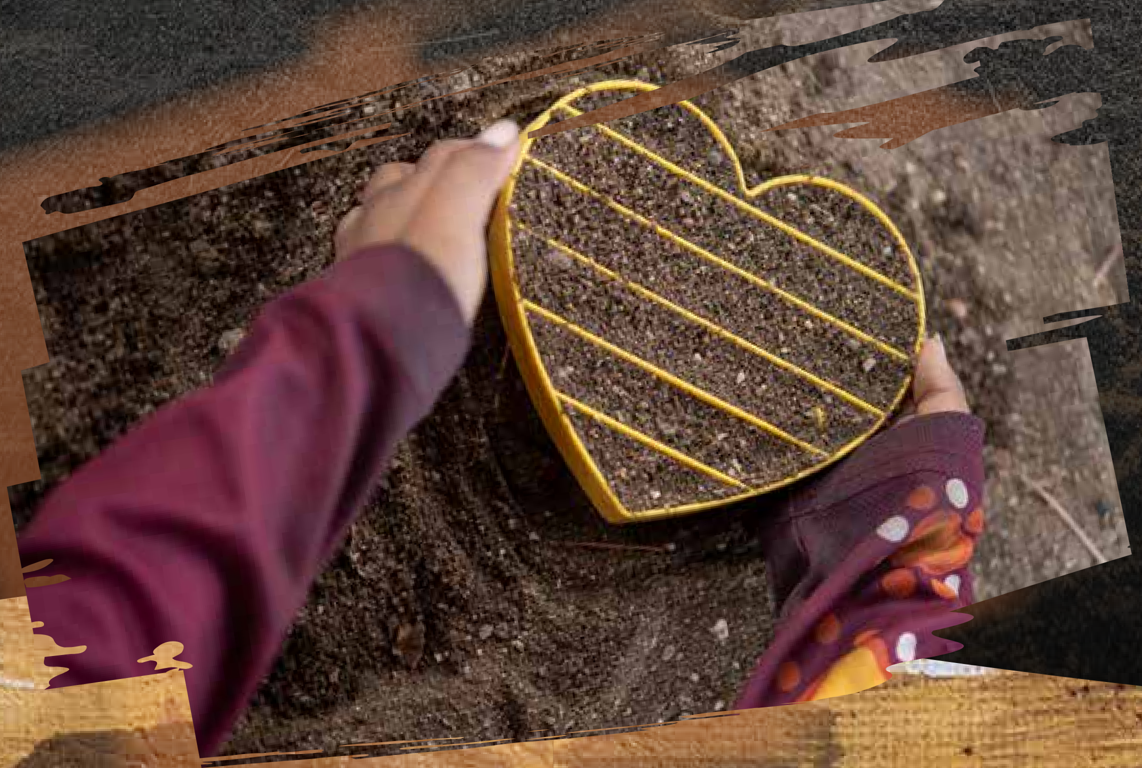

Wajawoorroo berrem yoowoorriyangem maroorr yimberriyinbe-yoowoo.

Wajawoorroo berrem, yilgoowoorroo-ngarri yimberriyinbe-yarri, warrernbeg yimberremenbe-yoowoo garajen, giningin doo werlmerren.

Dam yoowoorriyangem wajawoorroo bagarrjiya-ngarri boorroonboo, men'gawiyawa dambi garajbe-yoowoo doo giningim, doo werlmerrem.

There are lots of different skin sicknesses.

Skin sores, or school sores, are caused by germs called bacteria.

If they're not treated, they can lead to bad diseases like Rheumatic Heart Disease and kidney disease.

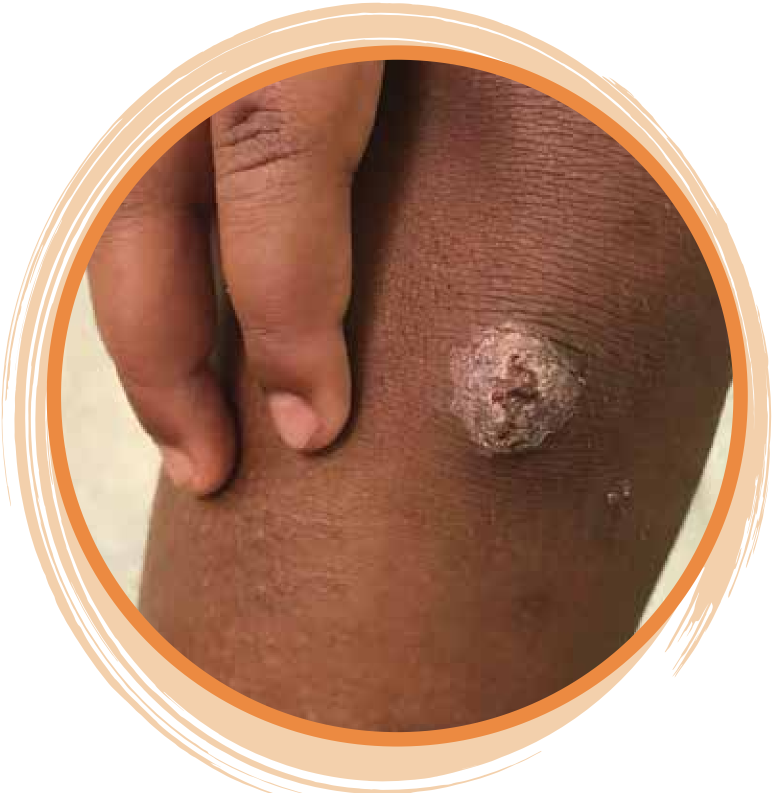

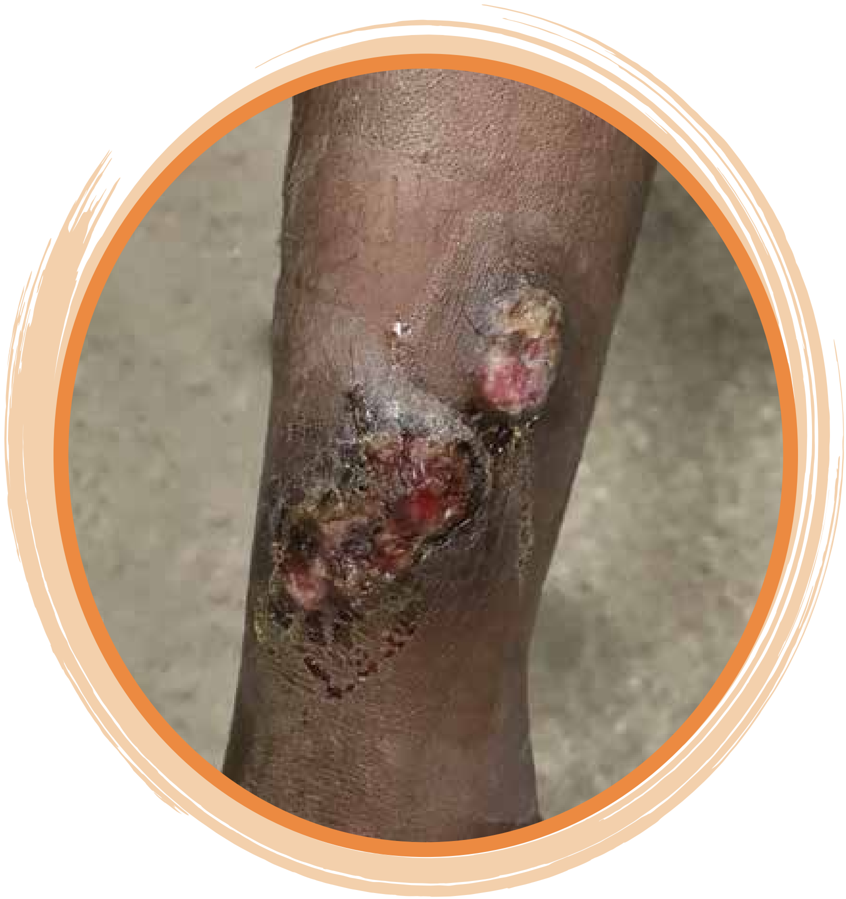

Melagawoom warrernbe booorroo-yoowoo wajawoorroo.

Bemberremenbe-ngarri gajim dam, thoowoorranyji wanyagem waj boorroorn-birri.

Ngoowan bimbirrimbe-ngarri gajim-boorroo waranggan, yilgoowoorroog bimbirrimbe giningin doo werlmerren.

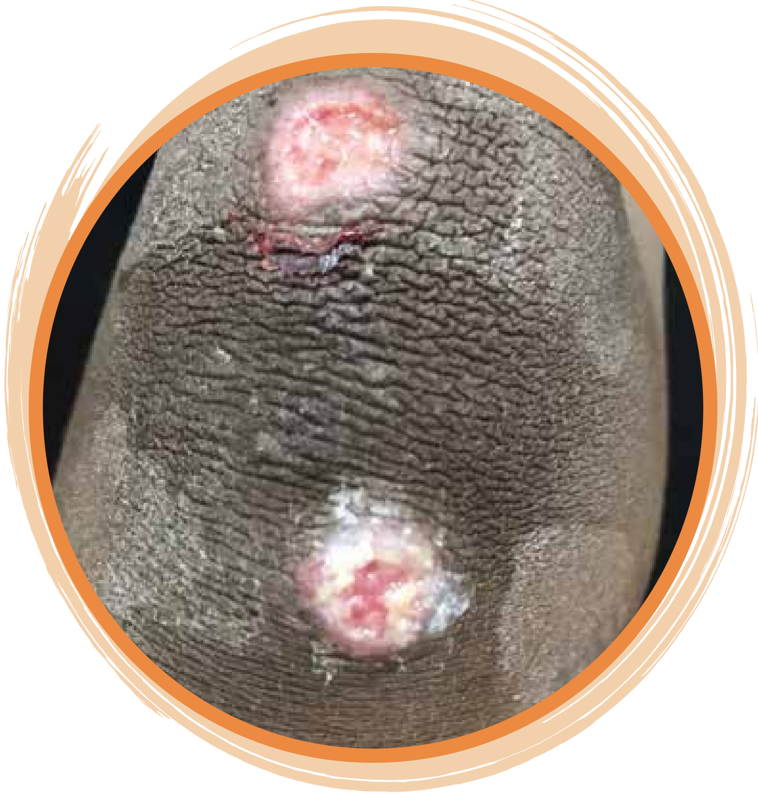

Scabies are cheeky bugs that camp in the skin. They are really itchy and too much scratching can lead to skin sores.

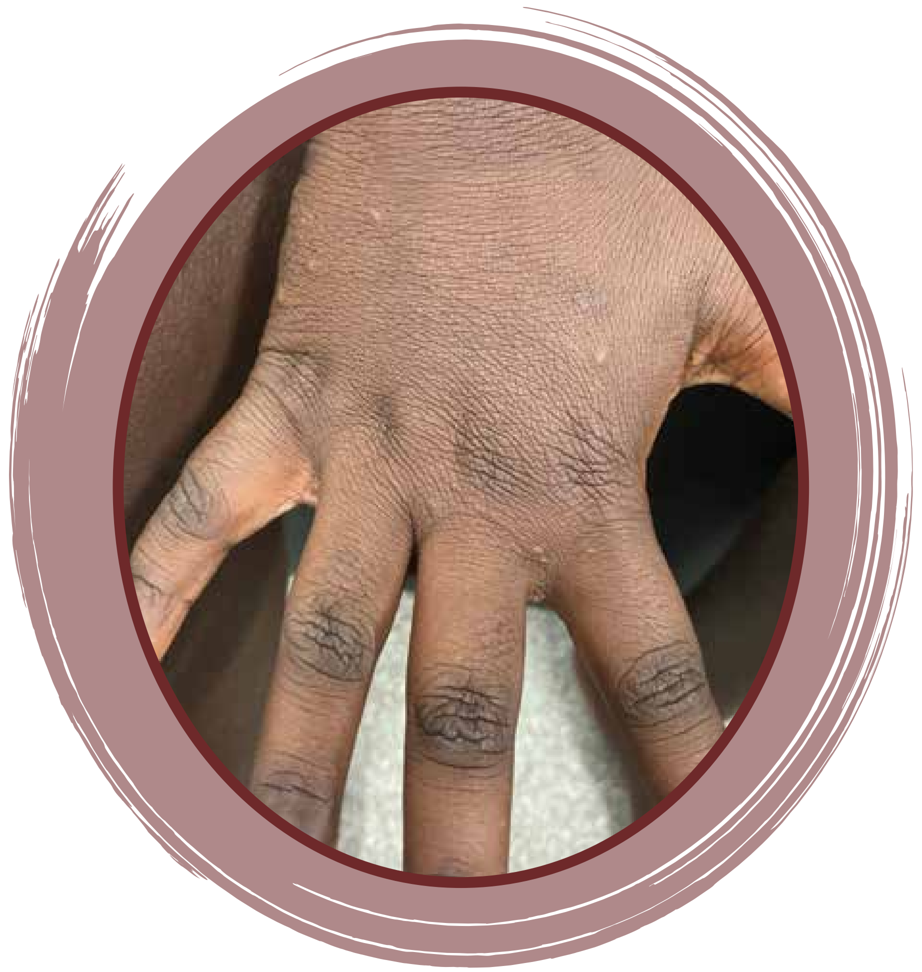

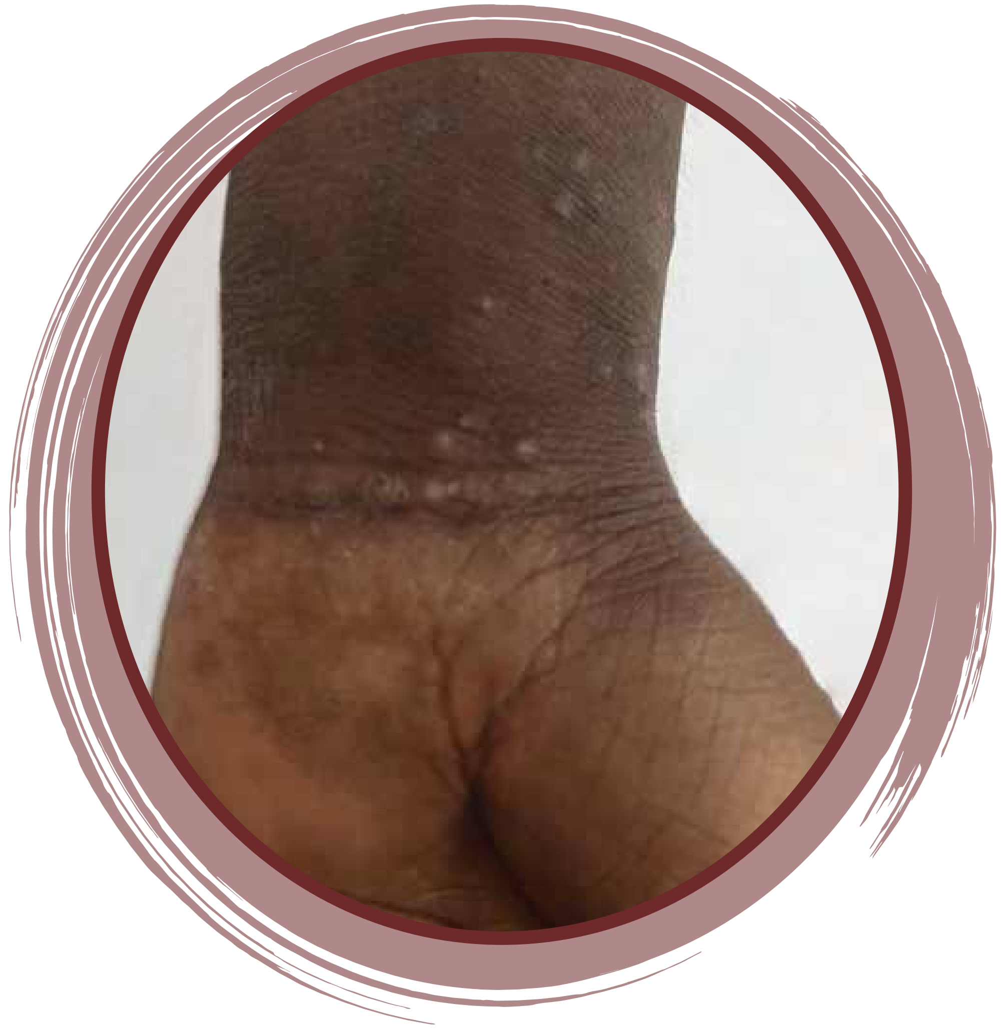

Dany wariwoony gajig-ngarri benemenji, nginjende-birri wajawoorroo. Dambi nyinggijam, girr-girr-ngarri berremiyanbe, gajig birriyiliyanbe.

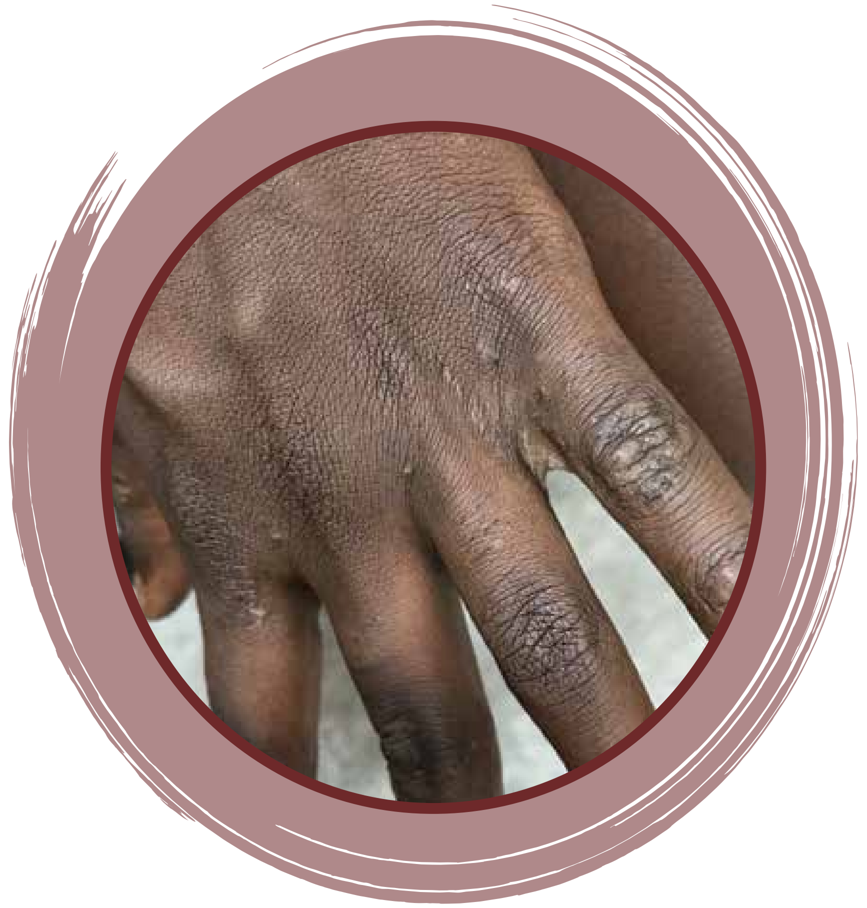

It's important to keep our skin strong.

For a long time our people have kept their skin strong with bush medicines.

Men'gawoom-boorroo wajawoorroo-boorroo-yoowoo maroorr-ngarri yambirrimnya.

Warna-warnarram maroorr yarremiyinya yoowoorriyangem-birri gibingarnam.

Bilirnji
(river gum)

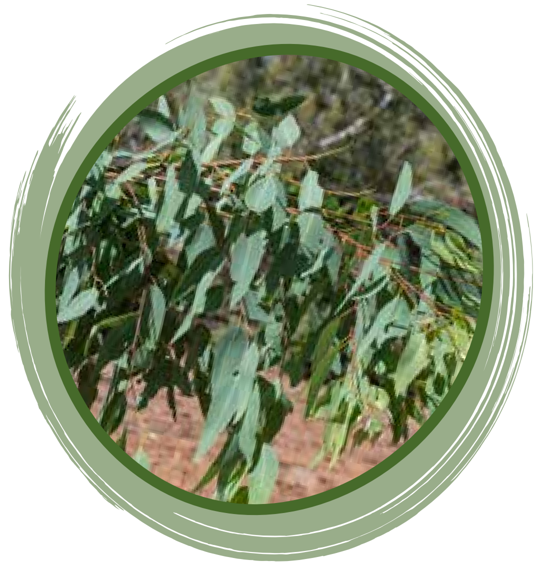

Thalngarrji
(snappy gum)

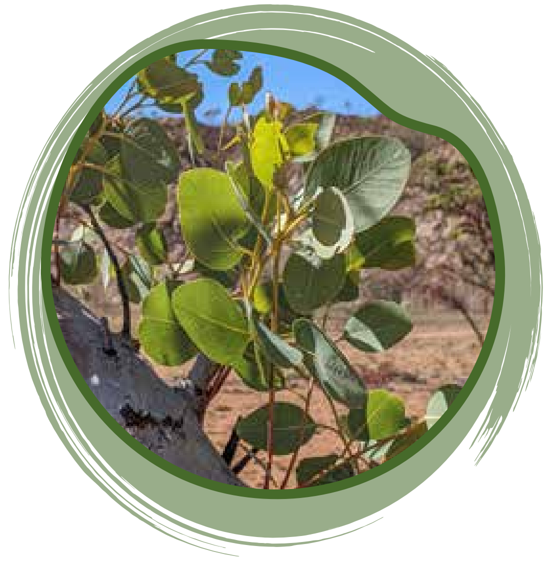

Ngarrngarrji
(lemongrass)

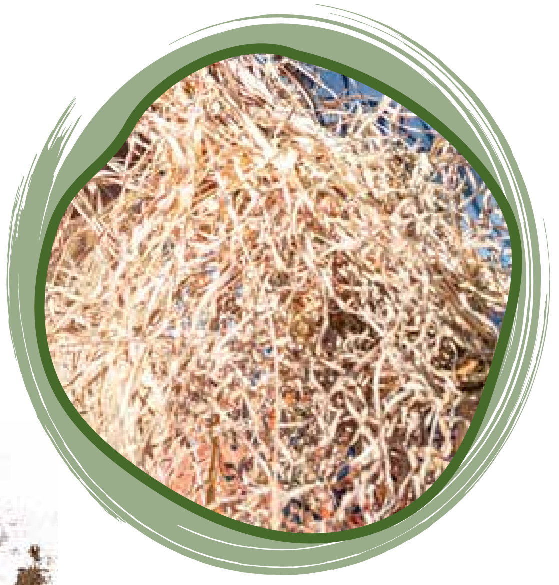

Miloowoony
(spinifex grass)

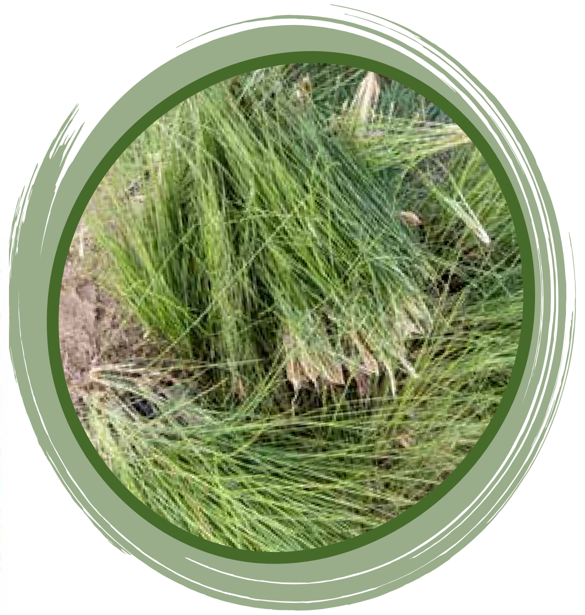

Lawoony
(lemonwood)

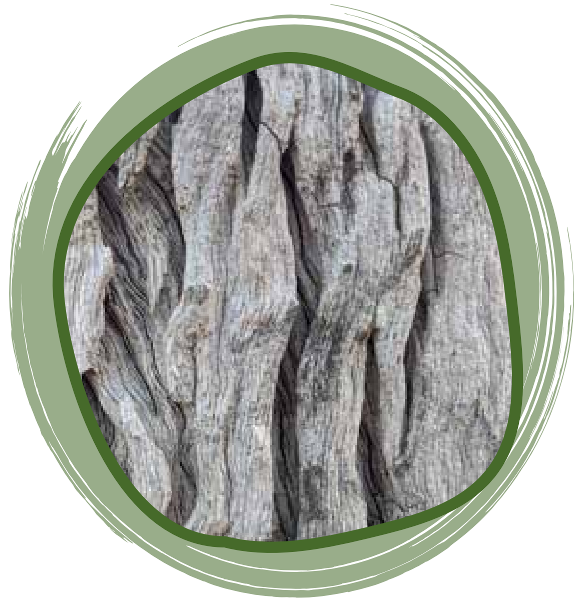

We can use these bush medicines to make a cream.

For the cream, first we boil up the leaves, spinifex and bark with oil and beeswax for as long as possible.

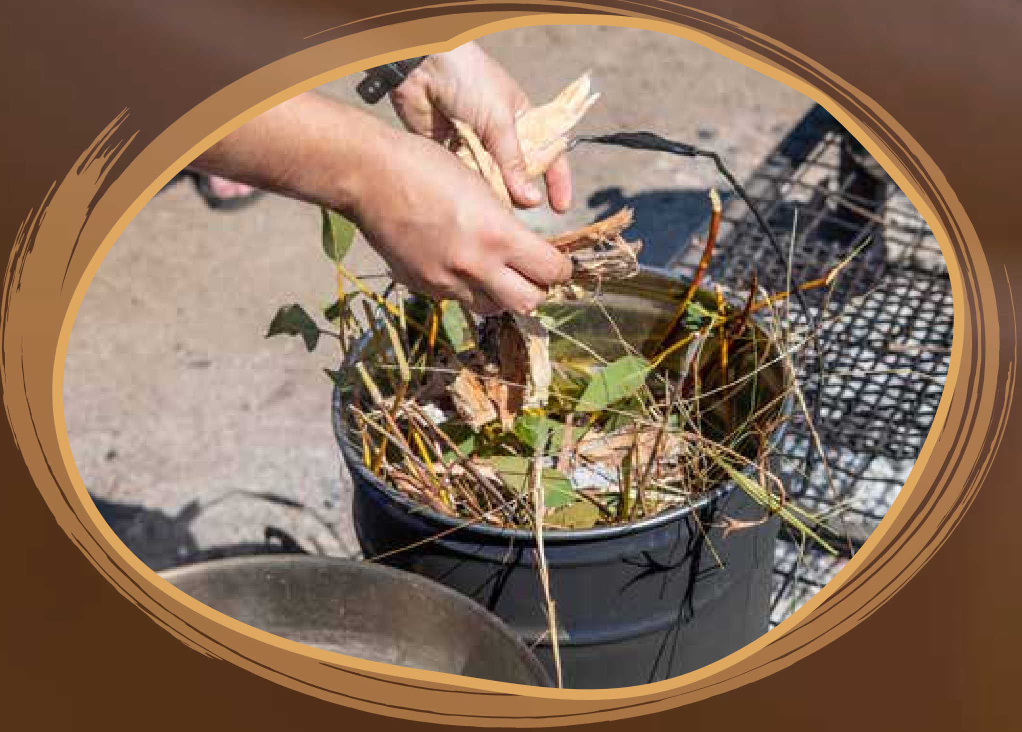

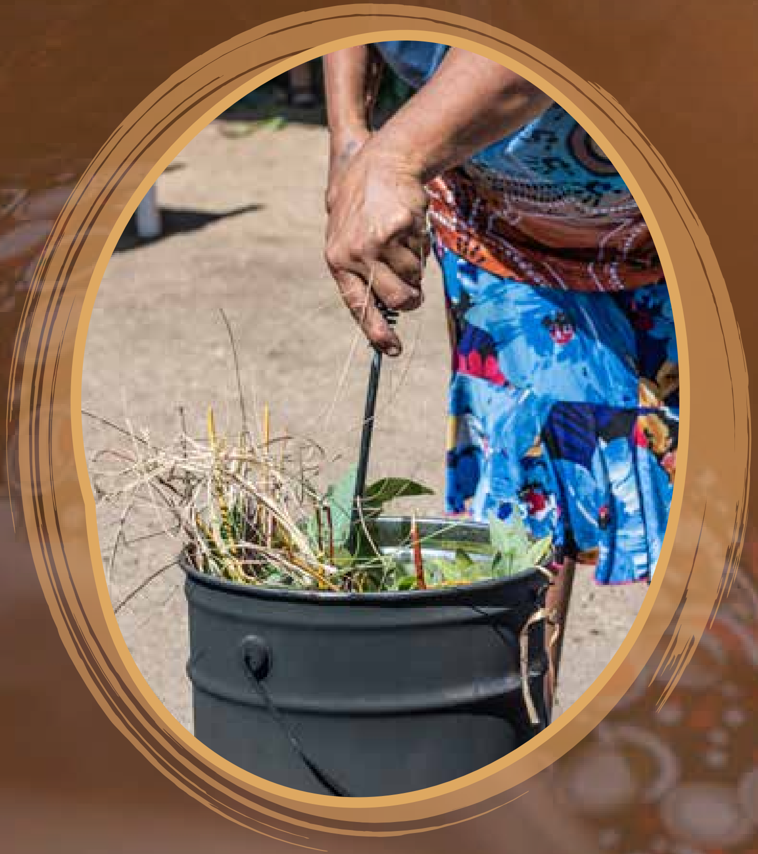

Berrembi yoowoorriyangem gibingarnam, ngararag yarroorn nyoon-nyoon-girrem men’gawoog-girrem.

Dambi gibingarnam ngarag-ngarri yarroorn, jaa yamberriyilinya ganarram, doo miliwoony, doo bernngam mooloonggoom-birri doo yagengem ganggarrjam dernag-girrem.

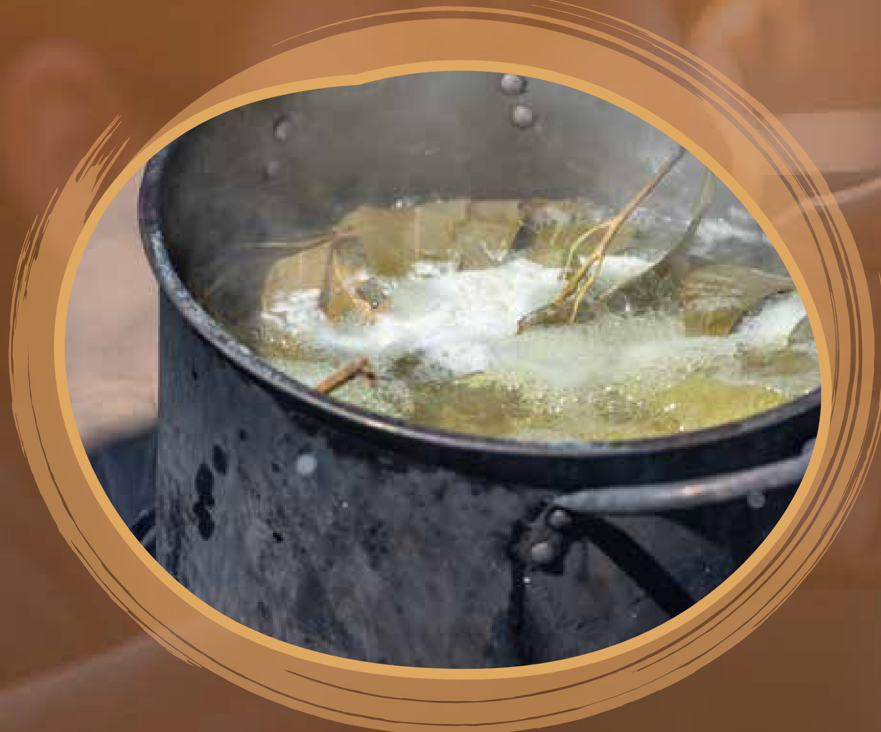

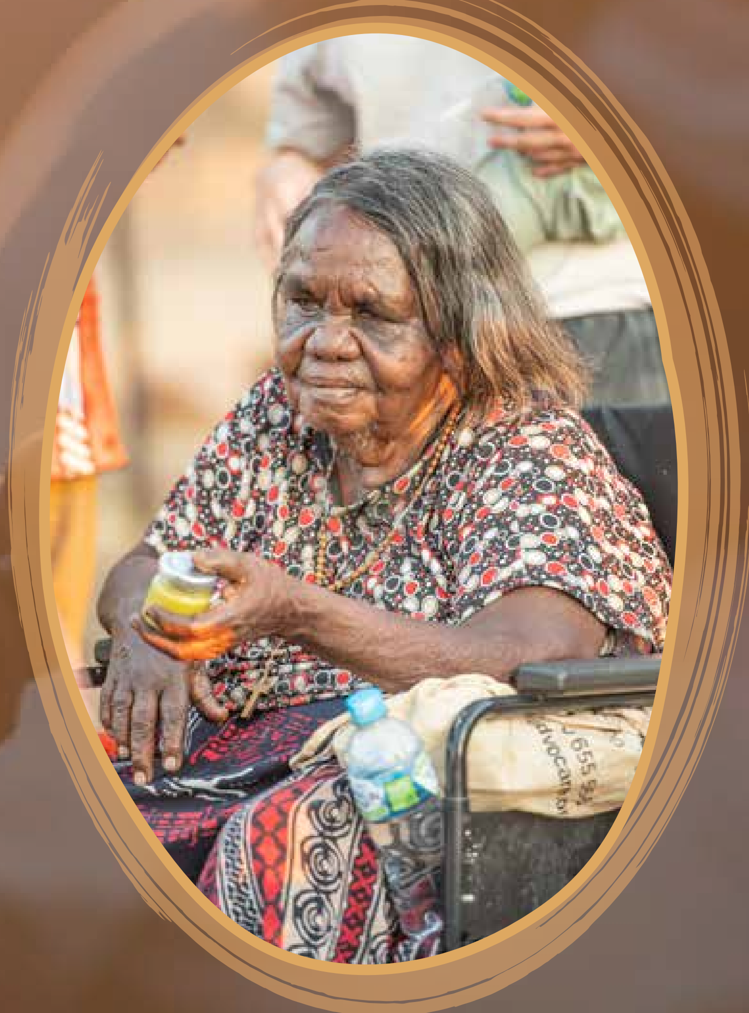

Second we pour this mixture through a cloth into small jars and let them cool.

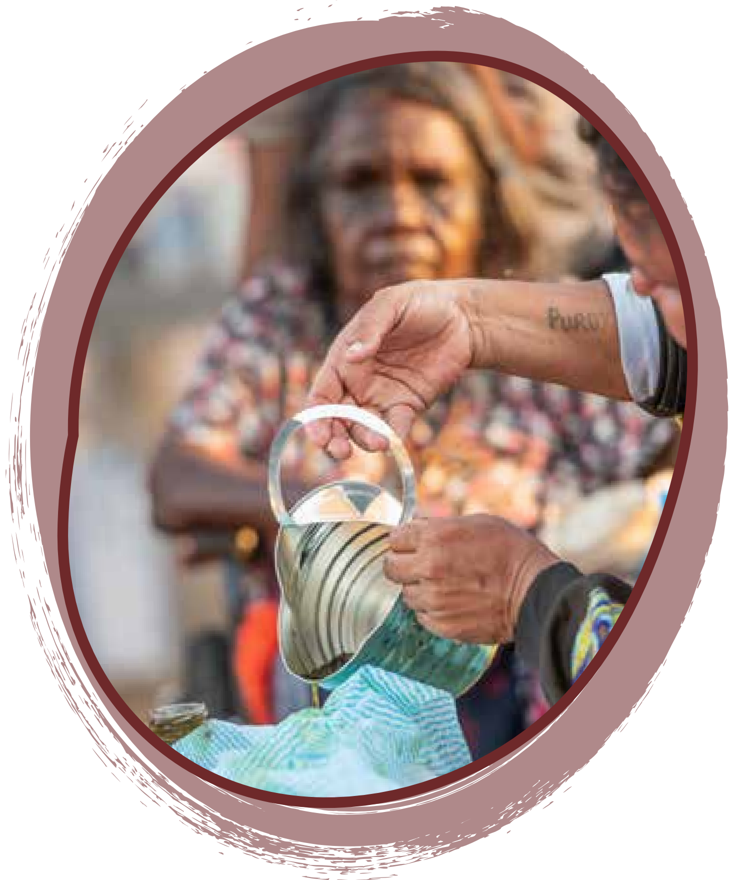

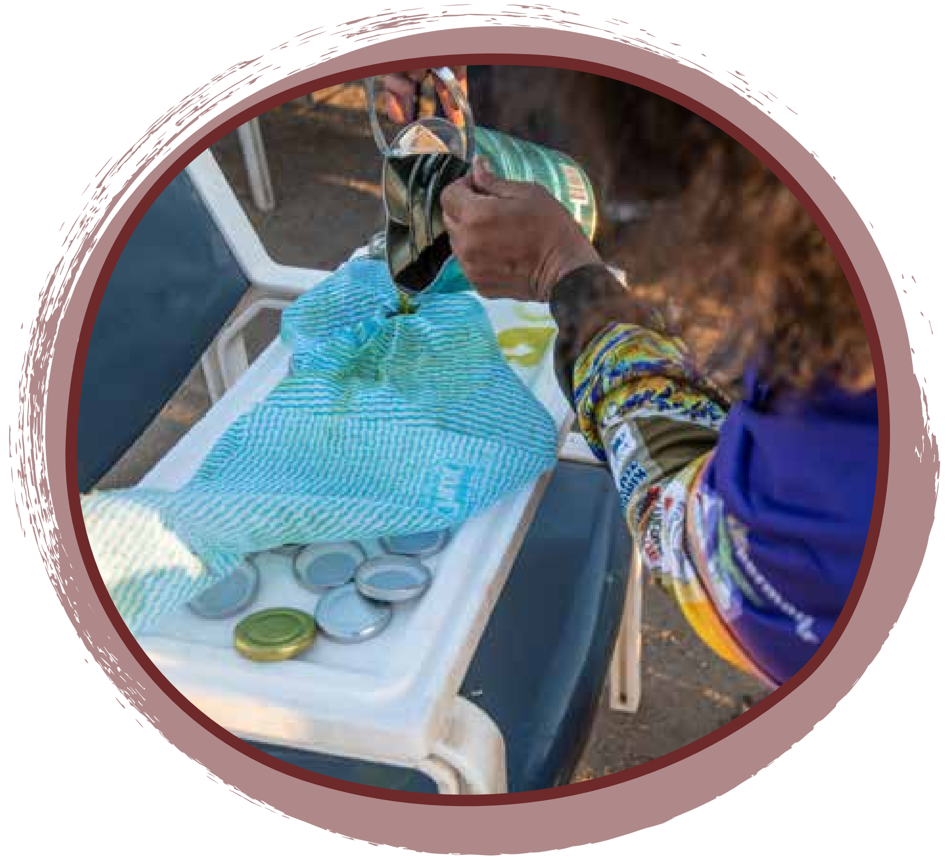

Yamberriyilinya berrem wanyagin jimbirlan, gawilig-girrem.

We rub these on our skin to keep it strong.

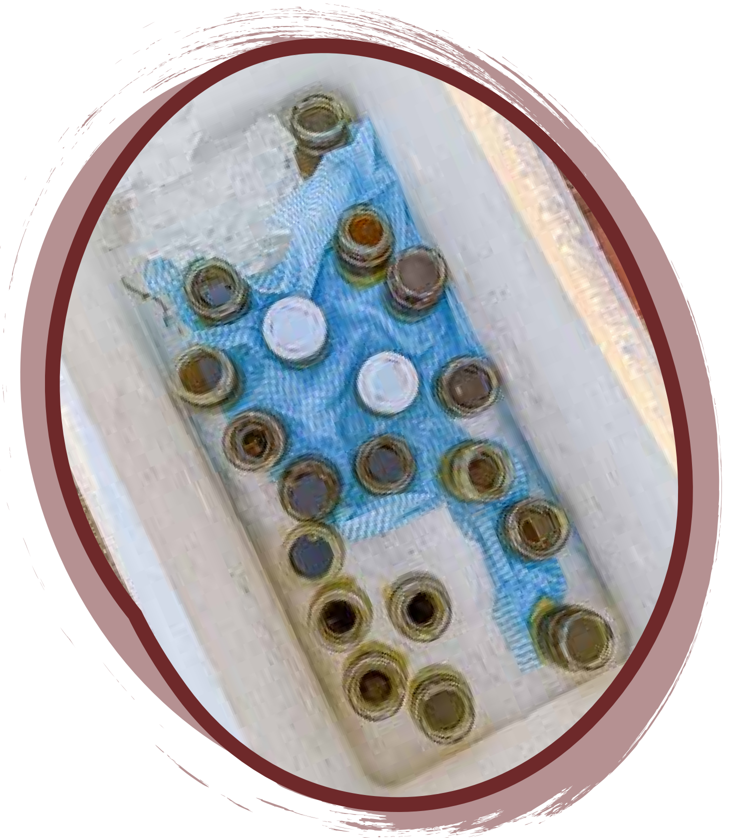

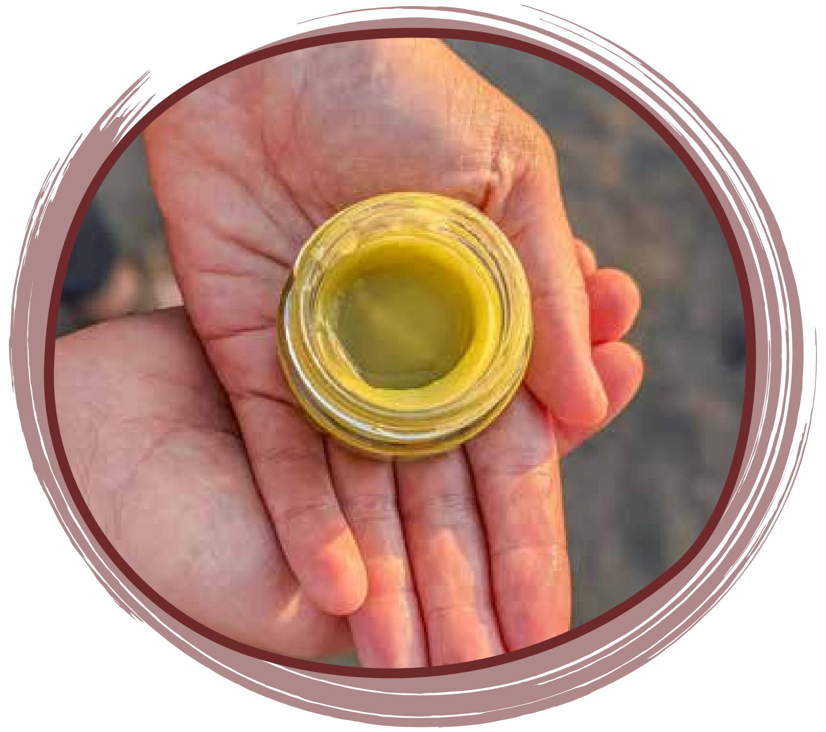

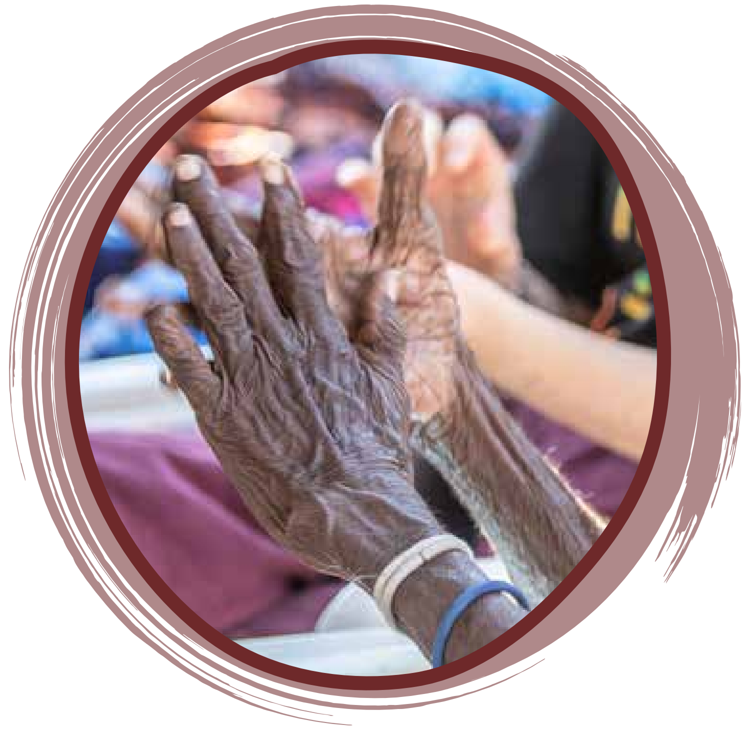

Nyoon-nyoon yarremiyanya wajawoorroo bagarrjig-girrem.

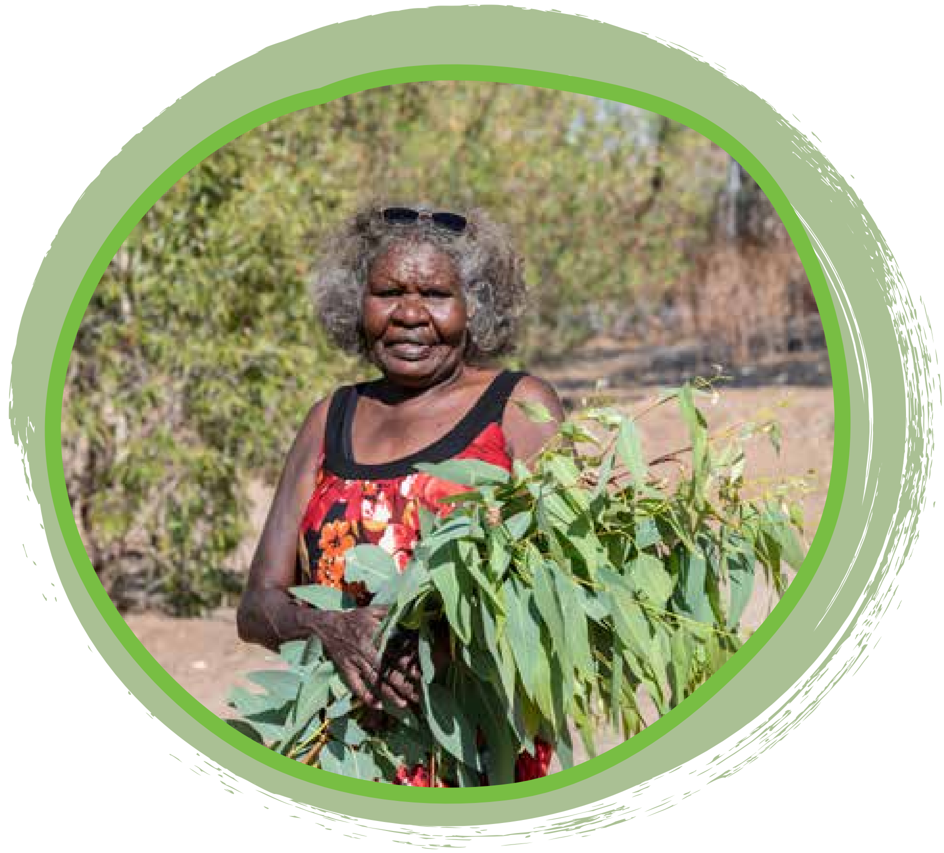

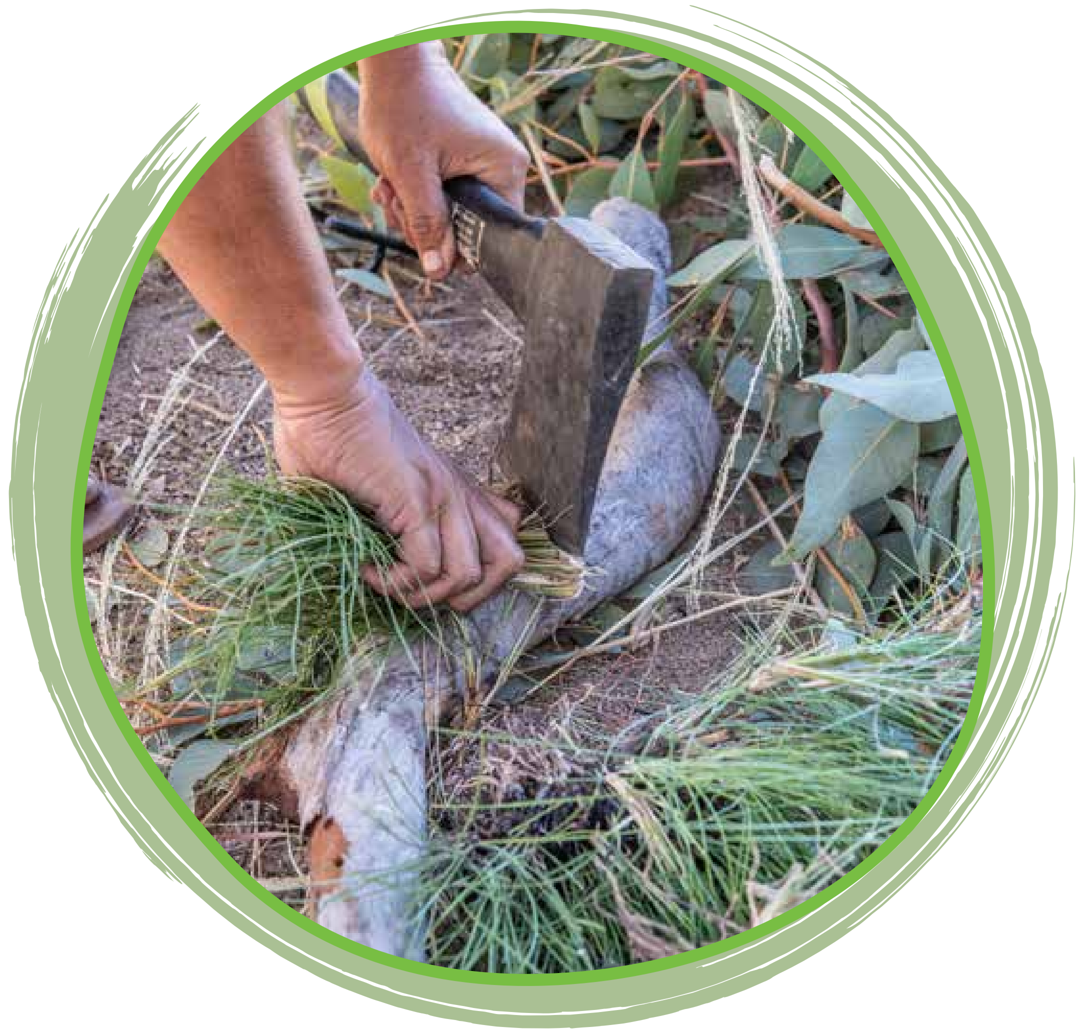

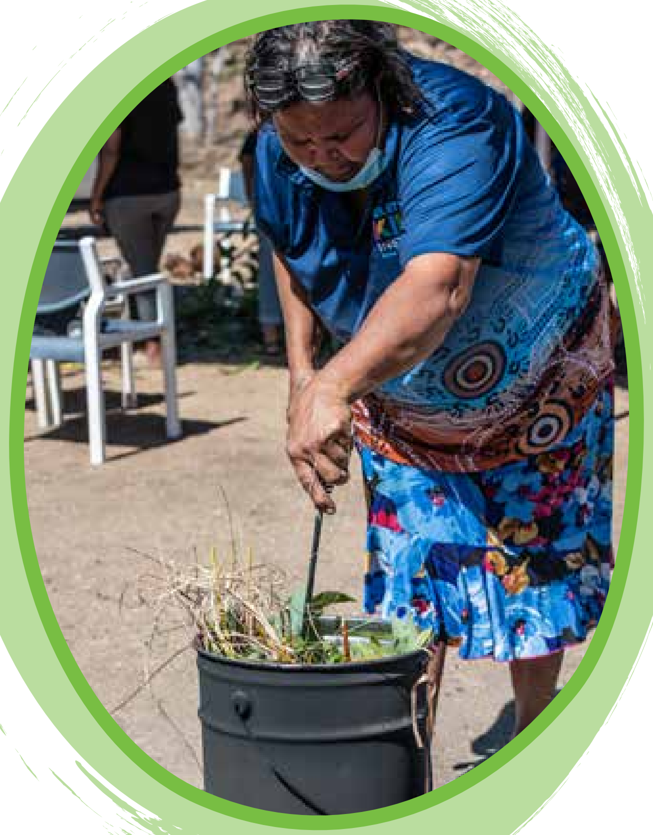

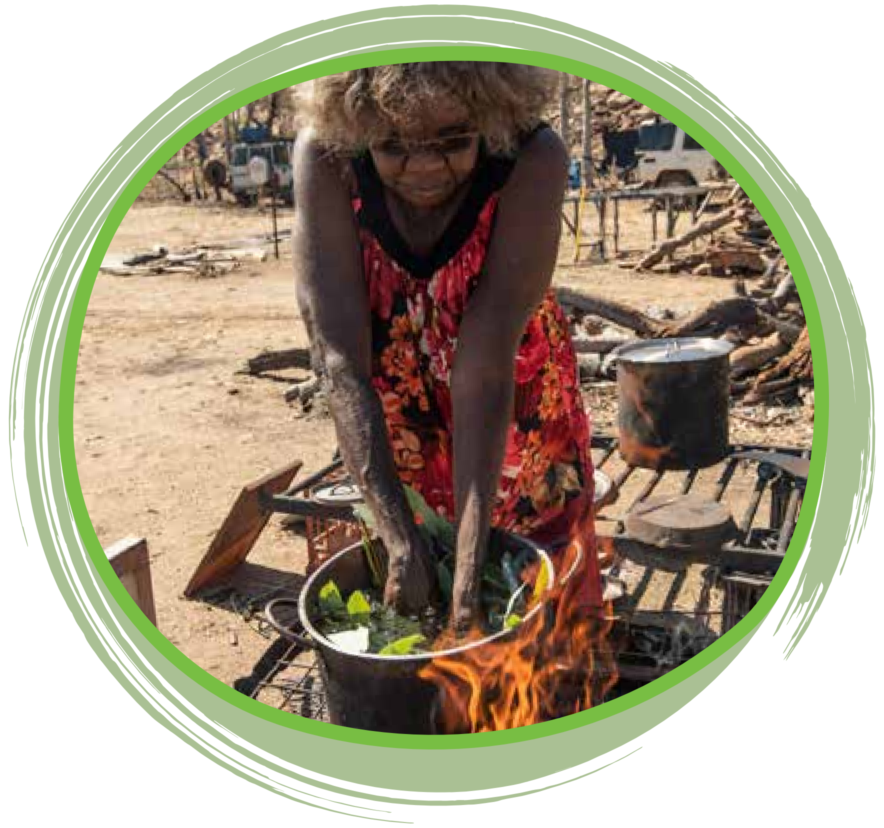

We can also use these to have a bogey (bath).

First, we boil up the leaves, grasses and bark until the water turns green and the smell is strong.

Berrembi men'gawoom nyirrega-girrem.

Dambi ganarram, miloowoom doo bernngam yamberriyilinya-ngarri goorrngan bajalarrin, jaa yamberriyilinya marnen, dam goorrngam werrgal-ngarri woomberriyinbe ngard-ngarri birrirn bagarrjim.

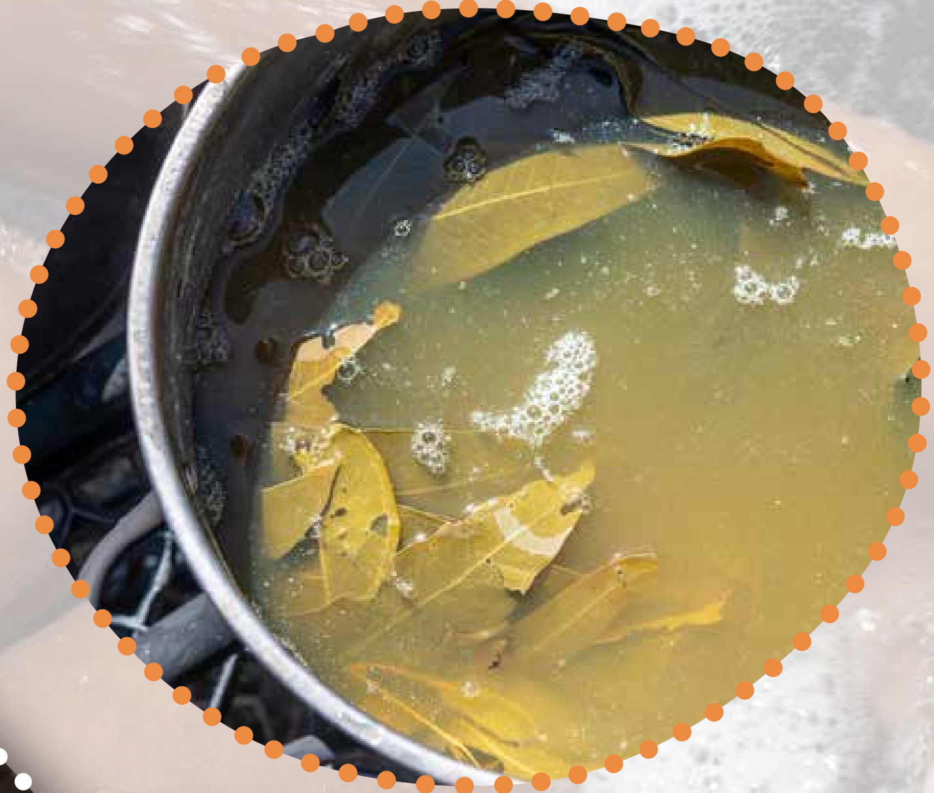

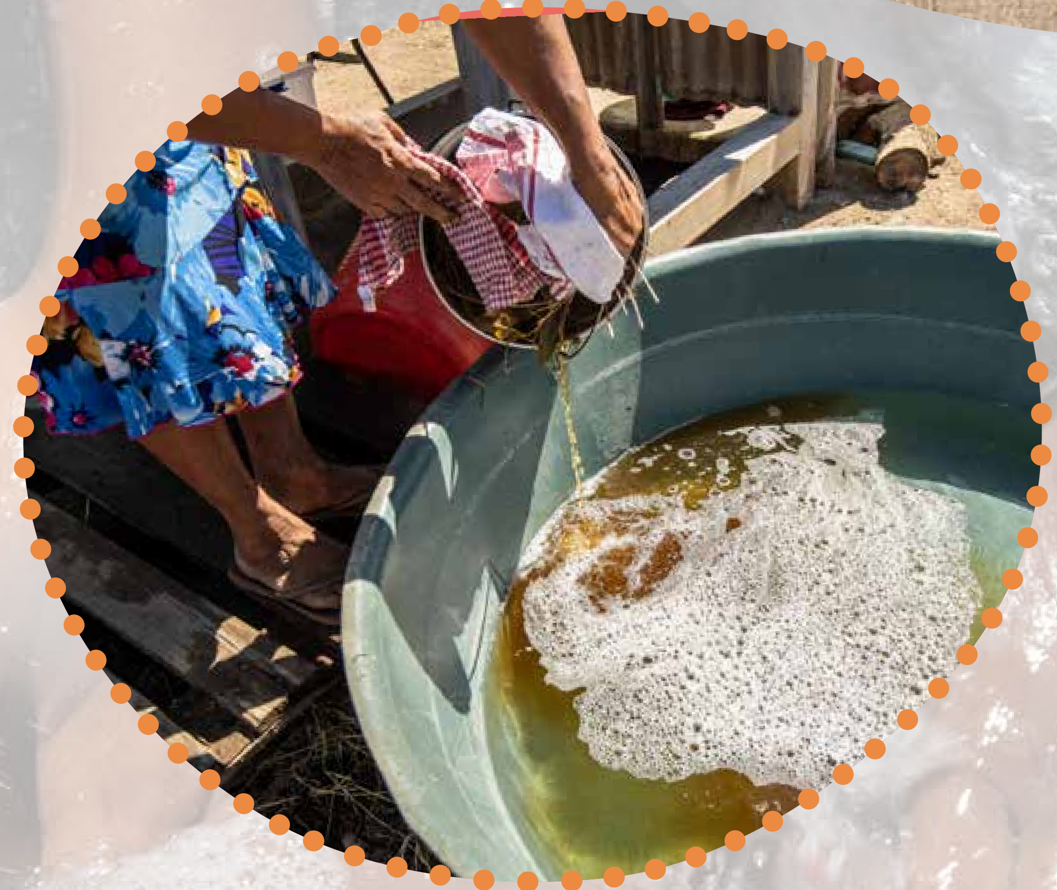

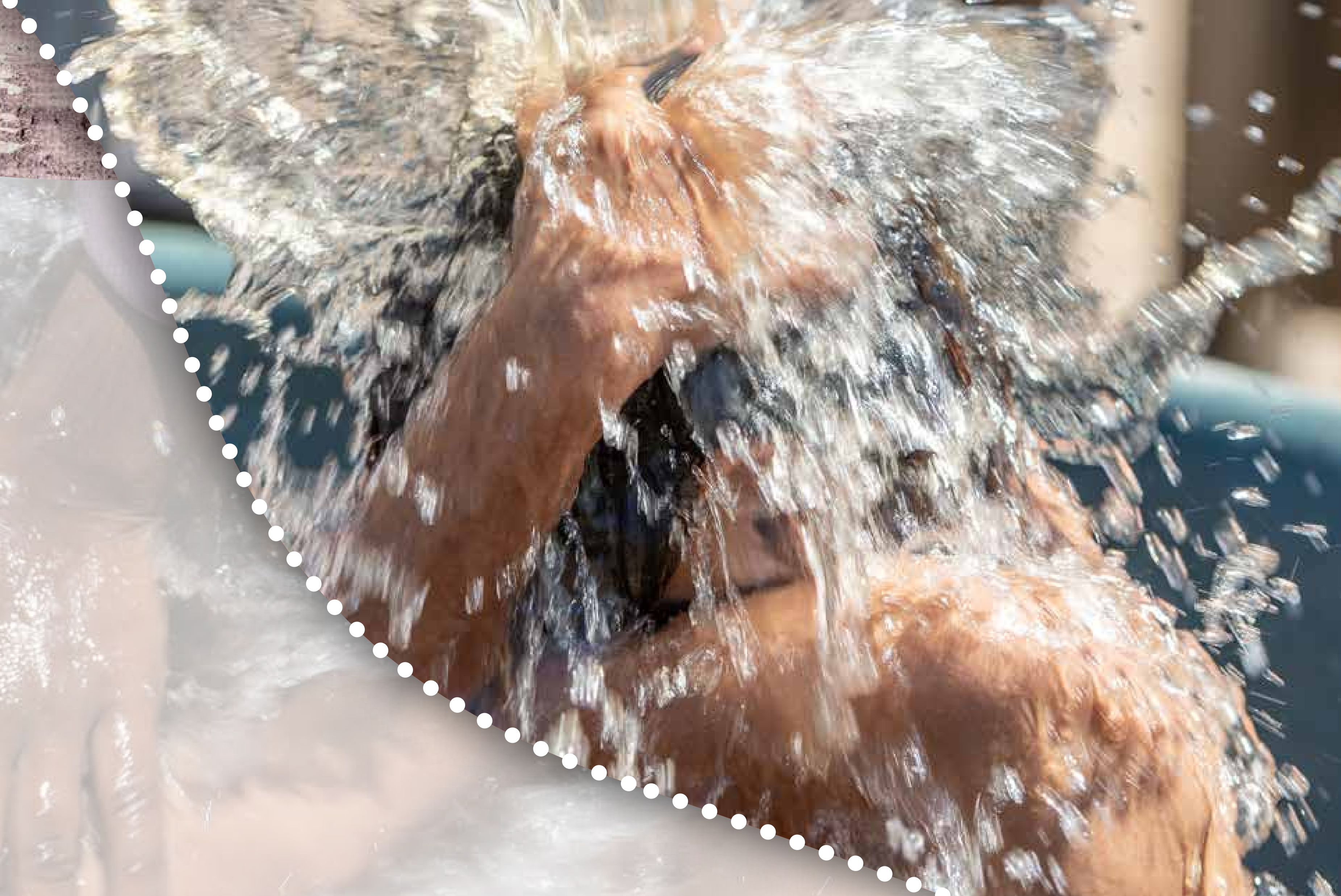

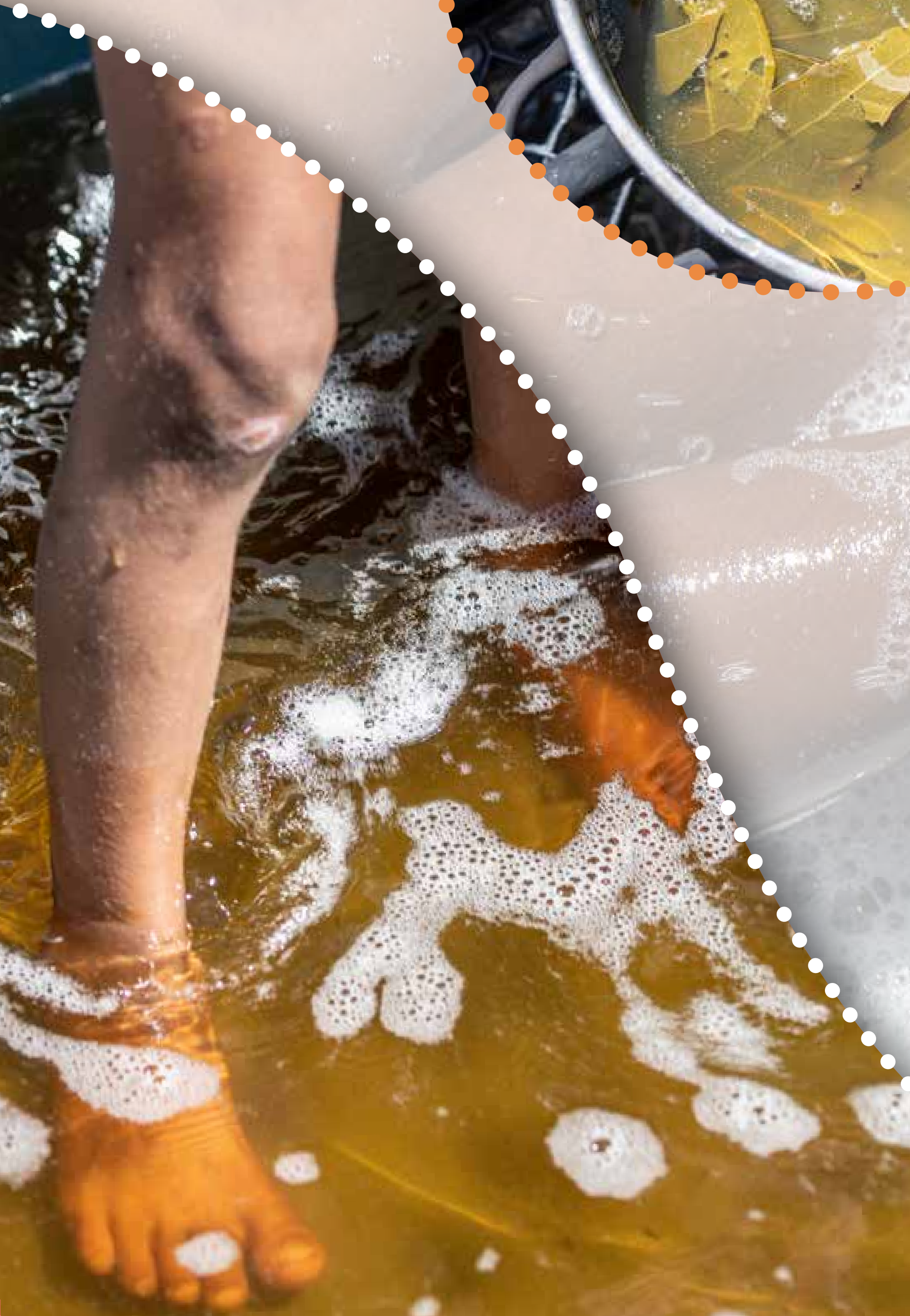

We let it cool for a bit, then put the liquid in the bath with water.

We pour the water over our heads and wash ourselves all over.

Gawilig yambirrimnya, gawili-ngarri woomberriyinbe, yambirriliyinya nawarran bajalarrin nyirrega-girrem.

Mamag yarremiyanya goonggooloon doo garajen.

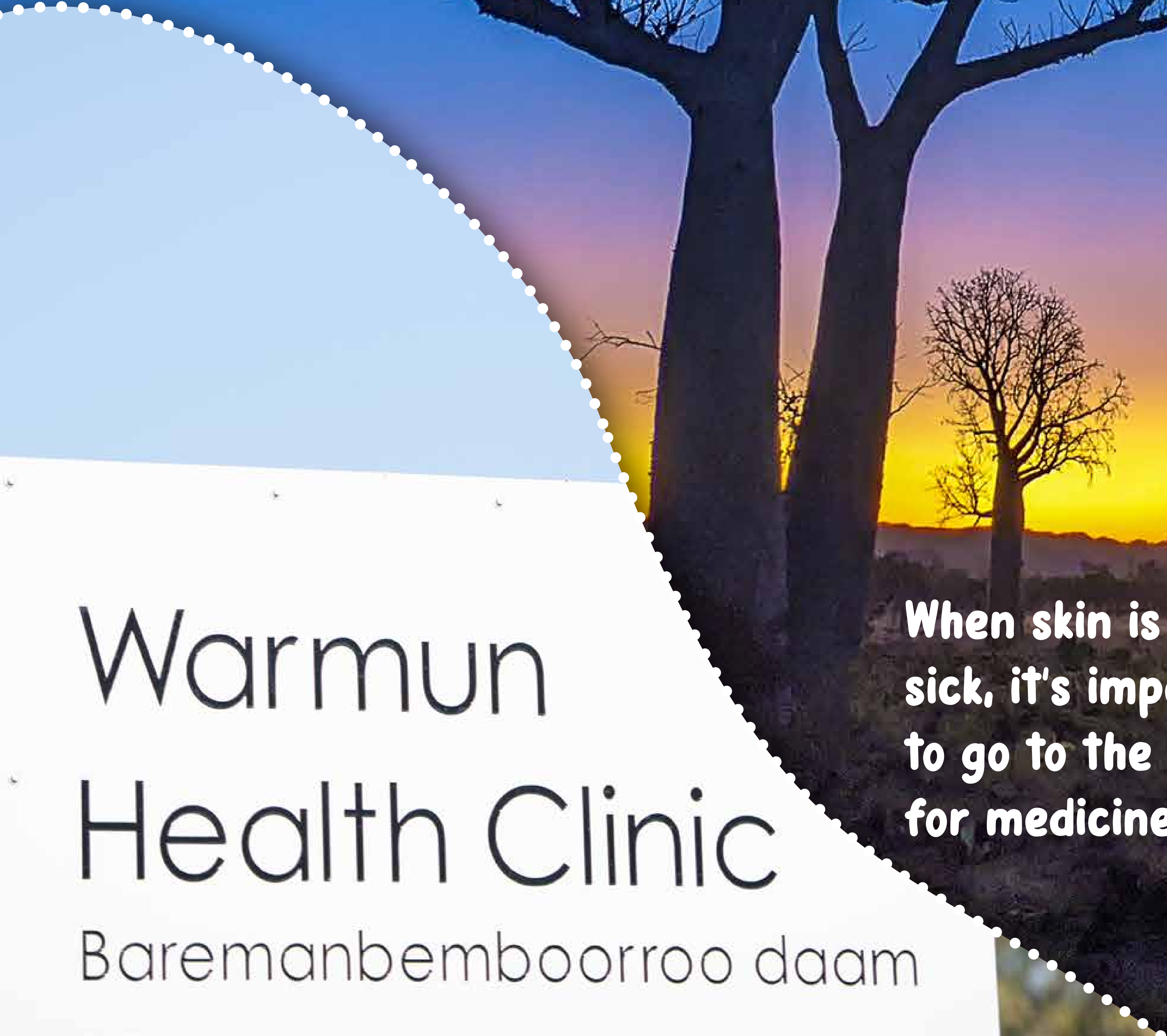

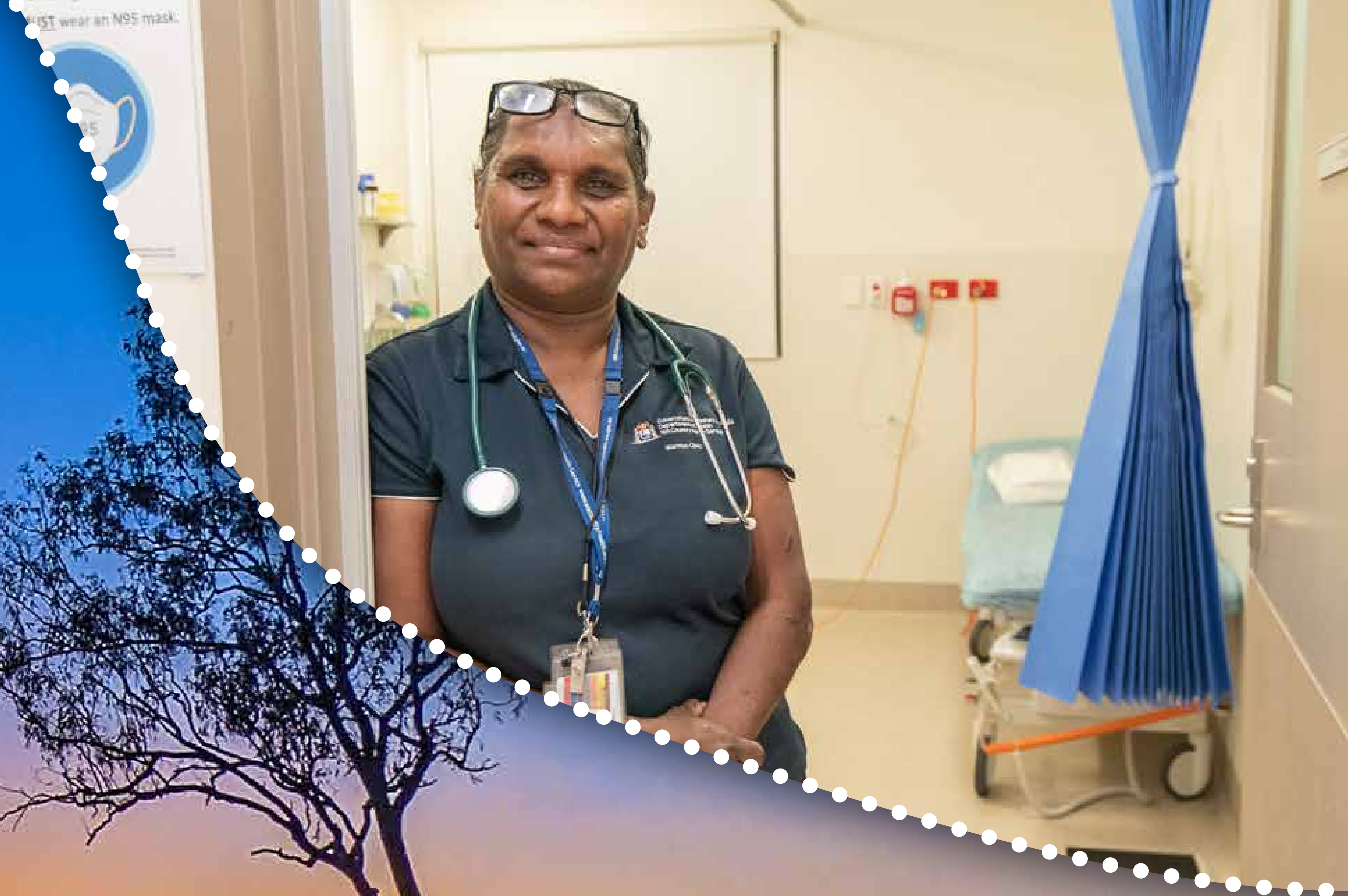

When skin is really sick, it's important to go to the clinic for medicine.

Dambi wajawoorroo warrernbe-ngarri woomberriyinbe, biyarra nyangoobam-birri men'gawoog-girrem.

For skin sores, we can go to the clinic for a medicine called antibiotics.

There are two ways to have this medicine. We can swallow the medicine in a tablet or drink.

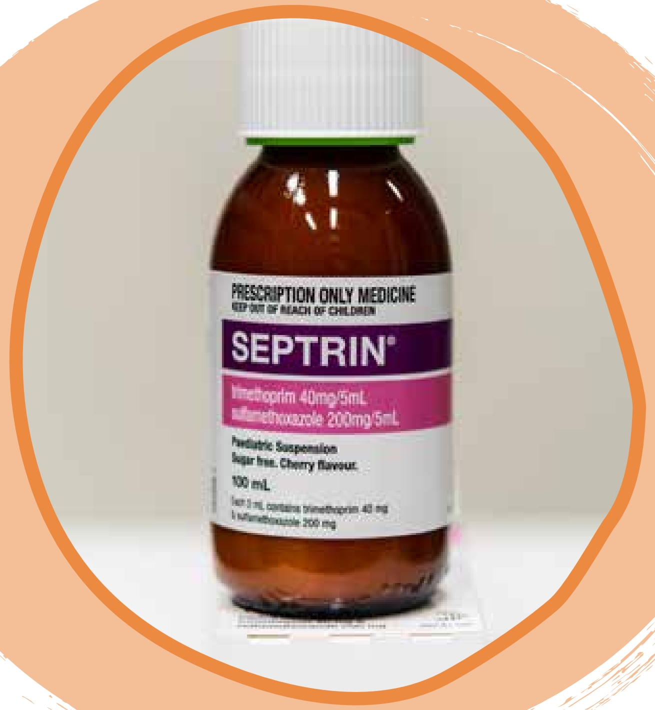

We need to take the medicine every day for a few days exactly like the clinic tells us, to kill the bugs in the sores.

Gajim-boorroo dam yarraan-birri nyangoobam dam men'gawoog-girrem garij-ngarri bemberremenbe yendi-bayiyodig.

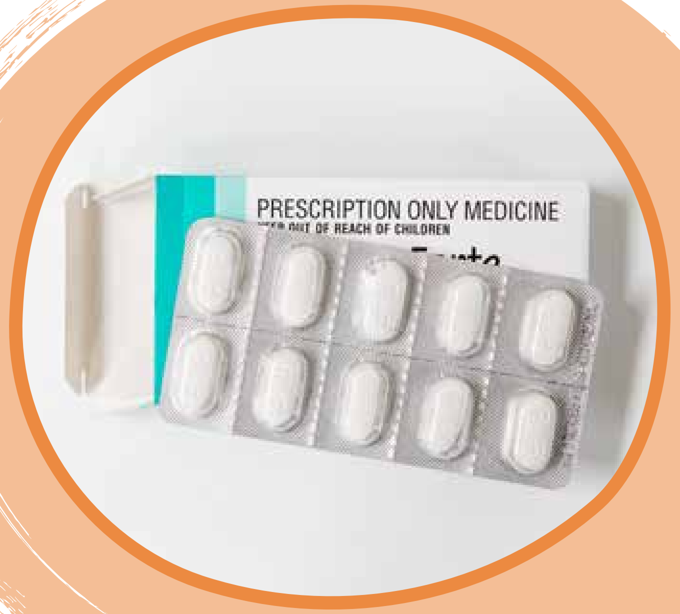

Dambi men'gawoog-girrem bangarinybem. Ngeltheg yamberriyinya dambi men'gawoog-girrem, ngoorloog-wanyji goorrngam-birri.

Nyangoobam waj-garri boorroorn-yarri dam-boorroo gajim, yambirrijande wayiniya gerrij, thed-girrem dam wanyagem gajin-yarri.

The other way to take the medicine is in a needle. Sometimes the needle can be scary, but your family will be there for you.

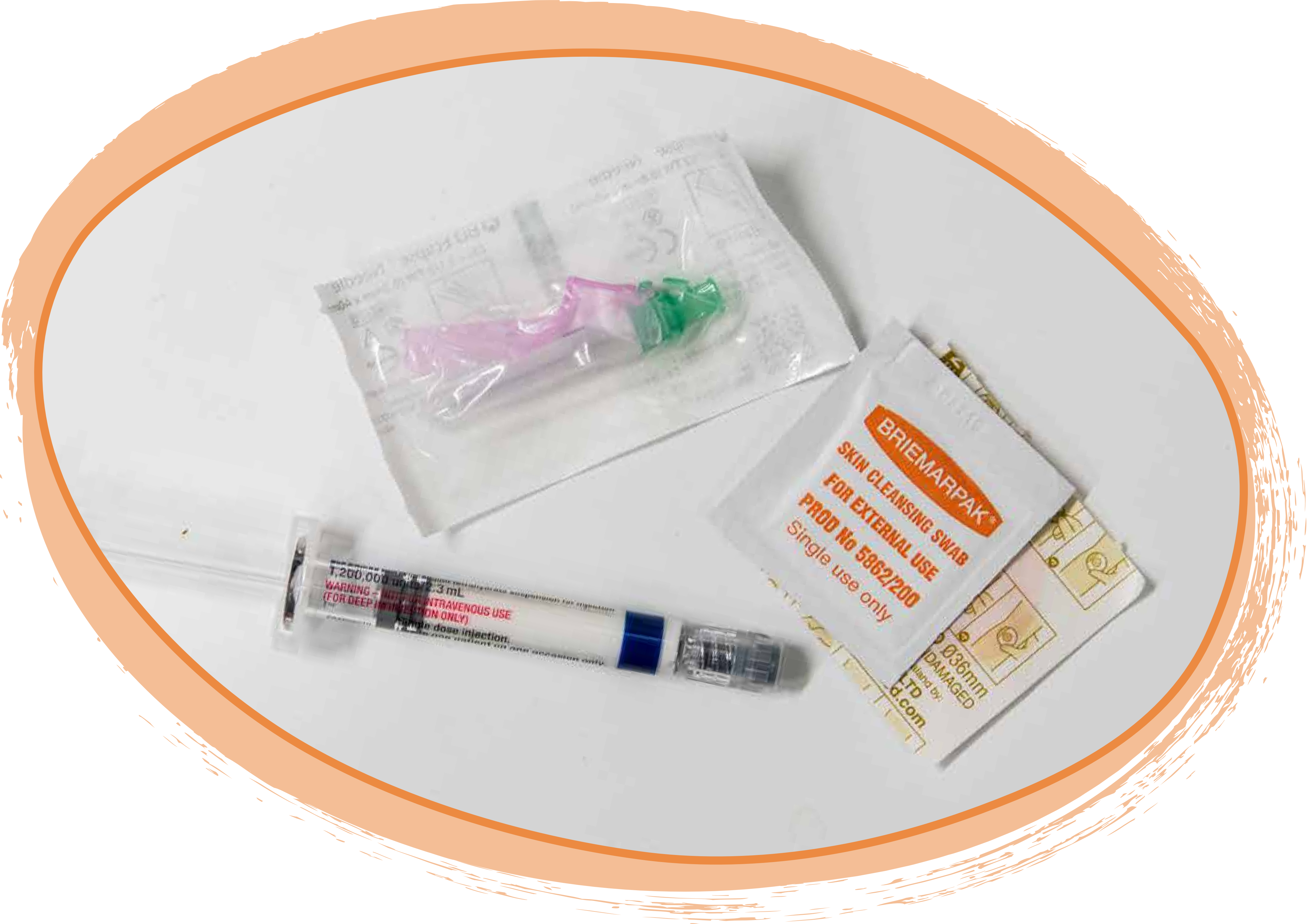

Yagenge-biny dam men'gawoog-girrem mindemgajim yimberriyin-yoowoo. Dambi mindemgajim jilbagenhawoom wanyji, nyingiyanyem ngaboom doo gooram birriyan-nenggoowa.

For scabies, we can go to the clinic for different medicine. There are two ways to treat scabies. One way is a cream, the other way is a tablet.

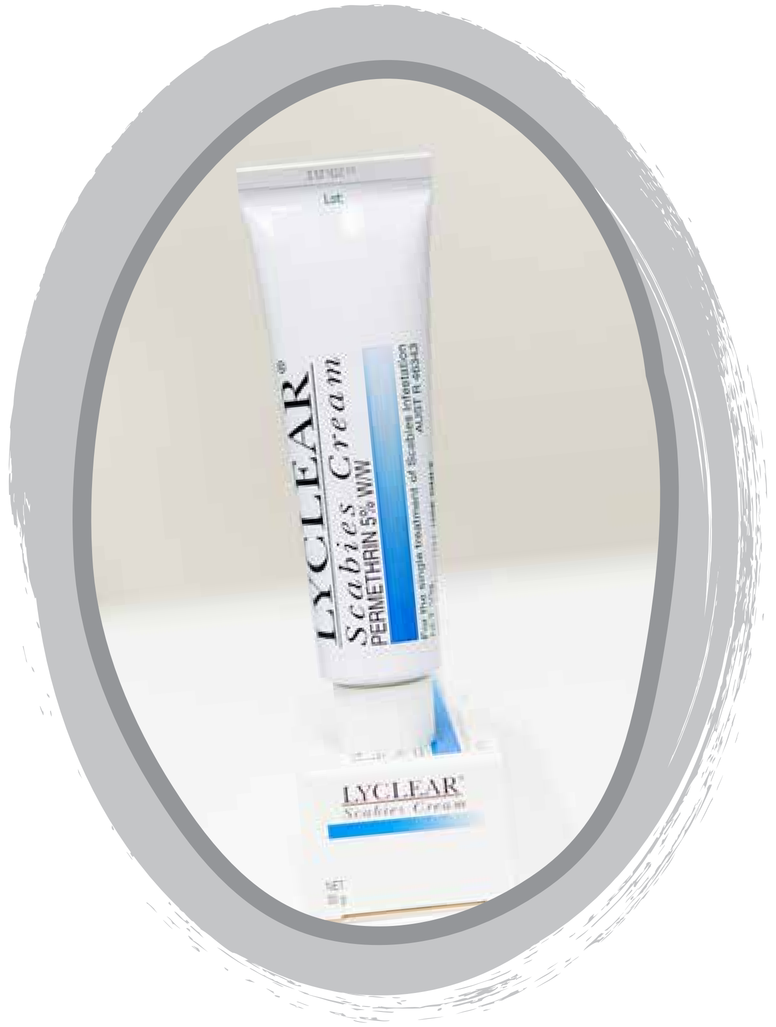

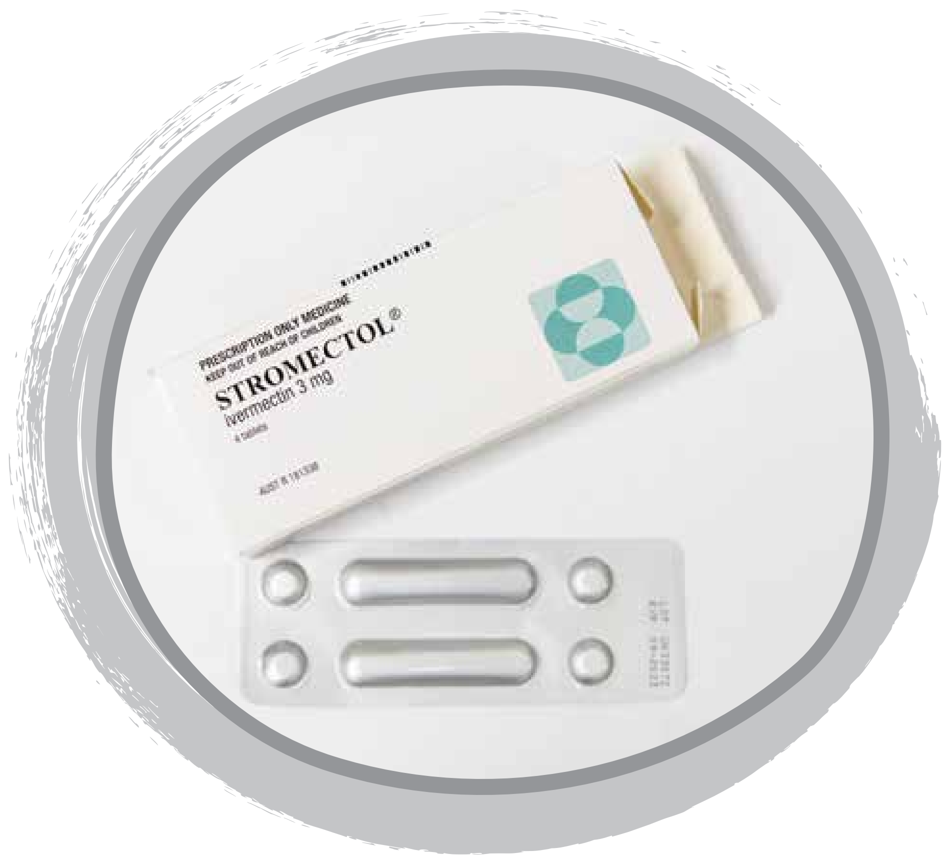

Dan nginjende-ngarri-yarri wajawoorroo, yarraan-birri nyangoobam men'gawoog-girrem. Bangarinybam yamberremoorloonya men'gawoog-girrem dam gajim. Jirrawoombi nyoon-nyoon-girrem dam, jirrawoom dam yagengem ngoorloog-goorrnga-baya.

We have to take either medicine on the first day we have scabies and again one week later.

Girr-girr-ngarri yarremiyanya yarraan-birri nyangoobam dam-boorroo men'gawoog-girrem, ngoorloog yarrern warangganda biri-nyaliny gerawarlen.

Scabies bugs can live in the house and spread to people we live with. That's why everyone in our house needs medicine to stop the bugs camping in their skin.

Dany wanyaginy nginji-ngarri mayaroon, ngiwiyan-birri dam yagengarram boorroonboo-ngarri dan mayaroon. Wayinigana boorroonboo-ngarri mayaroon, bemberrembe dam men'gawoog-girrem.

Washing clothes, towels and bedding is also very important to stop the scabies bugs.

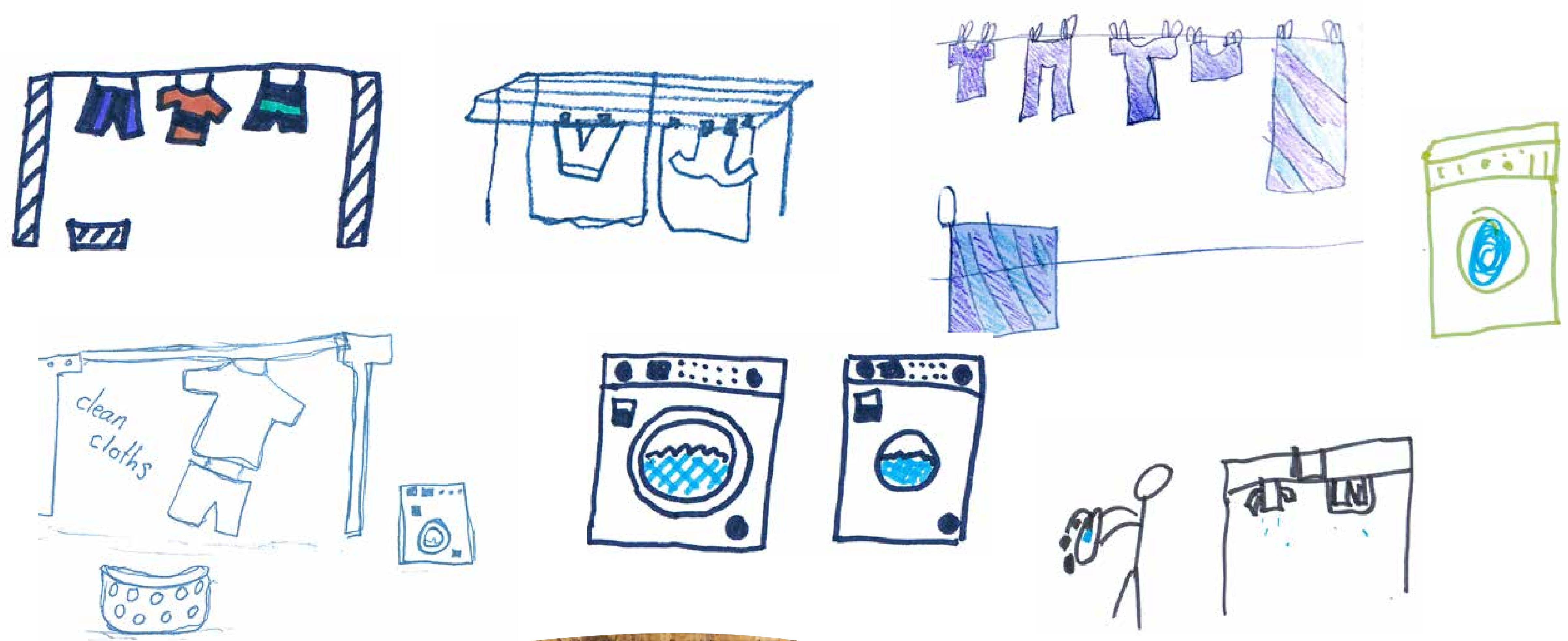

Loogoorr yamberremnya wiremgajim, banjalgbe dam, thoowoo-thoowoom thed-girrem dany wanyageny nyinggijany nginji-ngarri-birri.

There are things we can do every day to keep skin healthy, clean and strong.

Nyirrega yarrern derranden doo mendowoon men'gawoog-girrem wajawoorroo bagarrjig-girrem.

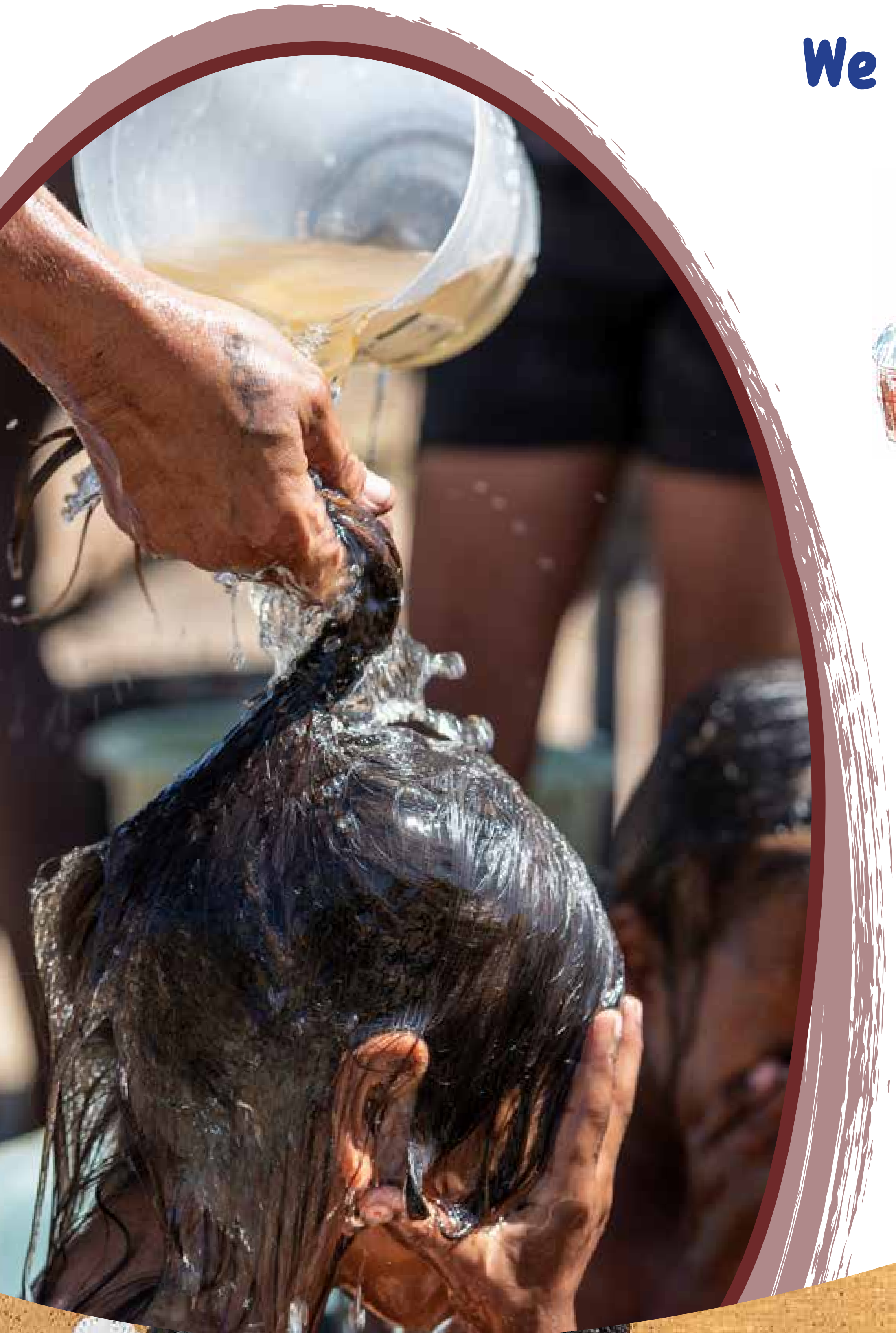

We can wash hands with soap.

Loogoorr yarremija marlam nyoomarlim-birri.

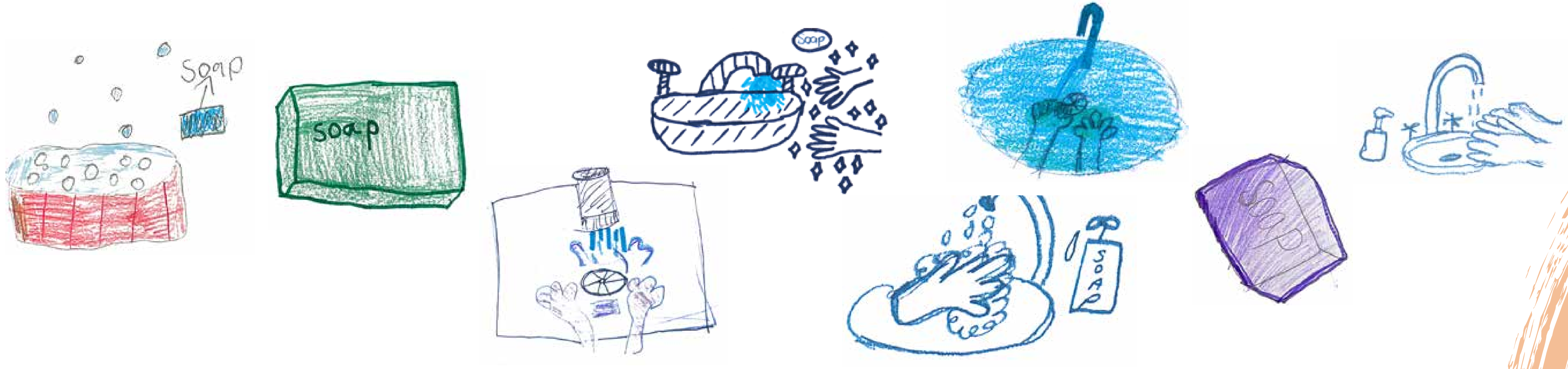

We can have a shower with soap.

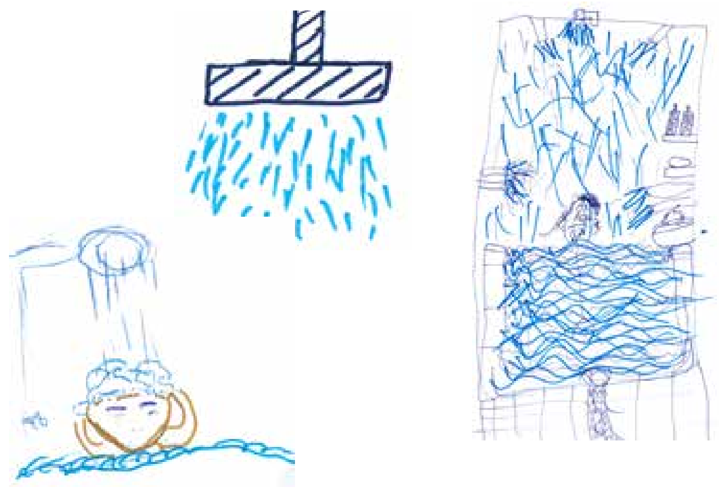

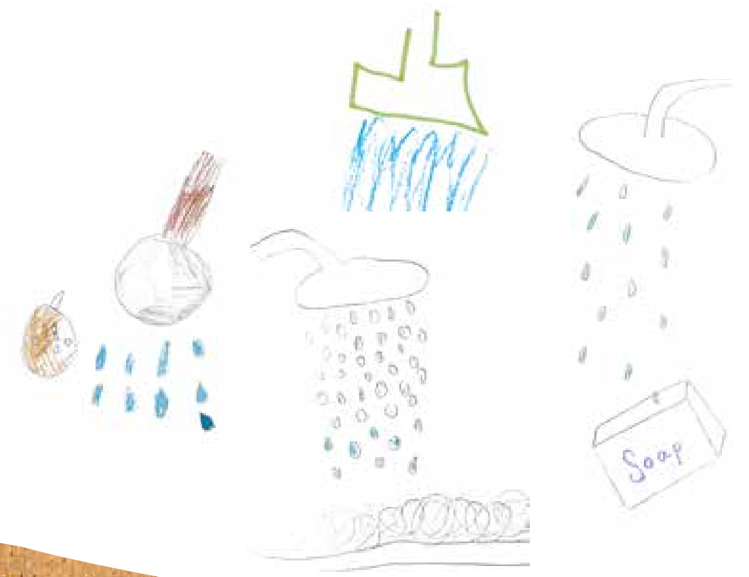

Nyirrega yarrern nyoomarlim-birri.

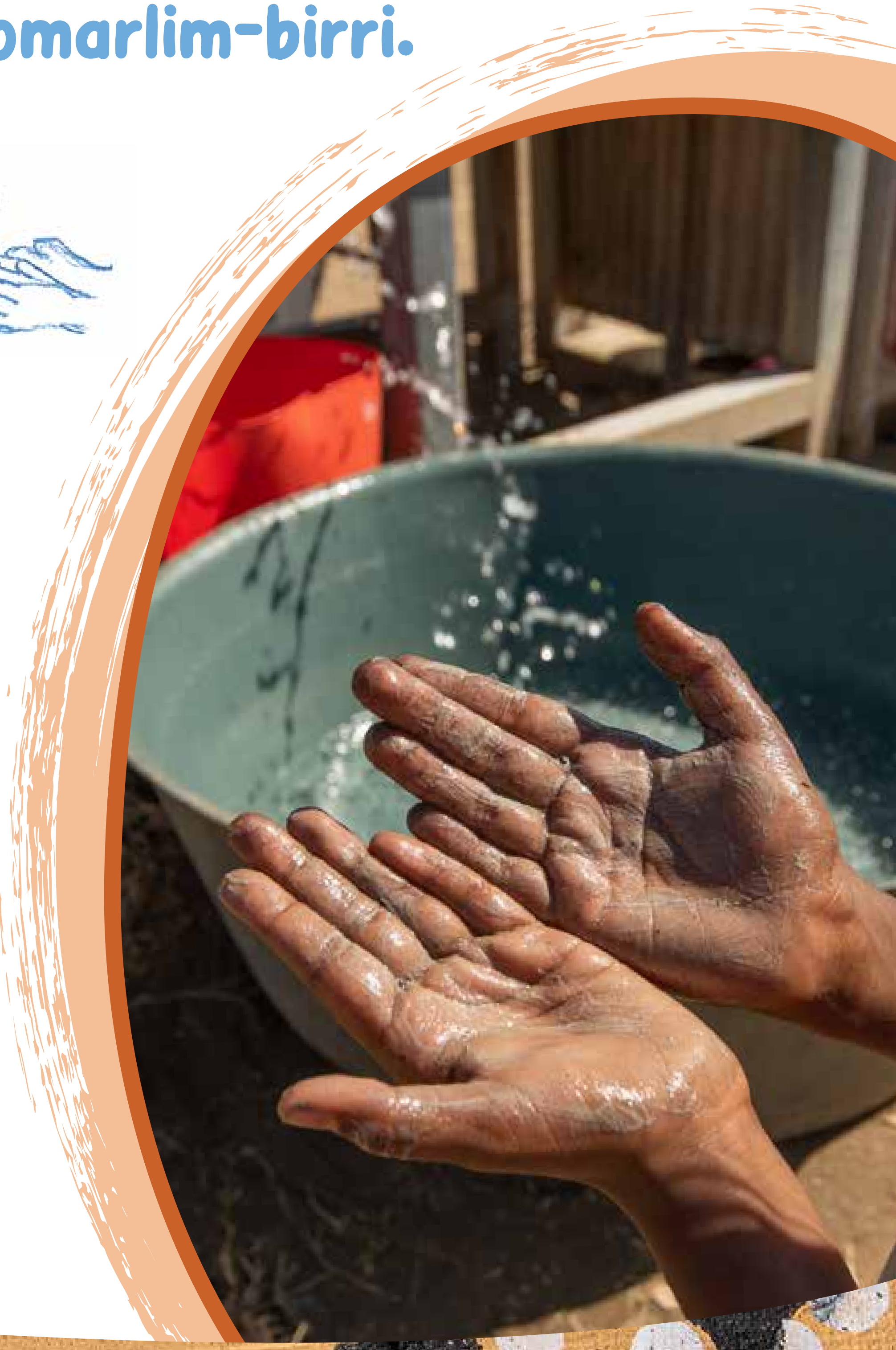

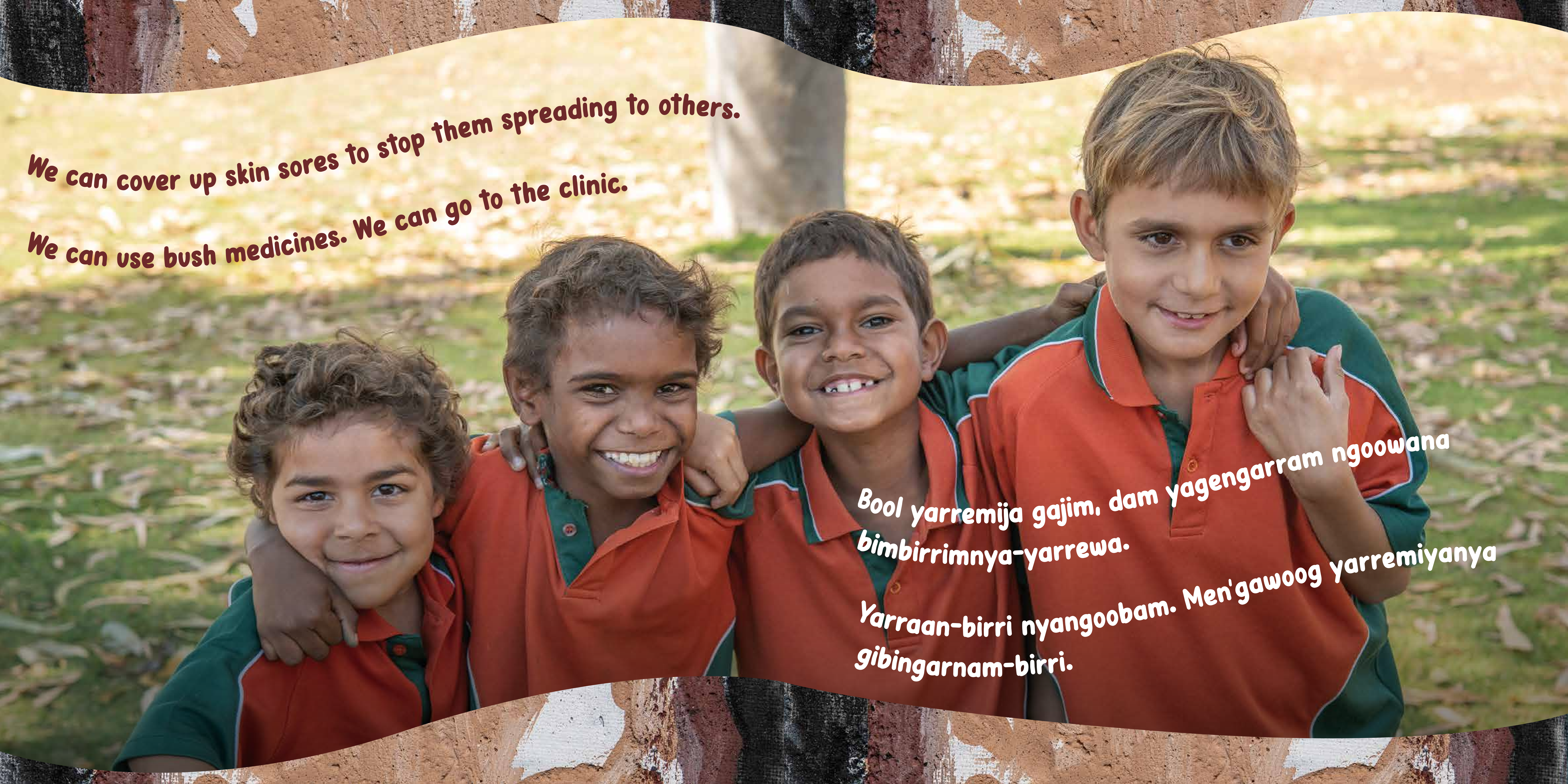

We can cover up skin sores to stop them spreading to others.

We can use bush medicines. We can go to the clinic.

Bool yarremija gajim, dam yagengarram ngoowana bimbirrimnya-yarrewa.

Yarraan-birri nyangoobam. Men'gawoog yarremiyanya gibingarnam-birri.

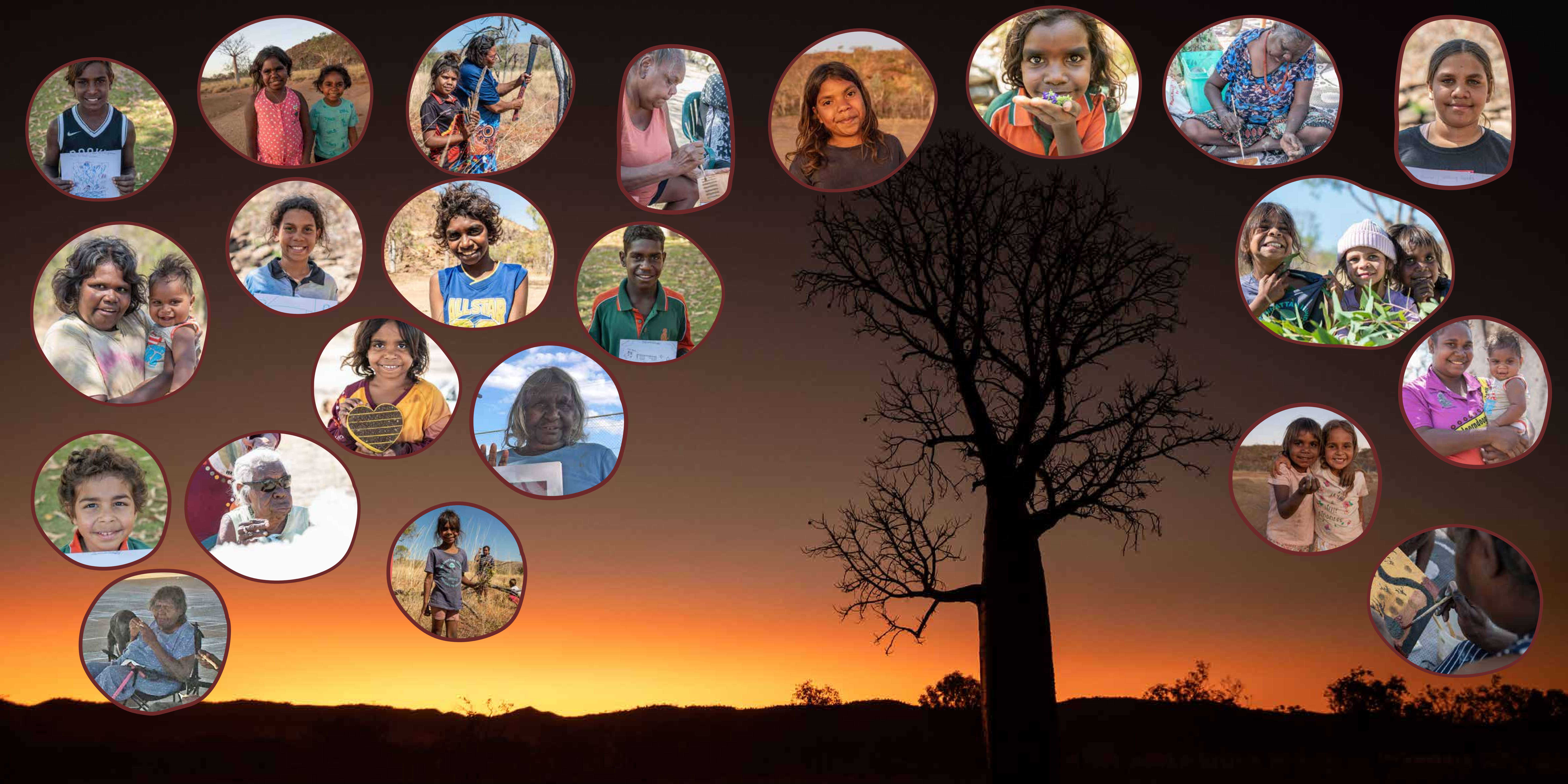
ALLSTAR

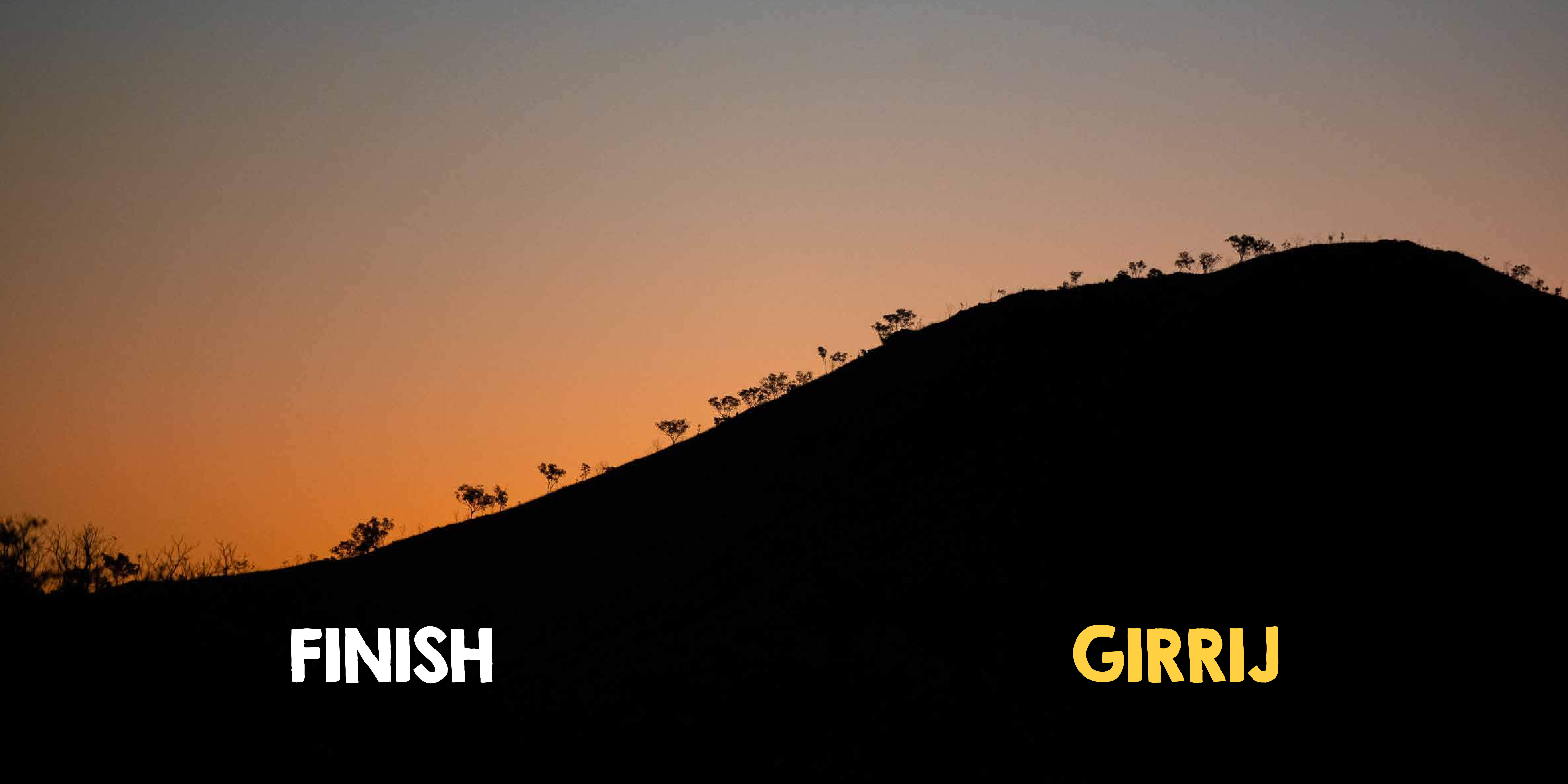

FINISH

GIRRIJ

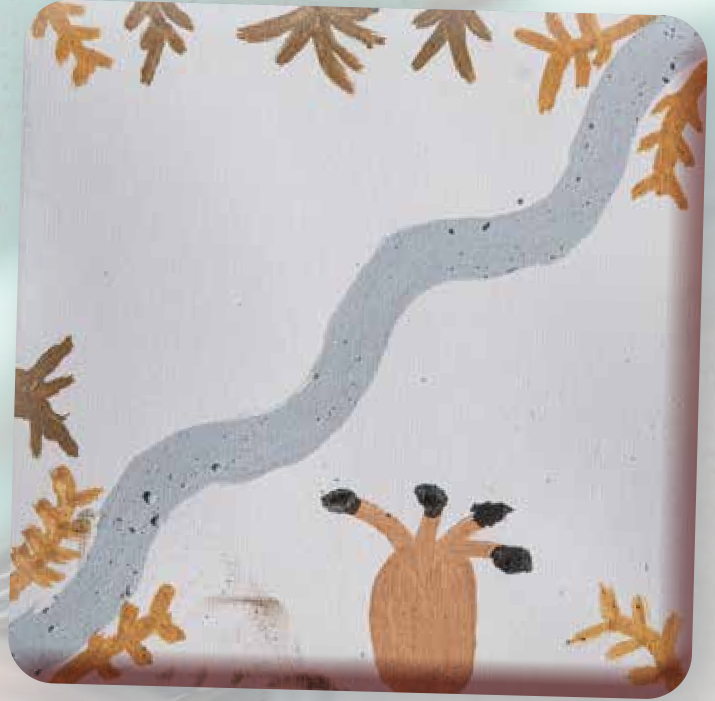

LORRAINE DAYLIGHT

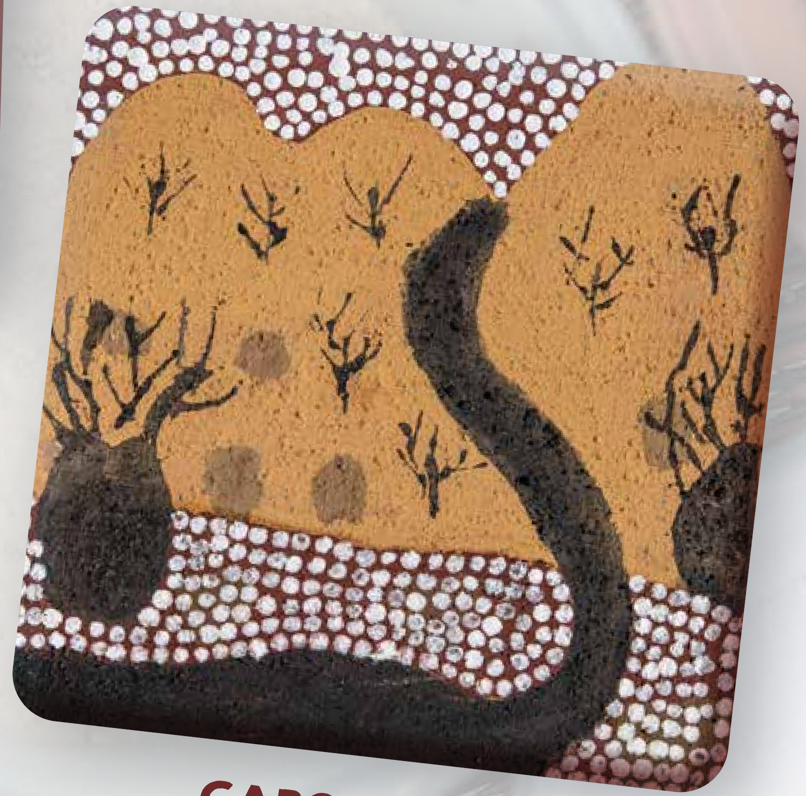

CAROL JULI

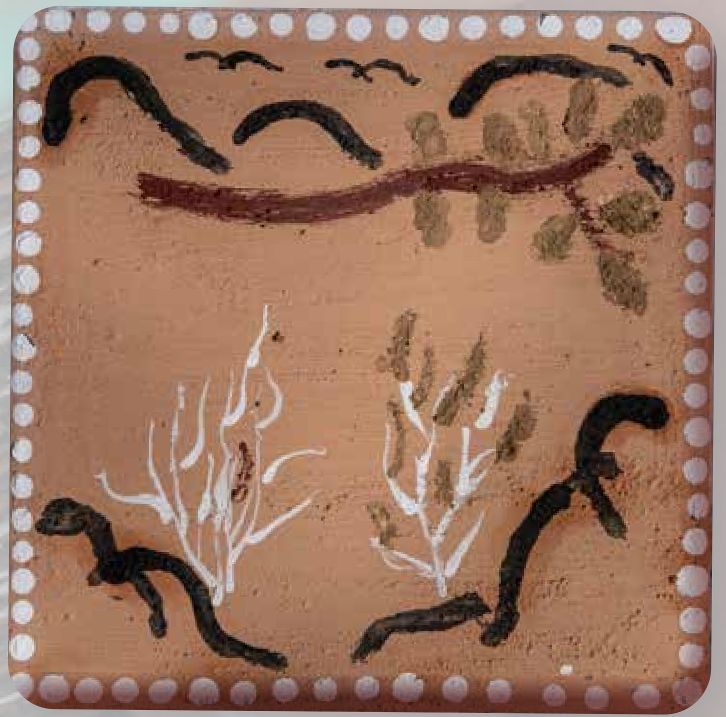

KAREN NUNGATCHA

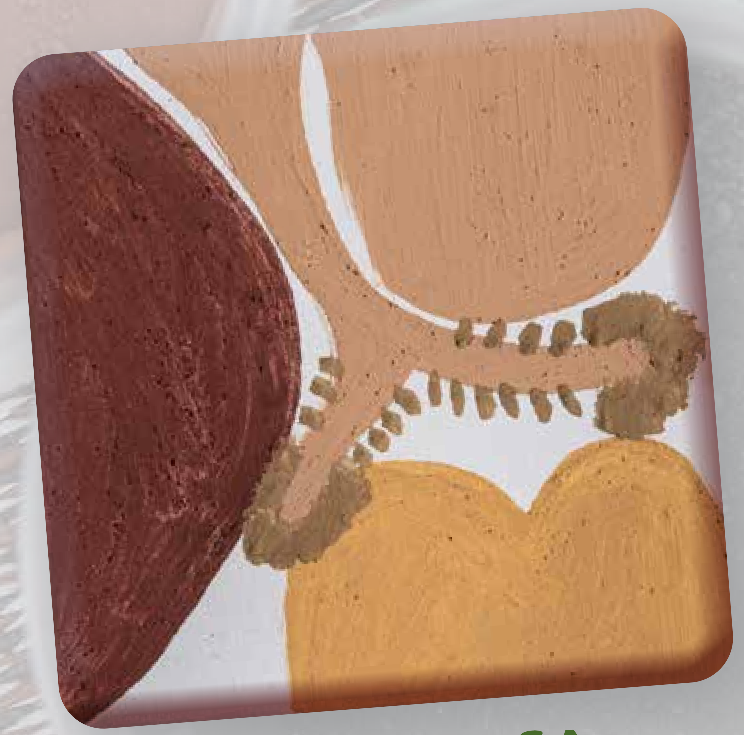

JANE YALUNGA

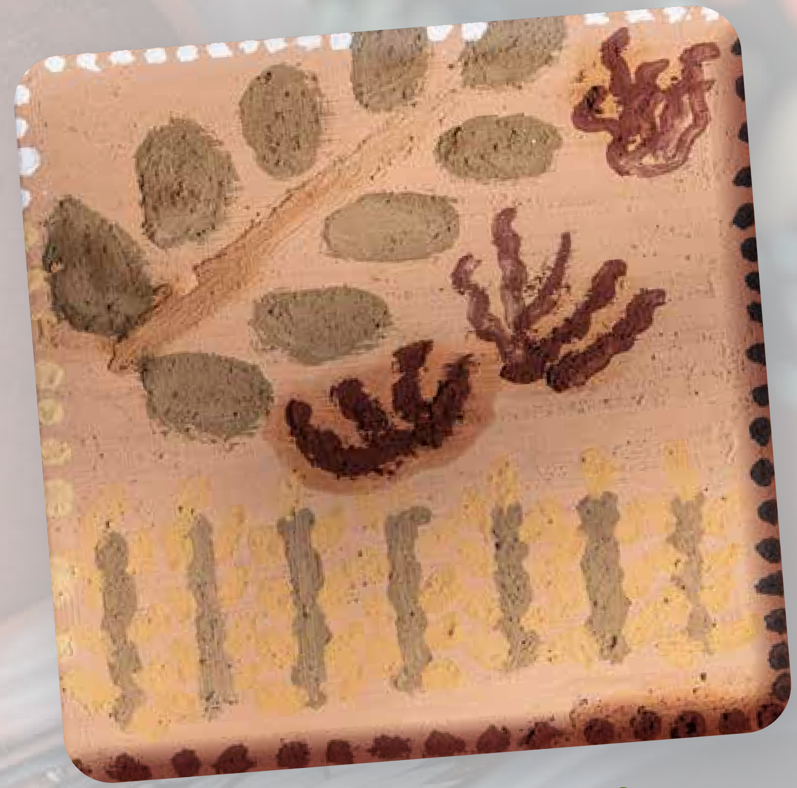

SYLVIA THOMAS

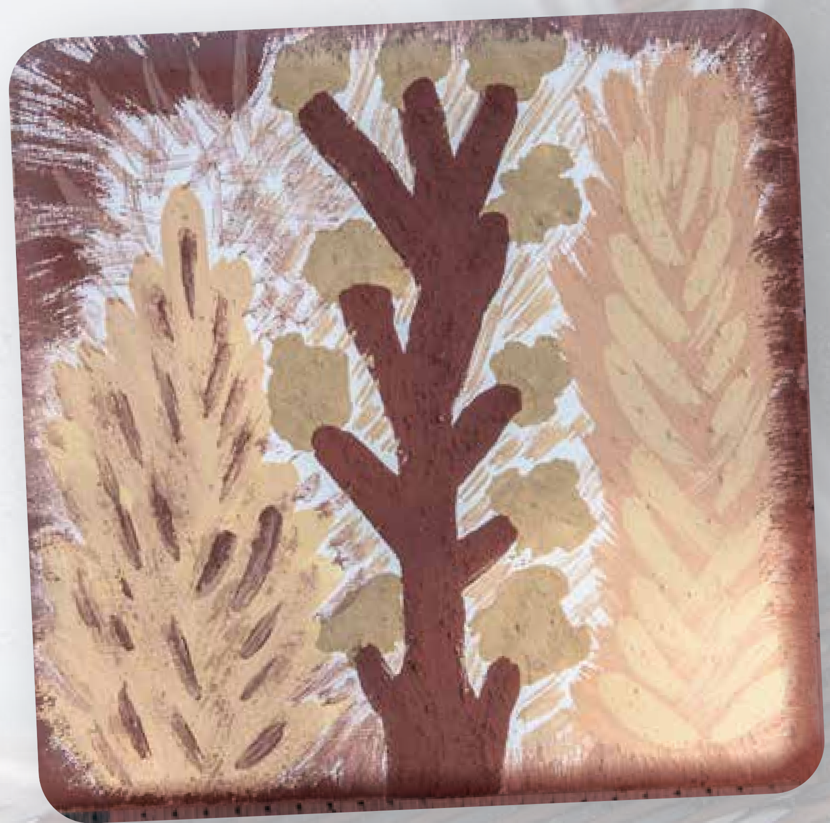

MADELINE PURDIE

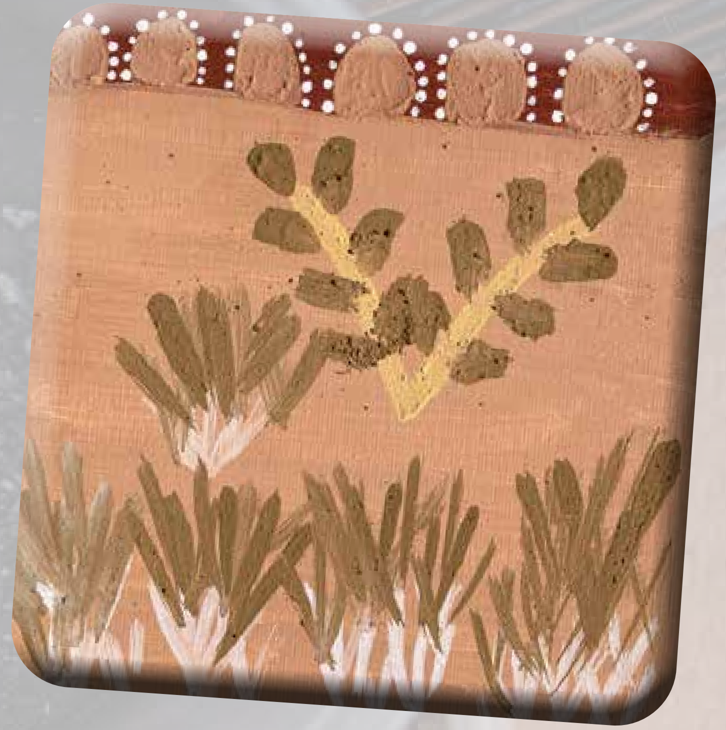

ROCHELLE PERIS

Background

The See, Treat, Prevent Skin Sores and Scabies (SToP) Trial is a shared project between researchers, service providers and communities to support skin health. In the early stages of the SToP Trial (2019 – 2021), the SToP Trial team sat with Warmun Elders and community members to understand the best way to promote healthy skin. The community conceived the idea of a healthy skin storybook featuring both traditional and Western approaches to supporting skin health.

In 2022, the SToP Trial team worked with Warmun community to co-design and co-develop this resource. The team was invited to attend the Two-Way Women's Healing camp, supported by the Warmun Local Drug Action Group, where stories and pictures of traditional bush medicines were shared and recorded. Students participated in skin health lessons organised by the health teacher, where they produced drawings of how they keep their skin strong. Finally, clinic staff members shared information about the clinic medicines commonly used to treat skin infections.

The language, art and traditional knowledge included in this resource is owned by Warmun community. Artists and translators were remunerated and gave permission for their work to be included in this resource.

The resulting resource is aligned with *Keeping Skin Healthy: A Handbook for Community Care Workers* (Pilbara, 2019) and *National Healthy Skin Guidelines* (2018).

The SToP Trial is a collaboration between Telethon Kids Institute, Kimberley Aboriginal Medical Services, WA Country Health Service - Kimberley and Nirrumbuk Environmental Health Services. The SToP Trial team sincerely thanks everyone who was involved in this project.

Ford A, Purdie M, Bray E, Koford F, Malgil R, Bray S, Sibosado S, McRae T, Gibbs T, Major A, Whelan A, Poore A, Thomas HMM, Bowen AC, *Berrembi jarragoo-boorroo wajawoorroo men'gawom Gijam (Gija Healthy Skin Story),* Telethon Kids Institute, Perth 2022

Copyright Telethon Kids Institute, 2022

Proudly supported by

SToP (See, Treat, Prevent) Skin Sores and Scabies

TELETHON KIDS INSTITUTE Discover. Prevent. Cure.

NIRRUMBUK ENVIRONMENTAL HEALTH & SERVICES

GOVERNMENT OF WESTERN AUSTRALIA SUPPORTER

Keeping lore and culture strong WARMUN

healthway

Australian Government National Health and Medical Research Council

NHMRC

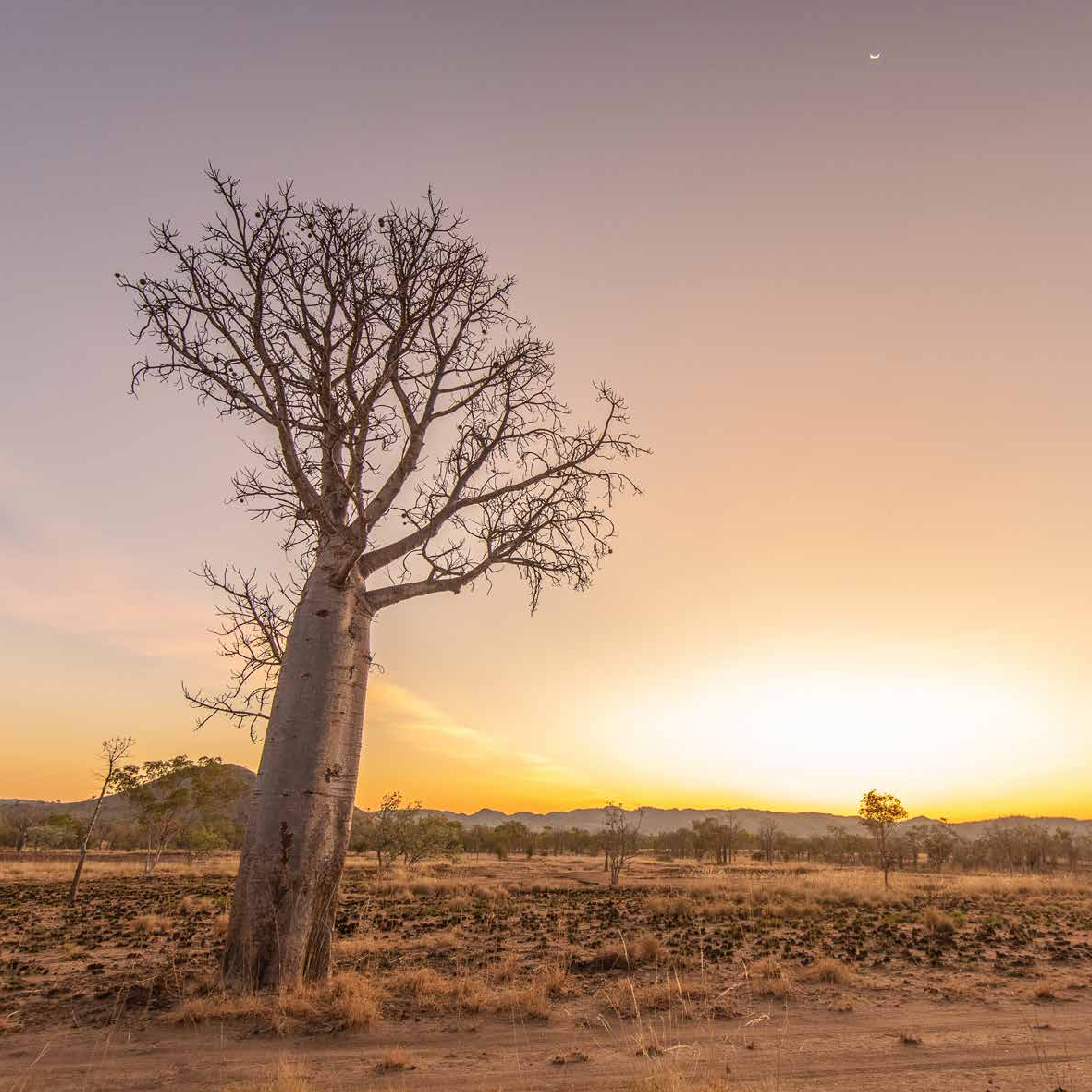

Supplement: Supplementary file 1 — Appendix 1: Berrembi Jarragboo‐boorroo Wajawoorroo Men'gawoom Gijam (Gija Healthy Skin Story). [file HPJA-36-0-s002.pdf]
