# Supplementary material for: Berrembi Jarragboo‐Boorroo Wajawoorroo Men'Gawoom Gijam (Gija Healthy Skin Story): Two‐Way Learning for Healthy Skin
Source: Health Promot J Austr. 2025 Oct 2;36(4):e70111. doi: 10.1002/hpja.70111 (PMC12491125; doi:10.1002/hpja.70111)

Dijan wen wi tokin bela propa good wan skin gota Gija

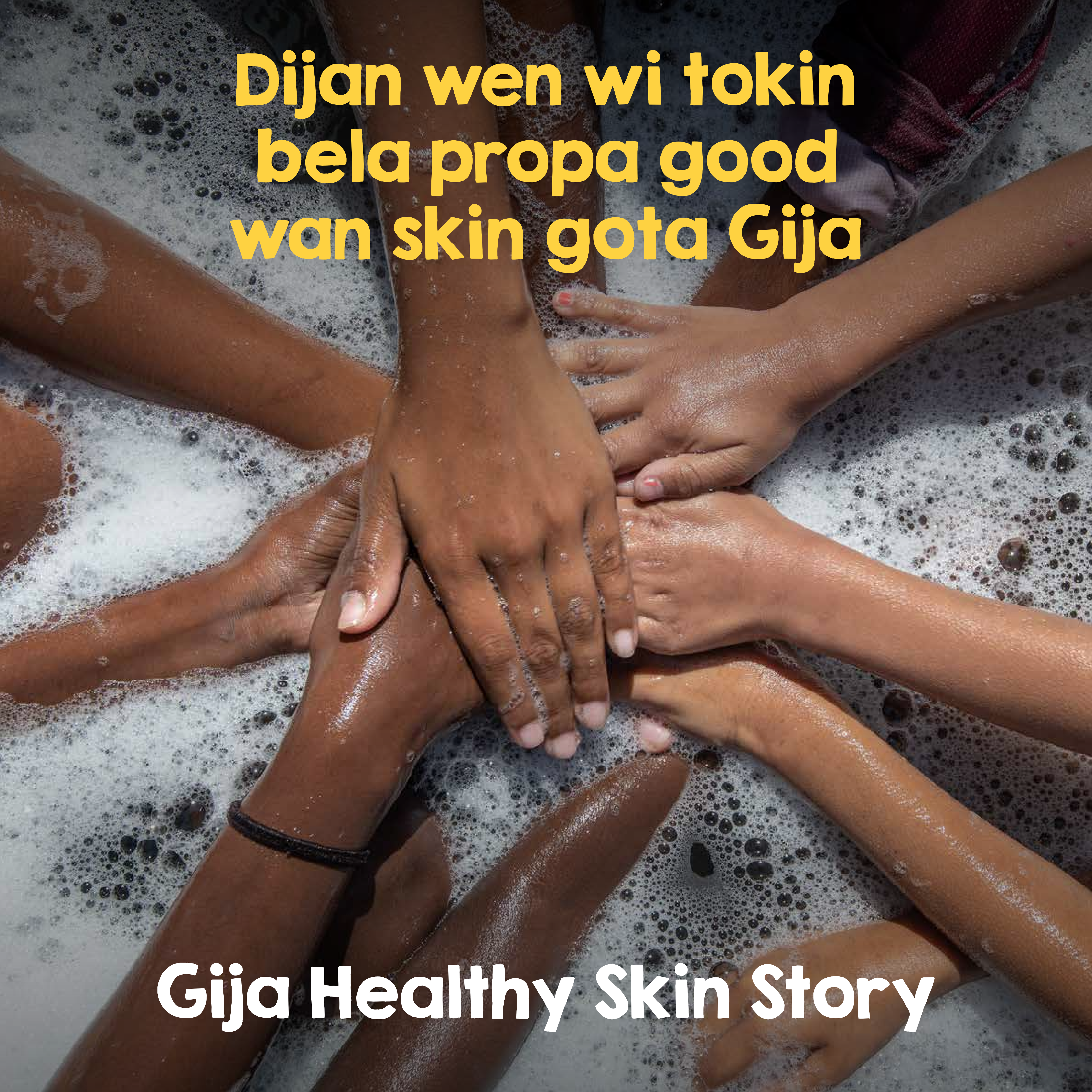

Gija Healthy Skin Story

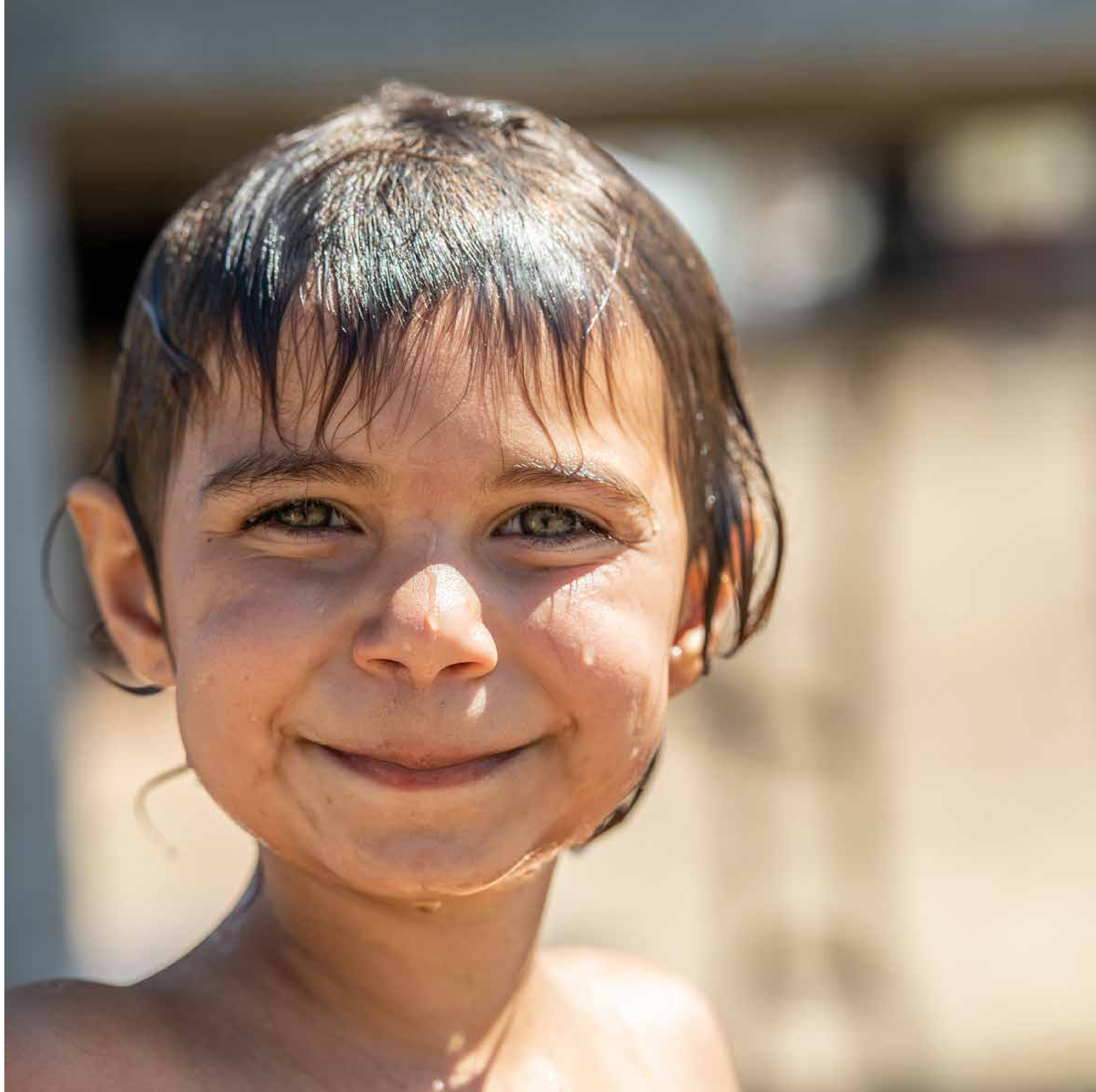

The Gija people are the Traditional Owners of the land and waterways on which this resource was produced. They have a strong, continuous connection to Country and Culture, especially traditional healing practices, and have shared their knowledge and wisdom in this resource so it may be celebrated for generations to come.

The following people were involved in making this resource:

Madeline Purdie and Cherylene Nocketta, who supported the project in Warmun community.

Mary Thomas, Mabel Juli, Shirley Purdie and Eileen Bray, who shared the traditional knowledge included in the resource.

Carol Johnson and Lauren Stone, who supported the project at Ngalangangpum Catholic school.

Madeline Purdie, Karen Nungatcha, Carol Juli, Sylvia Thomas, Lorraine Daylight, Jane Yalunga and Rochelle Peris, who provided the artwork.

Eileen Bray and Frances Kofod, who provided the translation.

Rose Malgil and Sonia Bray, who provided the content for the story.

The children of Warmun community, who provided the drawings.

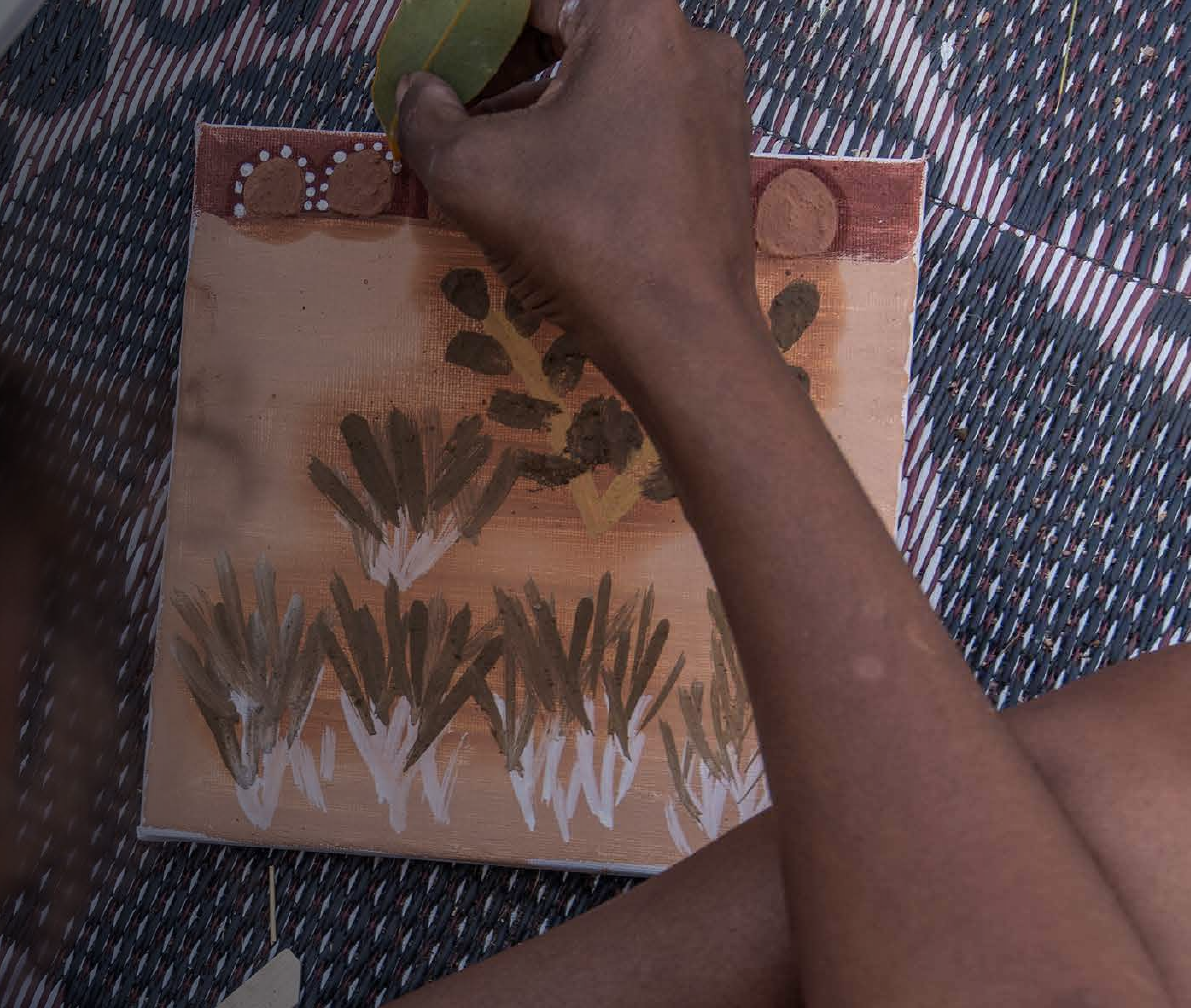

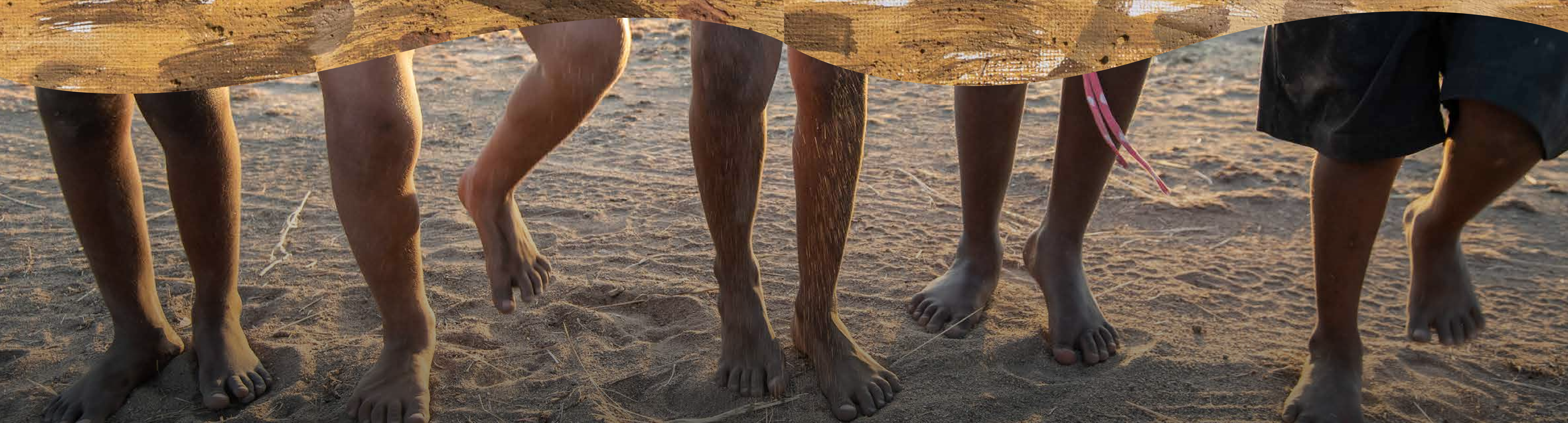

Our skin protects our bodies.

If our skin is sick, our bodies, kidneys and hearts can get sick.

Strong skin means strong bodies, strong kidneys and strong hearts.

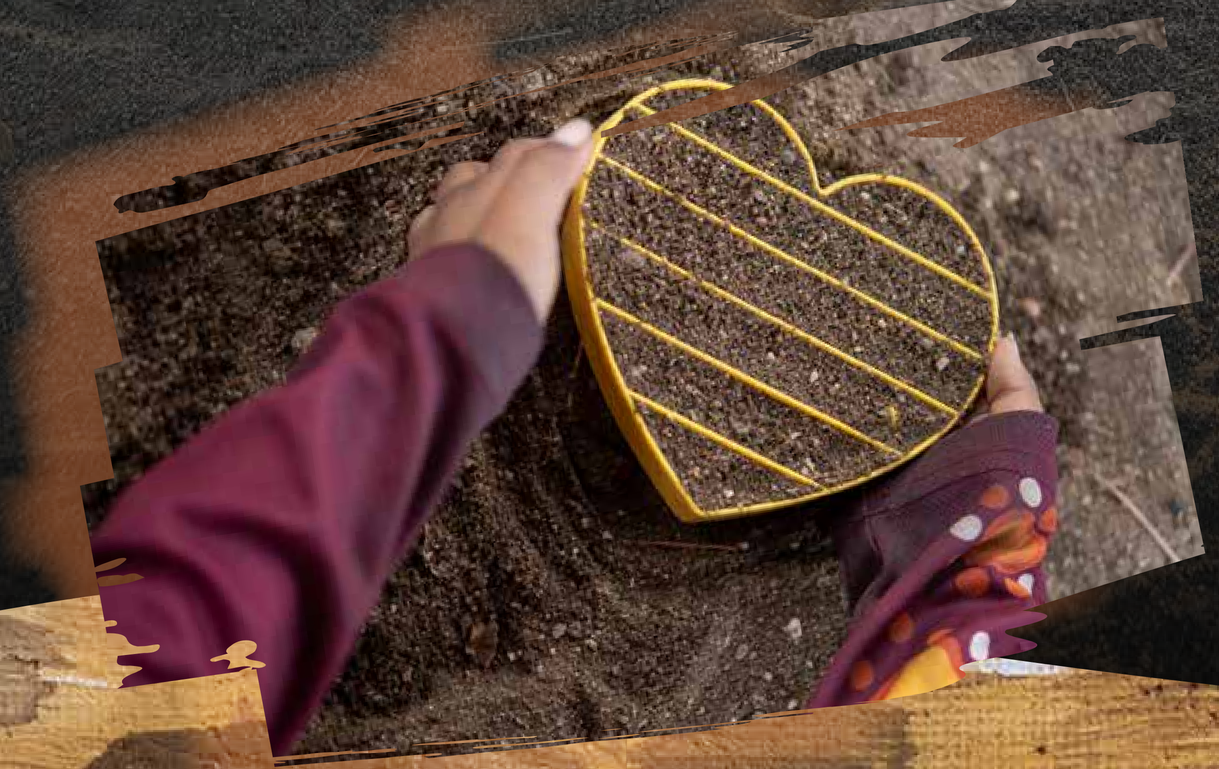

Dijan skin bela wi, im loogafem wi.

Dijan skin bela wi, wen im propa no good wan, im meigem wi sick olrawn la wi haat an la kidni.

Wen that skin bela wi him propa strong wan, bela wi bodi haat an kidni strong wan.

There are lots of different skin sicknesses.

Skin sores, or school sores, are caused by germs called bacteria.

If they're not treated, they can lead to bad diseases like Rheumatic Heart Disease and kidney disease.

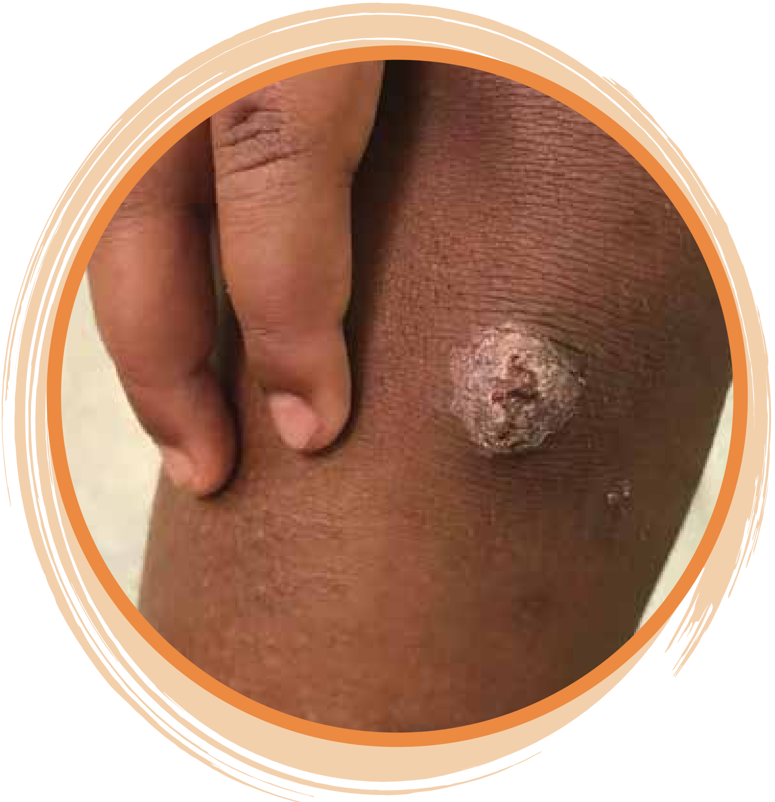

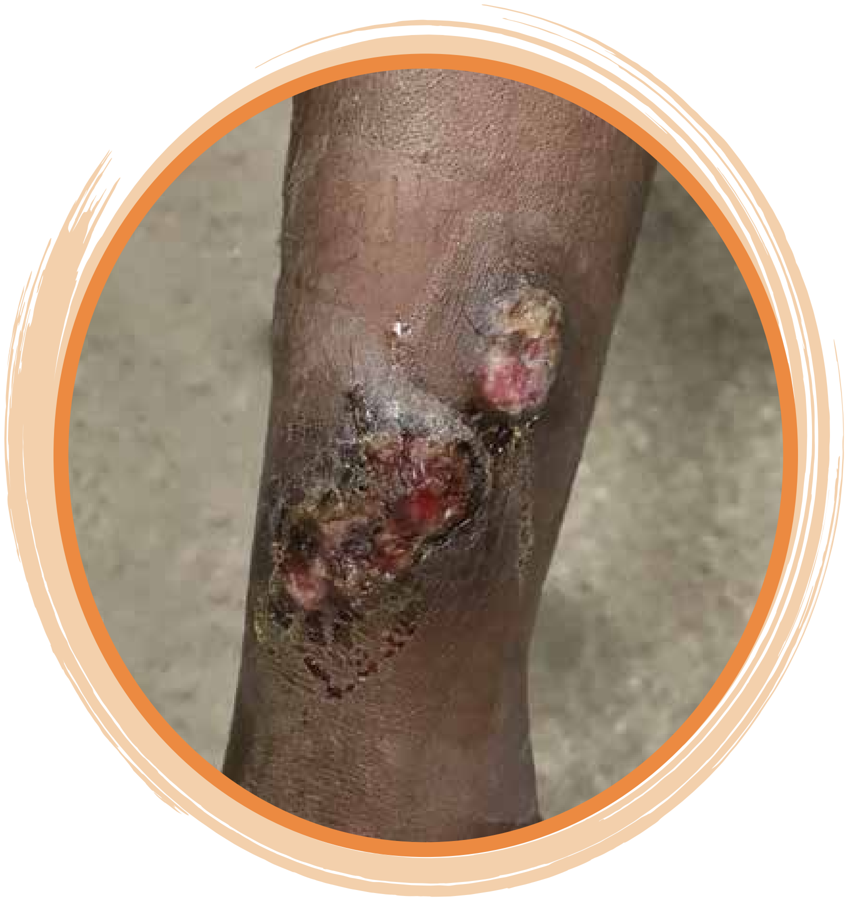

Hi gotem big mob difren kaina siginis bela skin.

Ola sowa wen olabat getembat everiweya, they getem fram samthing lidl wan thing weya hi spredembat la olabat.

If they gaant getem bat medijin bela dijan sowa qwik, thei ken getem Roomati haat and no good wan kidni.

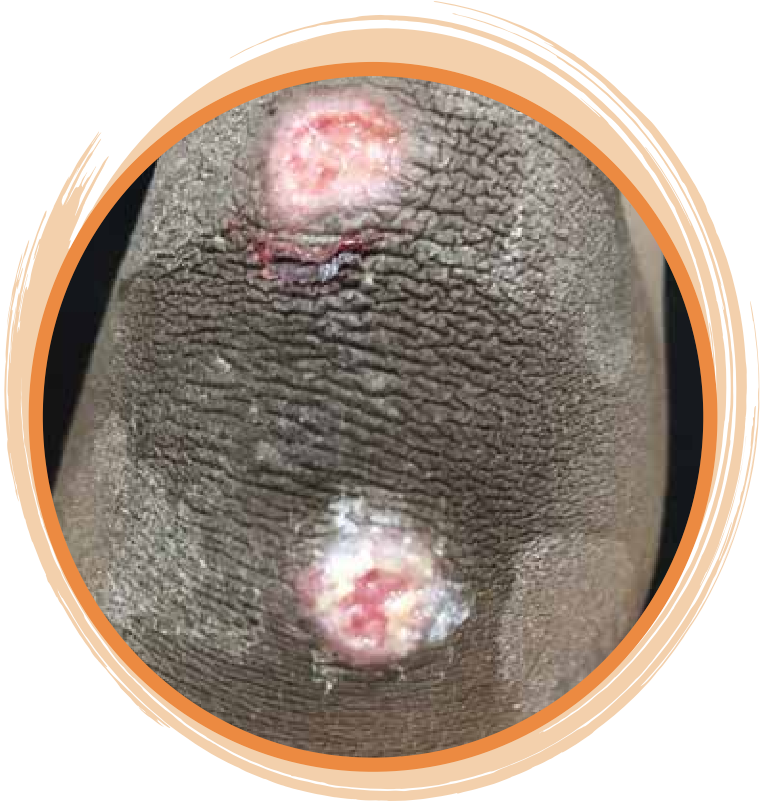

Scabies are cheeky bugs that camp in the skin. They are really itchy and too much scratching can lead to skin sores.

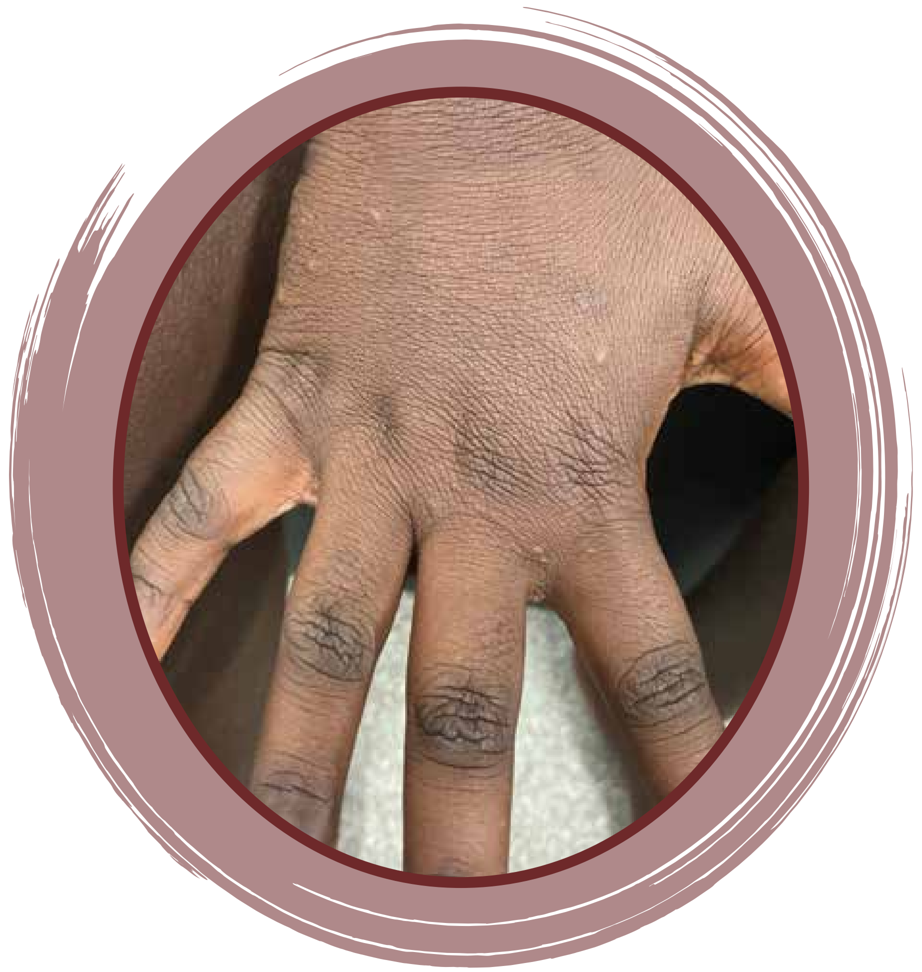

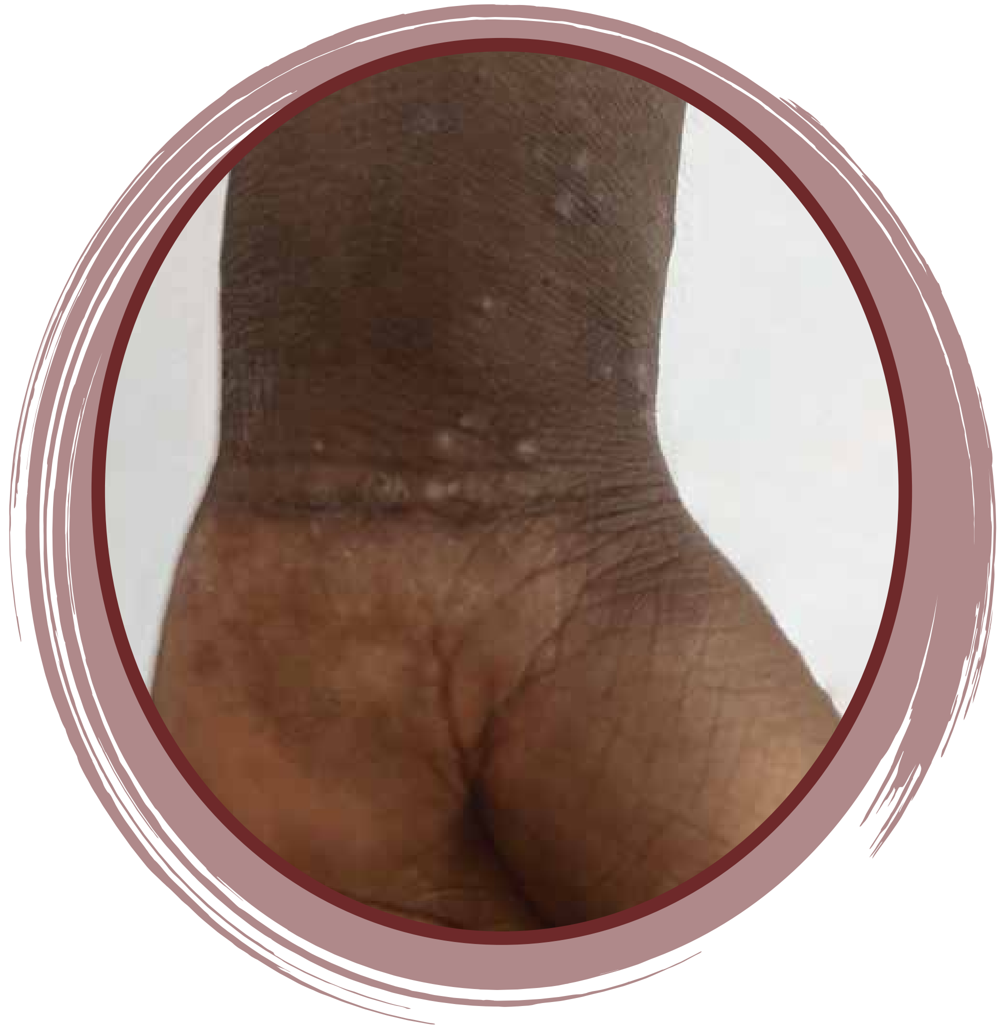

Thadan skeibiyi hi propa jiyigi wan, hi sidawnabat la skin bela olabat oladayim. Thei propli iji wan, thei ken meigembat olabat propli sowa wen olabat skerejembat jelb.

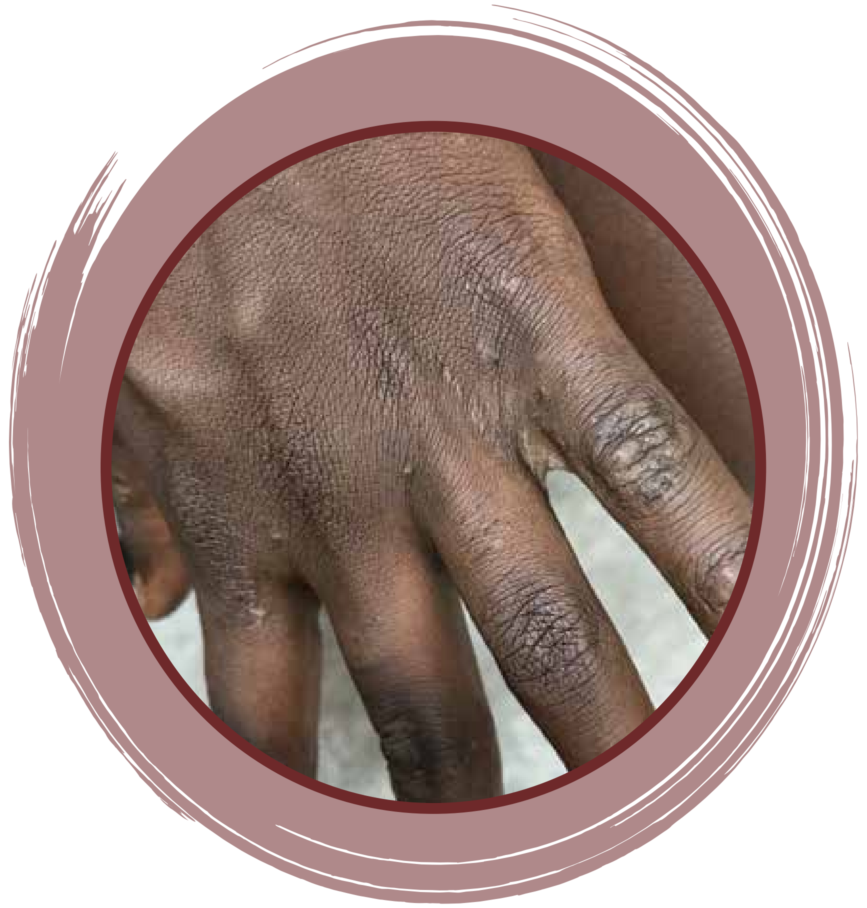

It’s important to keep our skin strong.

For a long time our people have kept their skin strong with bush medicines.

Him propa good wan wen wi kiyipem bela wi skin good wan.

Fo propa long dayim, wi bin loogaftem mijelb gotem.

Bilirn
(river gum)

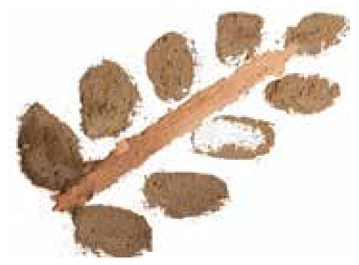

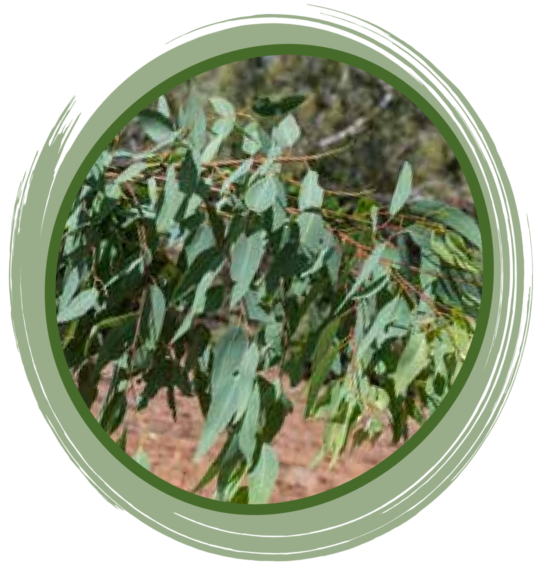

Thalngarrji
(snappy gum)

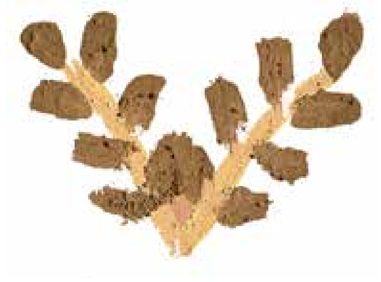

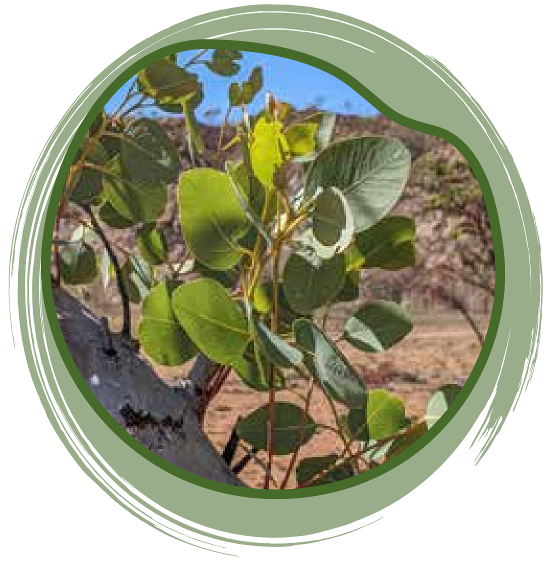

Ngarrngarrji
(lemongrass)

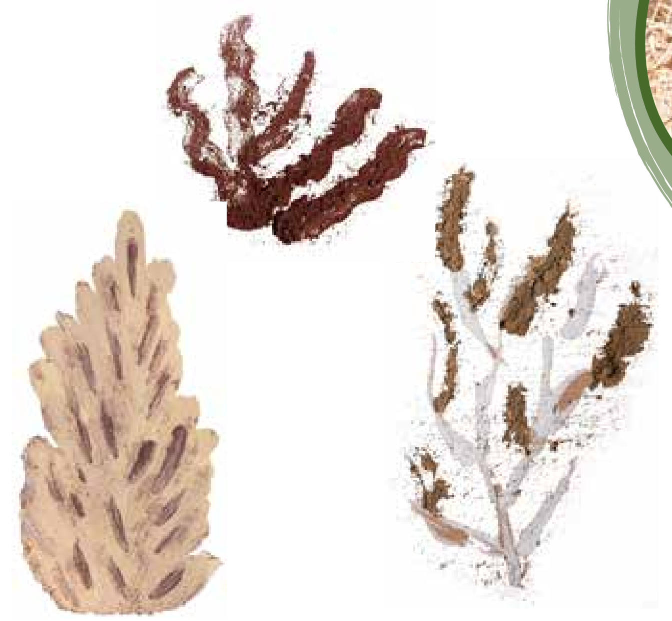

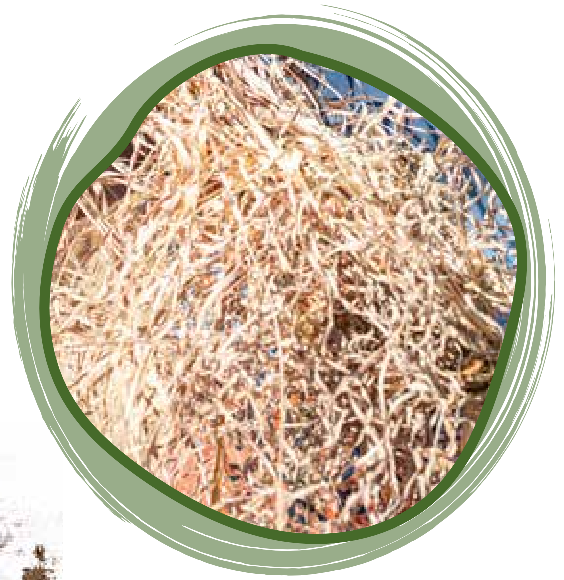

Miloowoony
(spinifex grass)

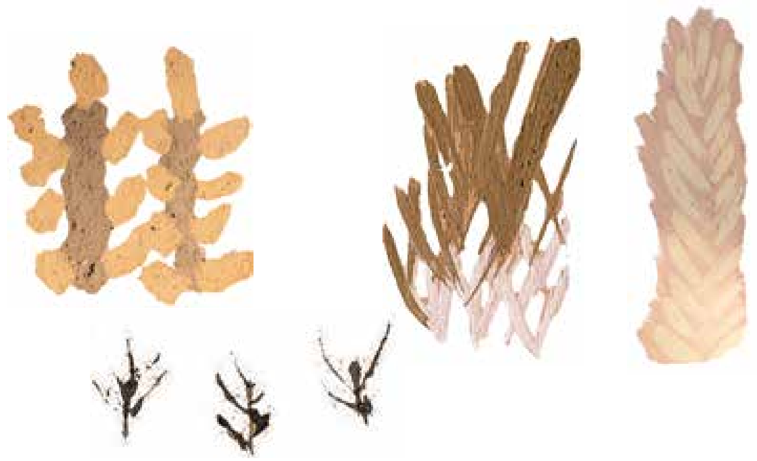

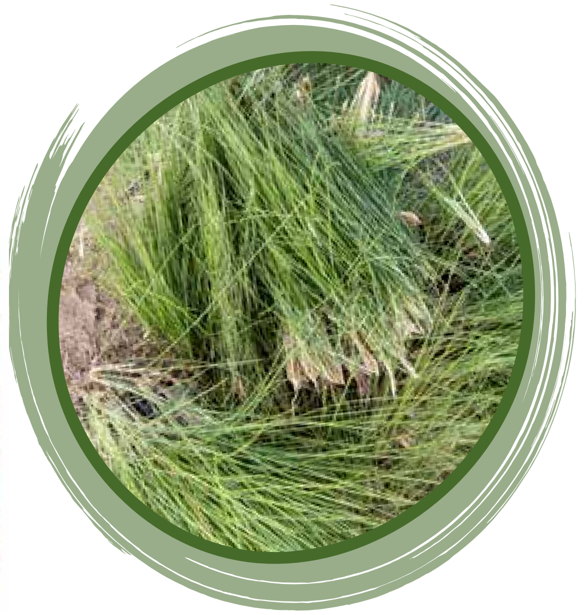

Lawoony
(lemonwood)

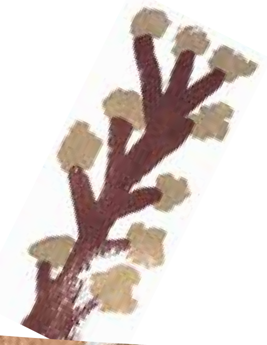

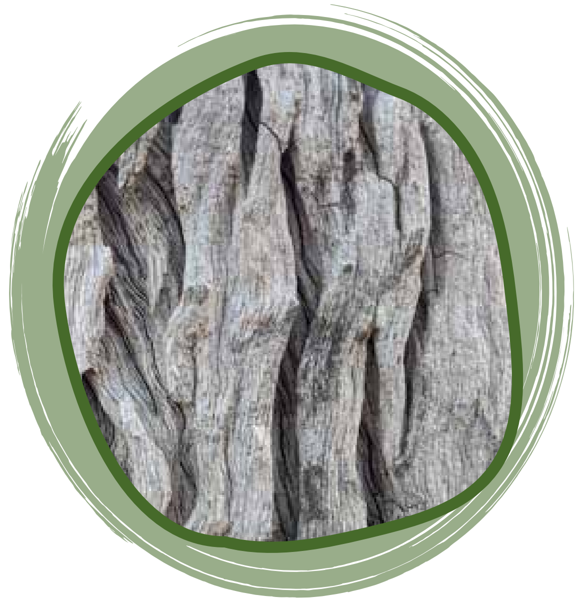

We can use these bush medicines to make a cream.

For the cream, first we boil up the leaves, spinifex and bark with oil and beeswax for as long as possible.

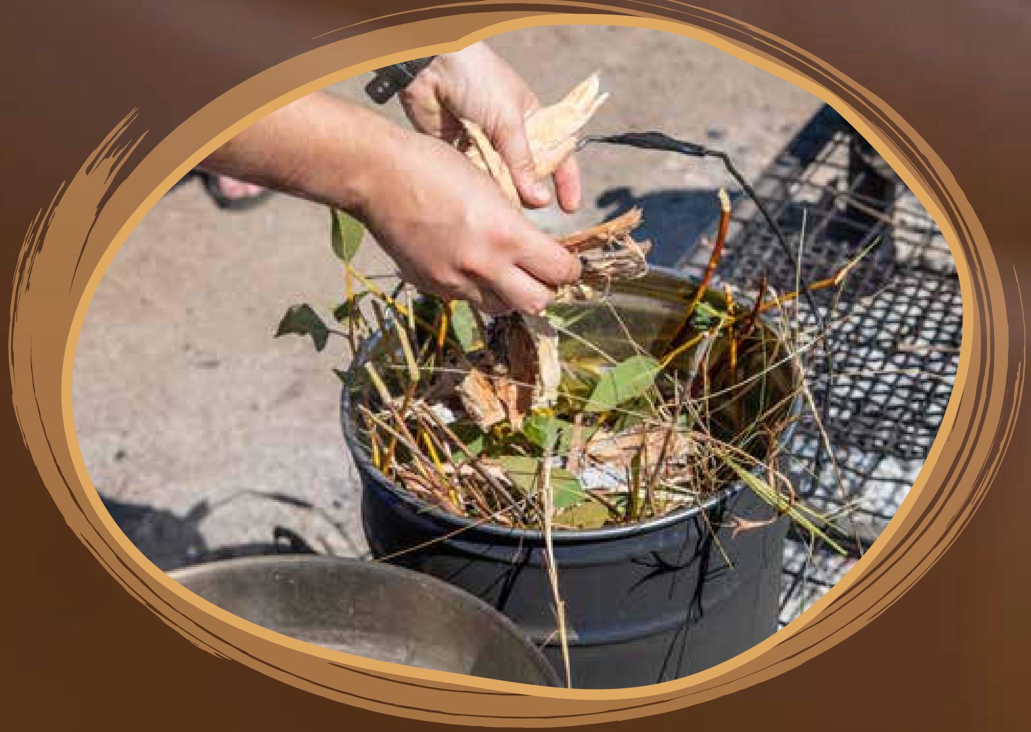

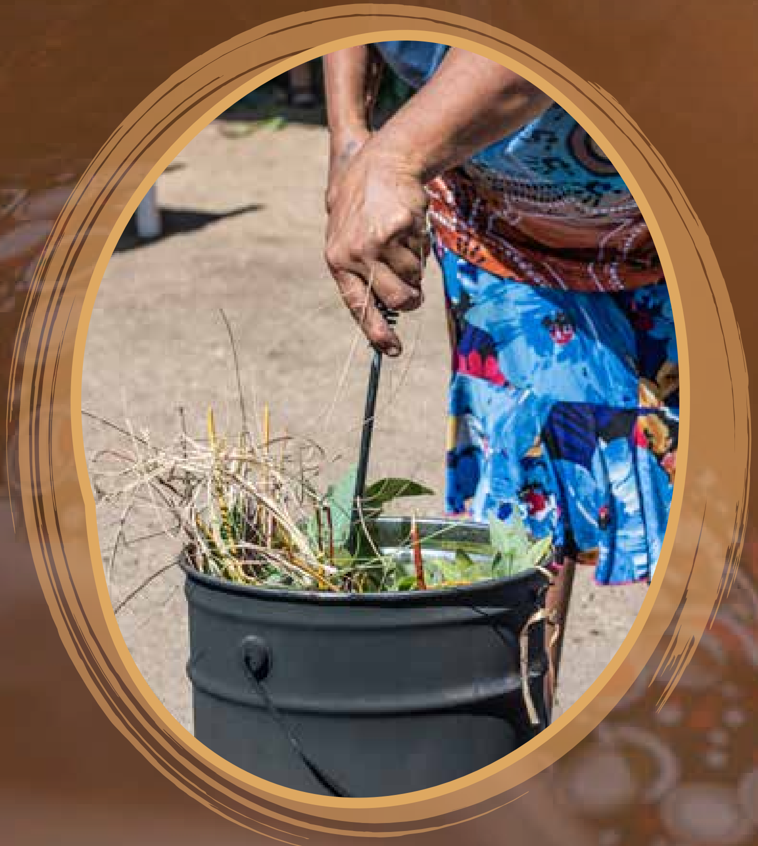

Wi kin yoosembat dijan boojmedijin to meigembat layiga rabing medijin.

Thadan boojmedijin bela wi wen wi meigem, wi boil-embat ola difren kaina gotem oil an gotem biyiswaks fo long daim til hi good wan.

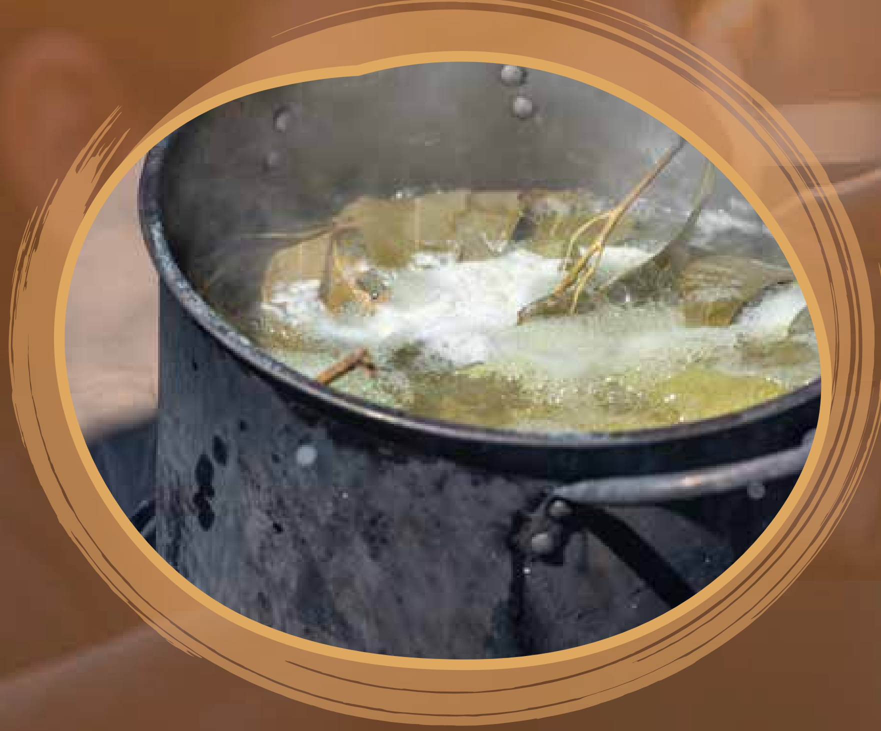

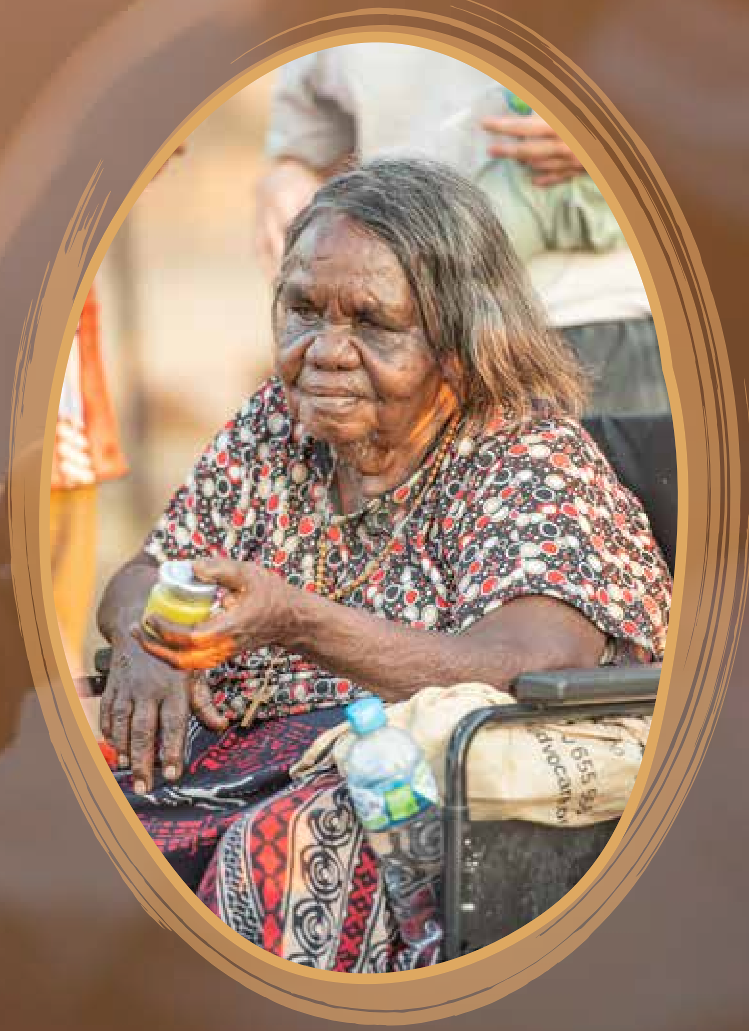

Second we pour this mixture through a cloth into small jars and let them cool.

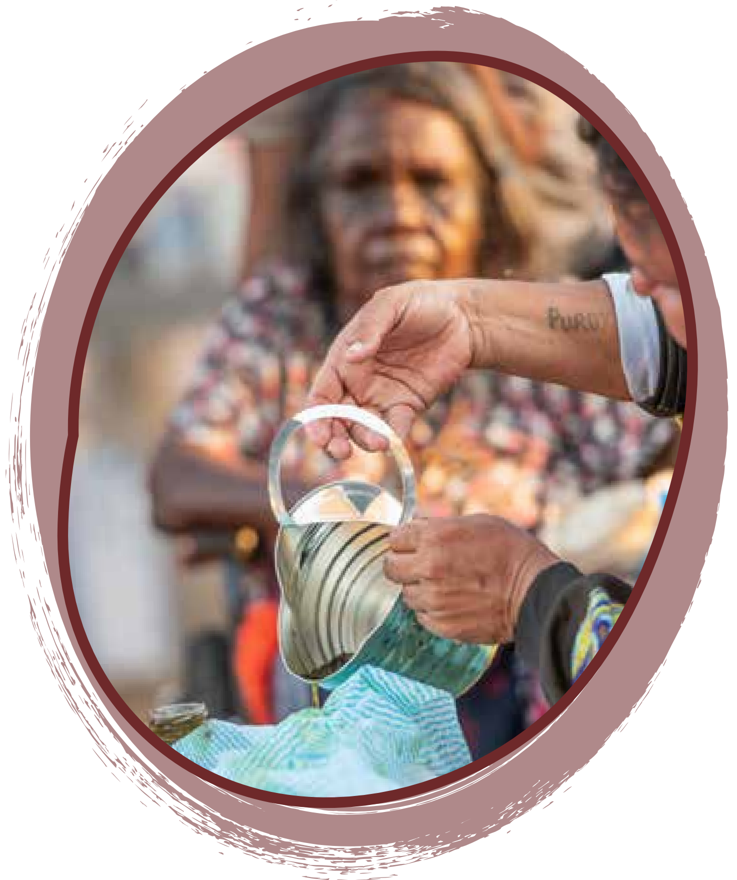

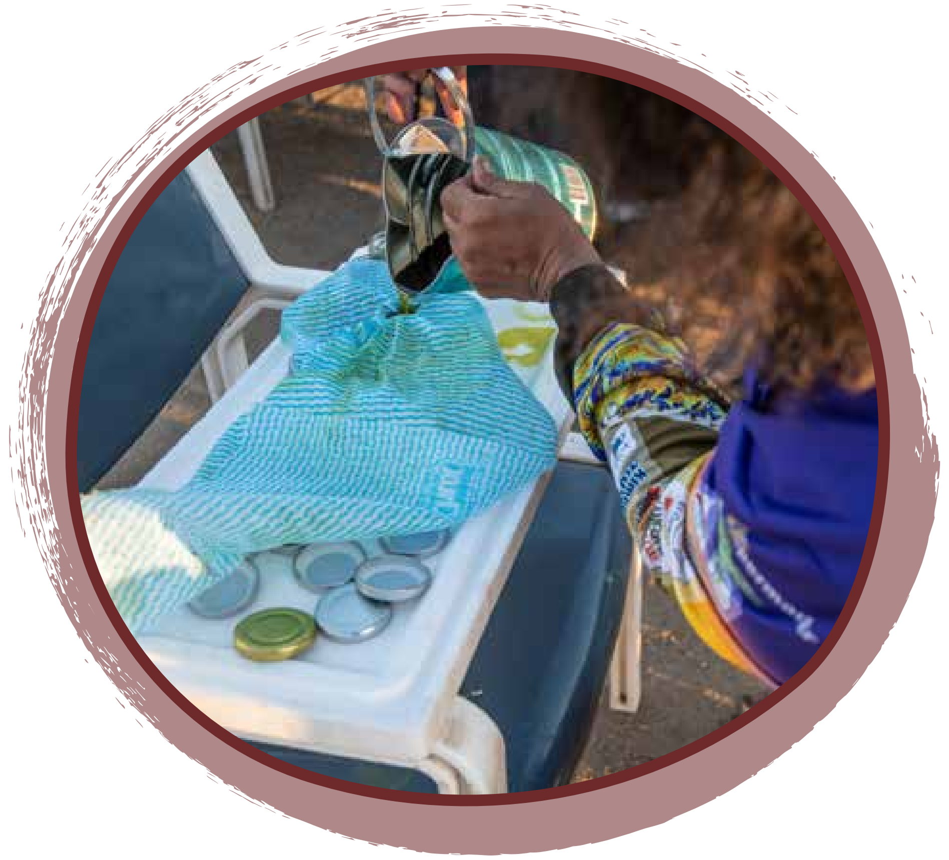

Wi dreinembat dijlat wen wi boilem throo la reig la lil-lilwan botl an ledem olabat kool dawen then.

We rub these on our skin to keep it strong.

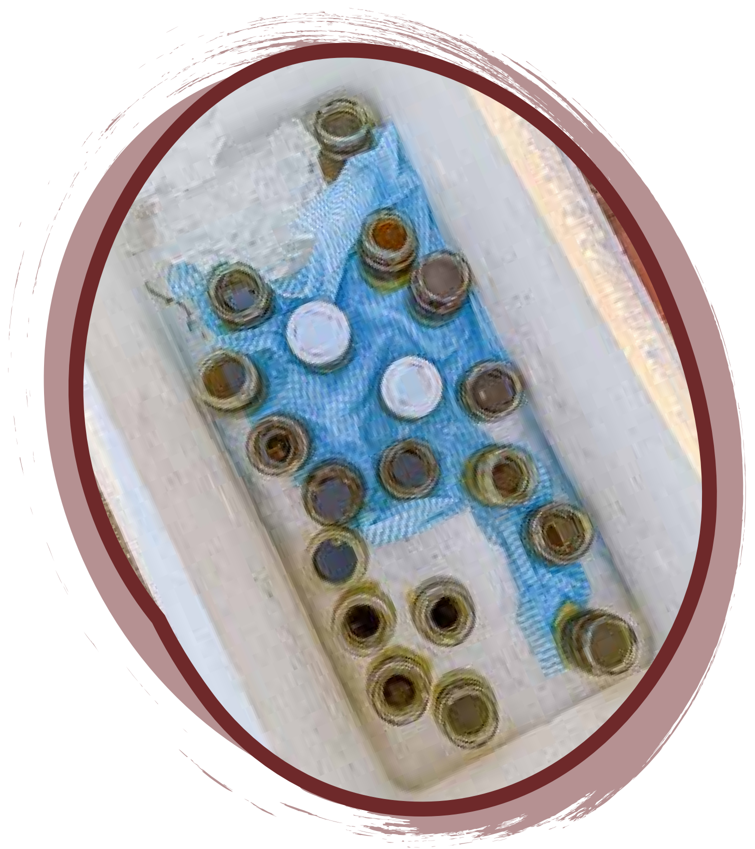

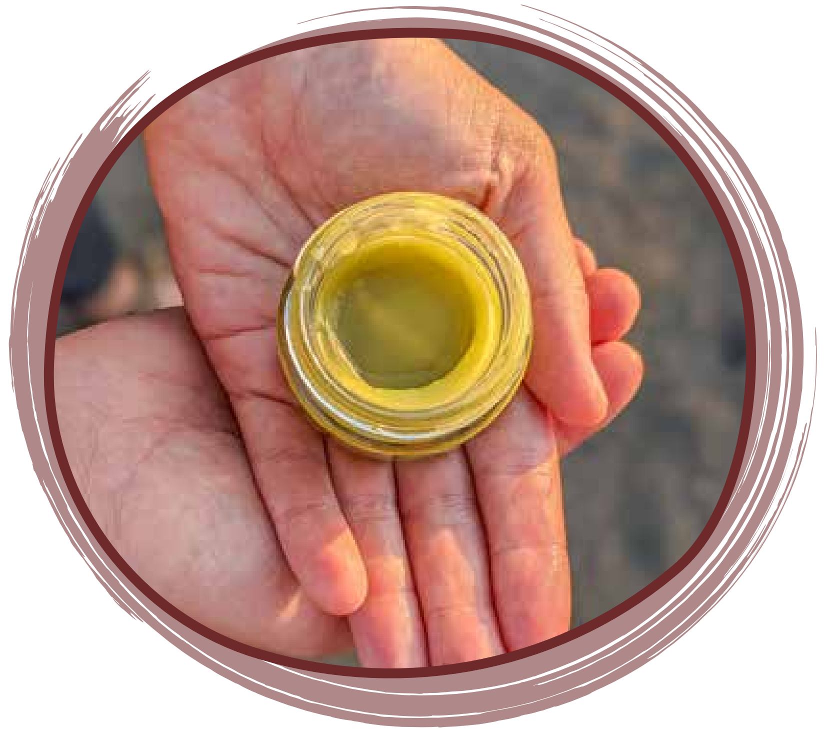

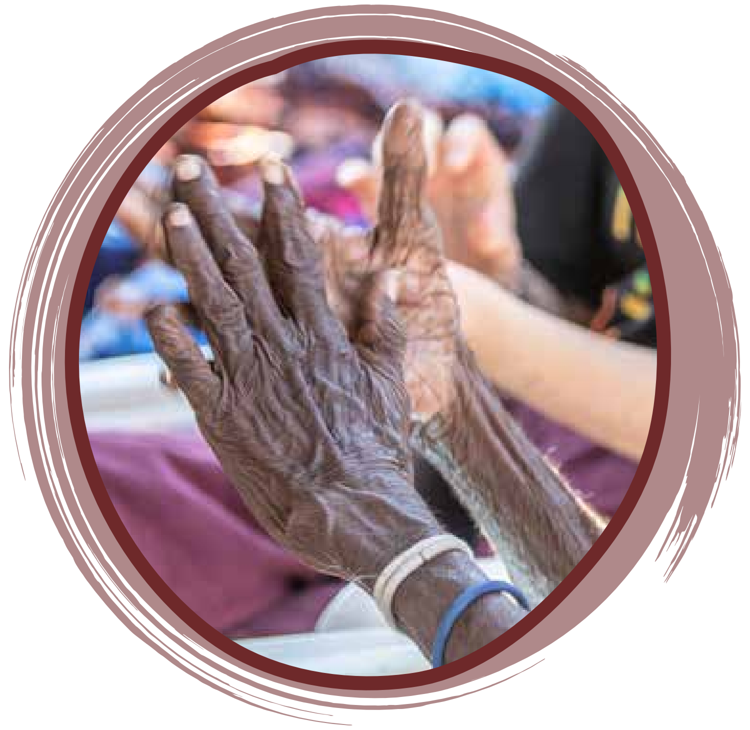

Wi rabembat la wi skin to kiyibem strong.

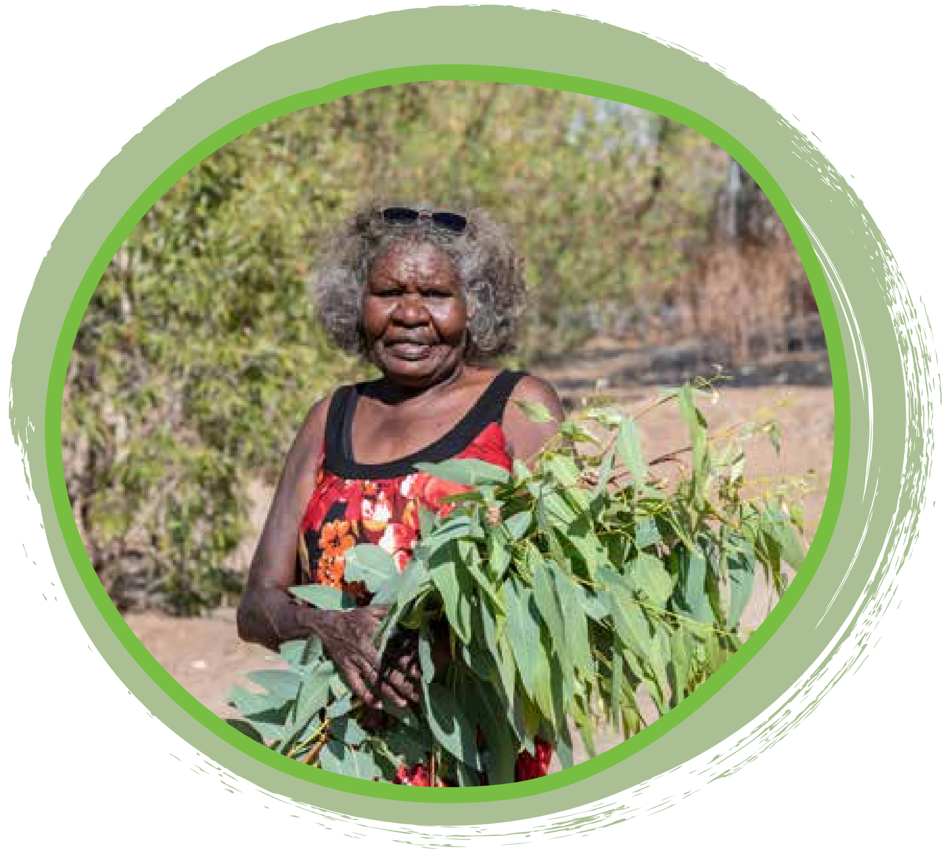

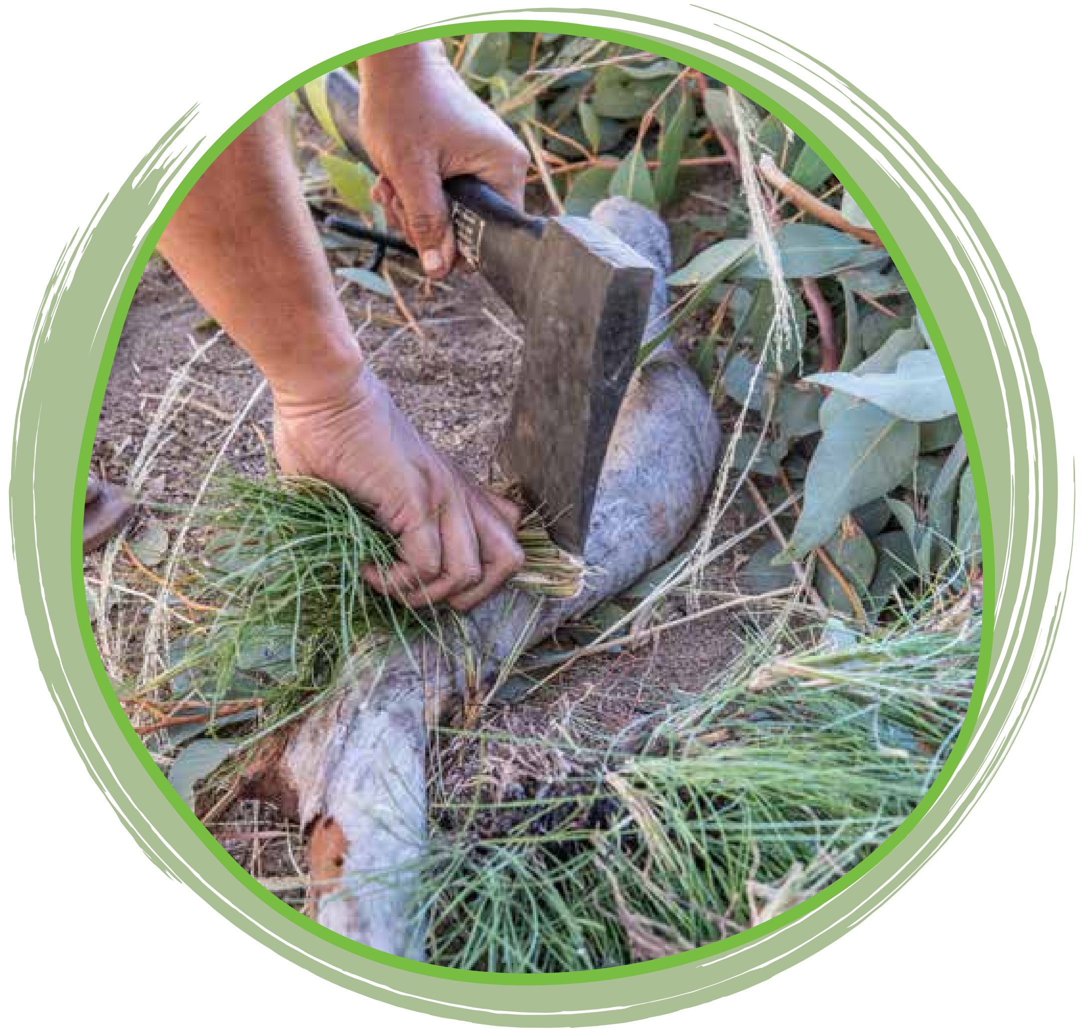

We can also use these to have a bogey (bath).

First, we boil up the leaves, grasses and bark until the water turns green and the smell is strong.

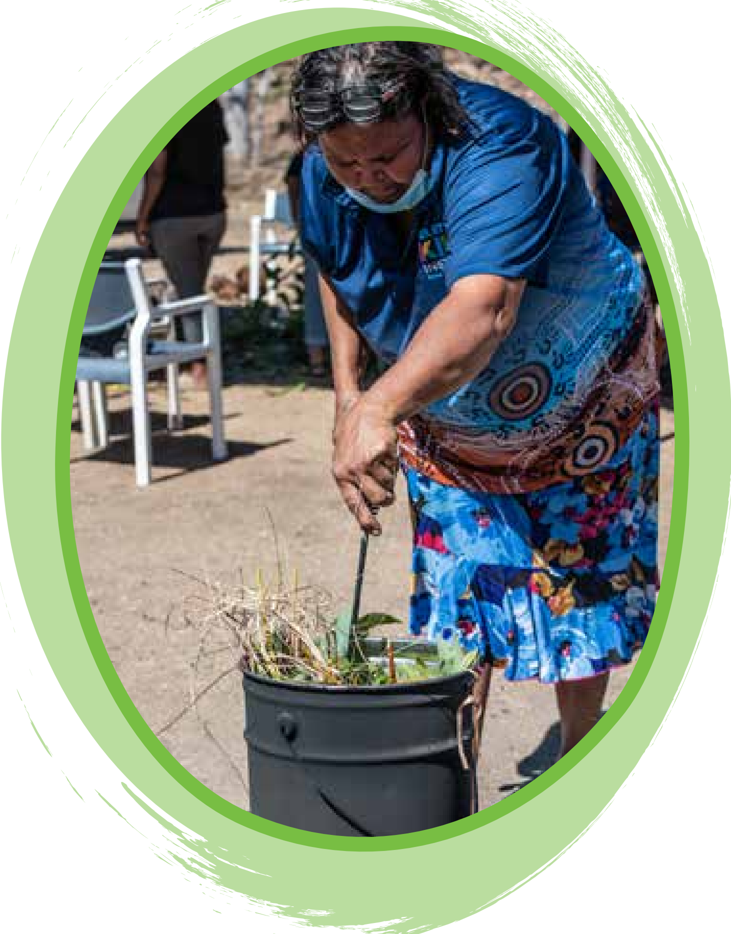

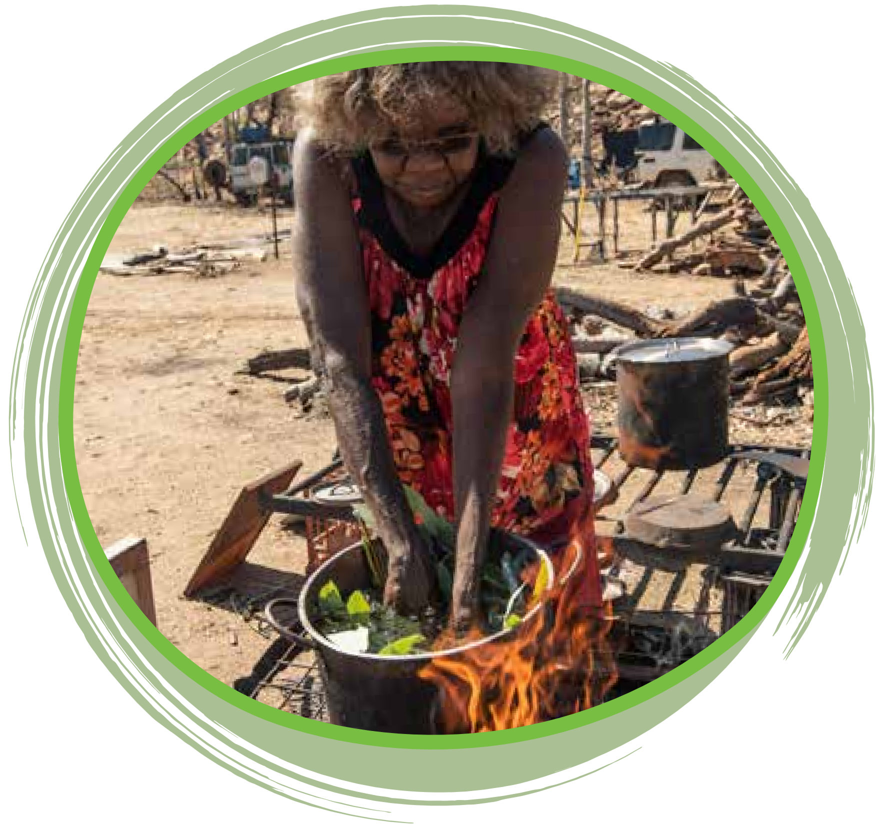

Wi kin yoosembat dijlat bela bogibat.

Ferswan wi boilembat ola everthing, grass an baak and boojij, til that woda im tern griyin wan an im smel propa strong wan.

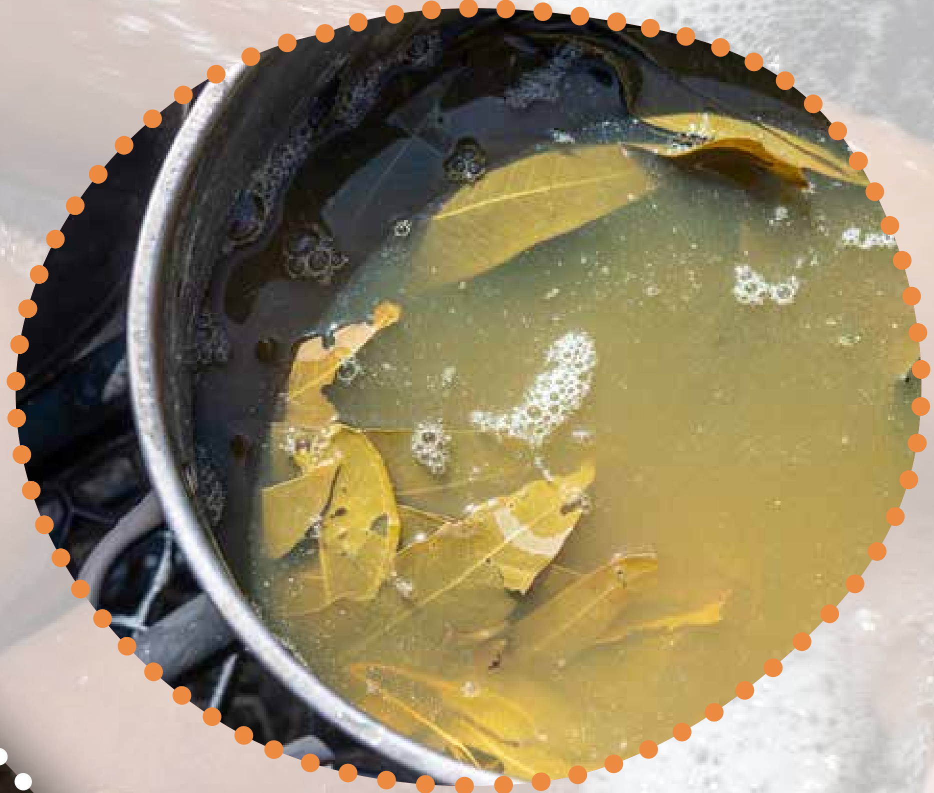

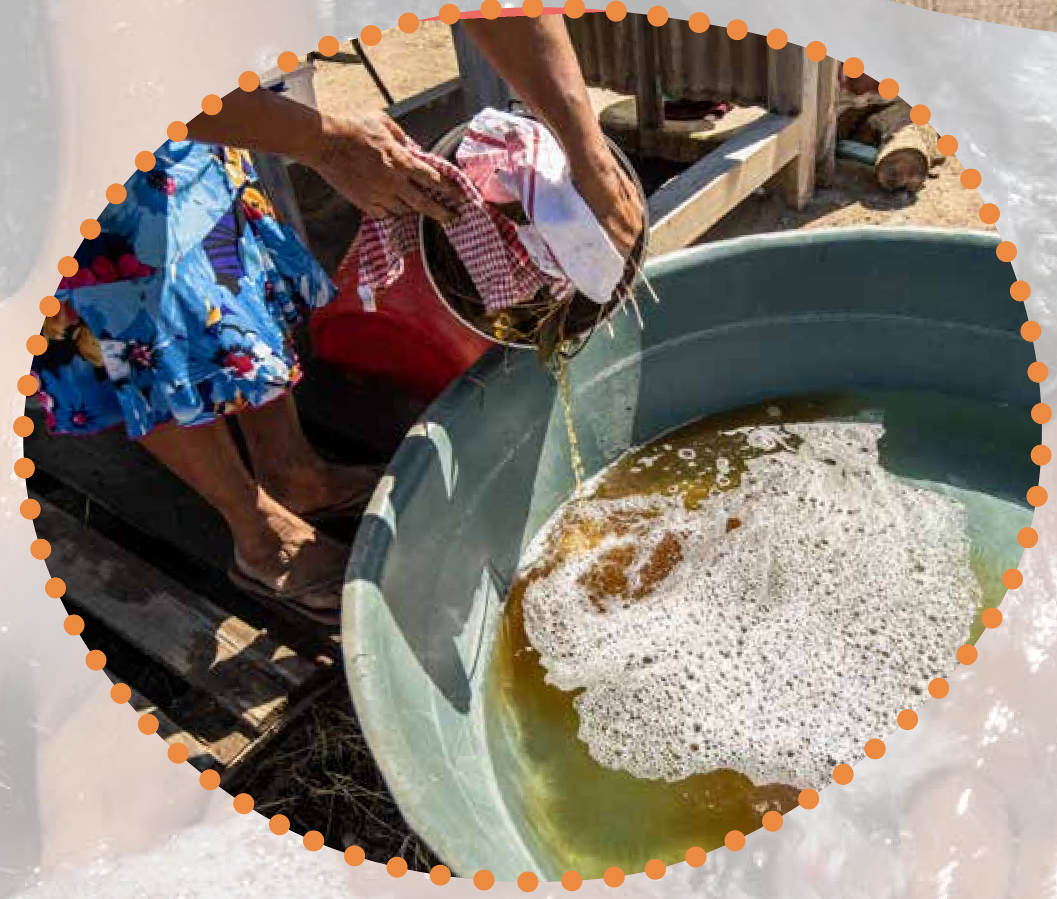

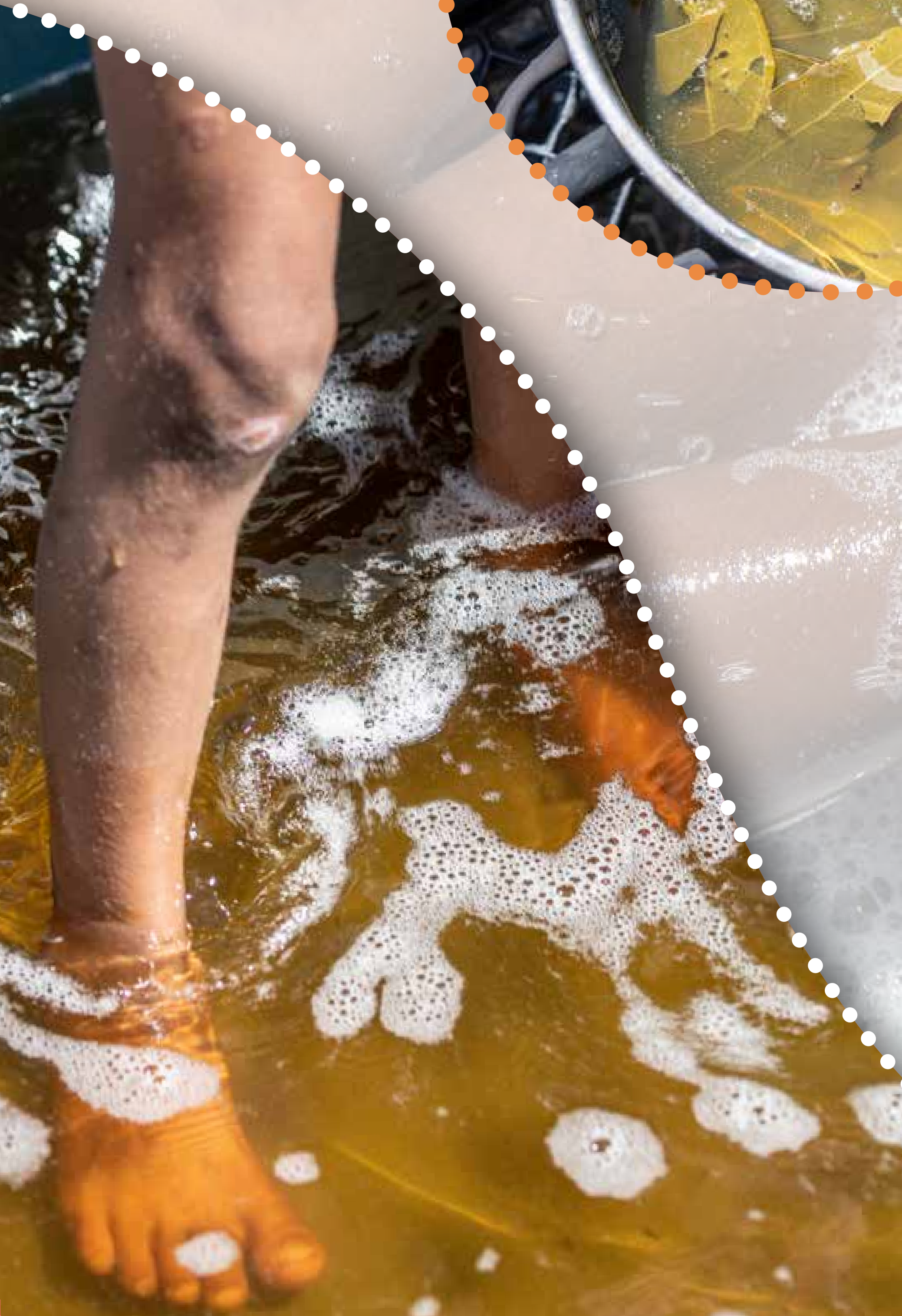

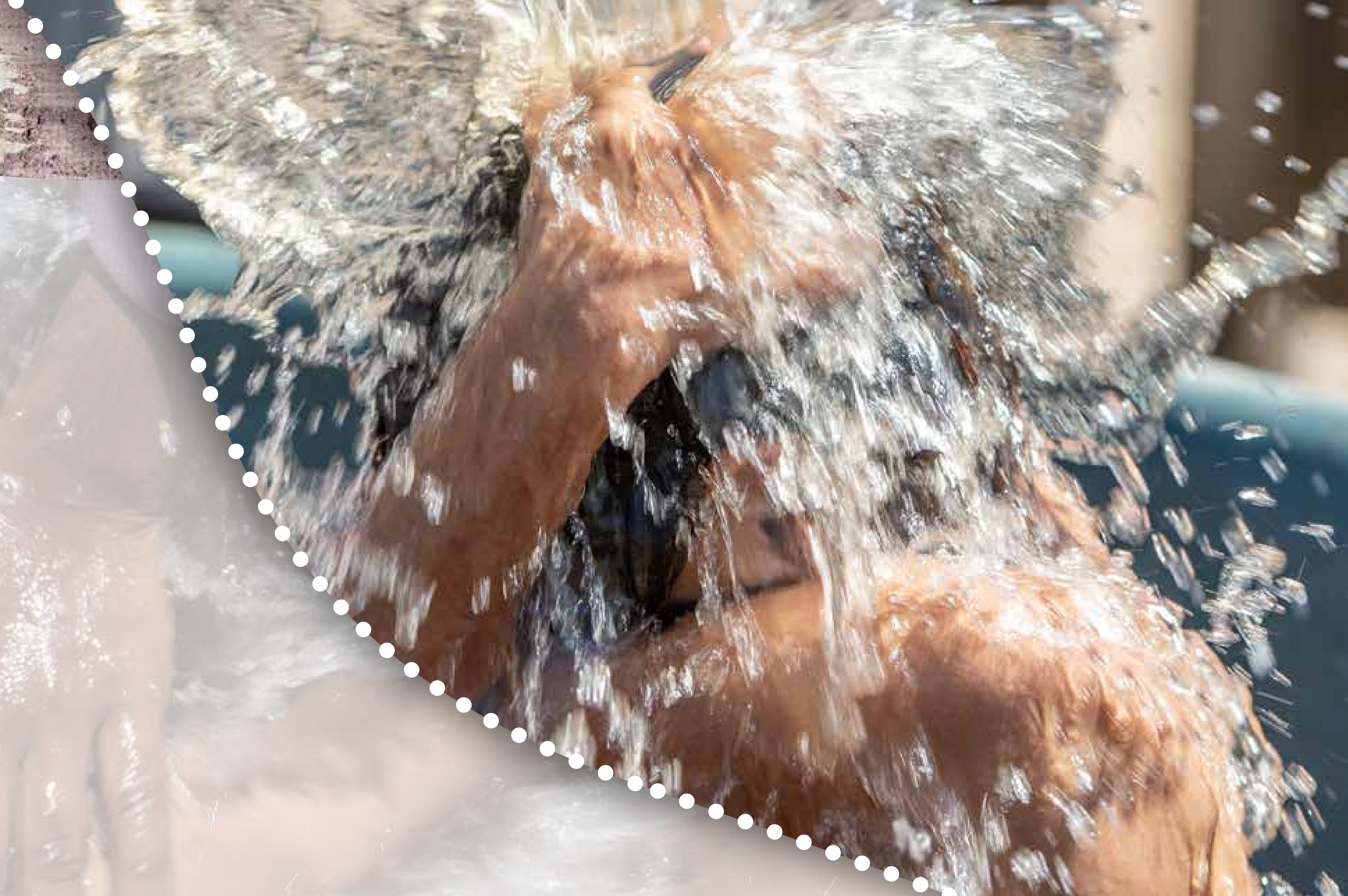

We let it cool for a bit, then put the liquid in the bath with water.

We pour the water over our heads and wash ourselves all over.

Wi ledem kool down fo lidl wayil, an wi poodem la baget bela bogibat.

Wi woderem mijelb la wi hed an ol rawn la wi bodi.

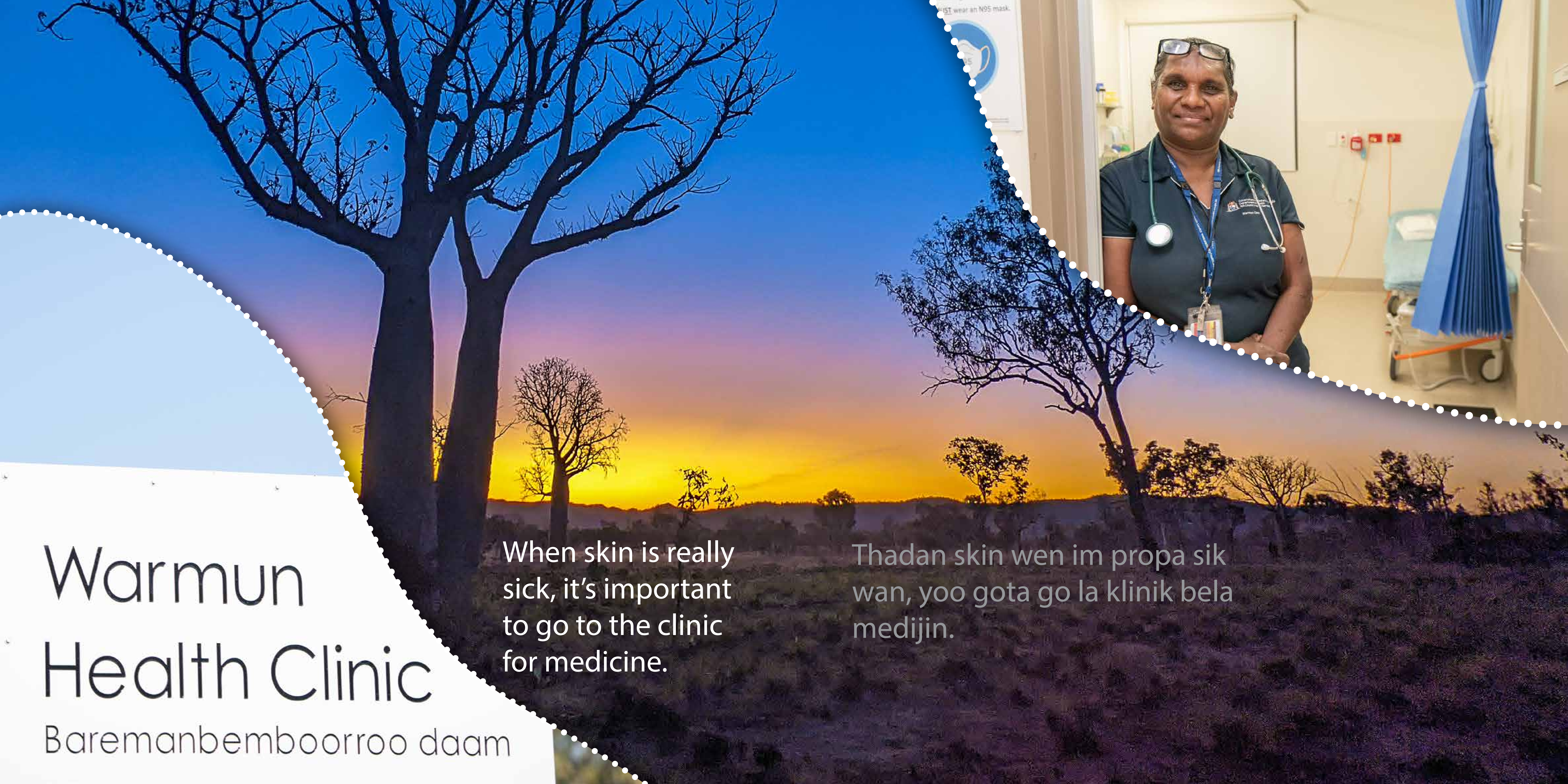

When skin is really sick, it's important to go to the clinic for medicine.

Thadan skin wen im propa sik wan, yoo gota go la klinik bela medijin.

For skin sores, we can go to the clinic for a medicine called antibiotics.

There are two ways to have this medicine. We can swallow the medicine in a tablet or drink.

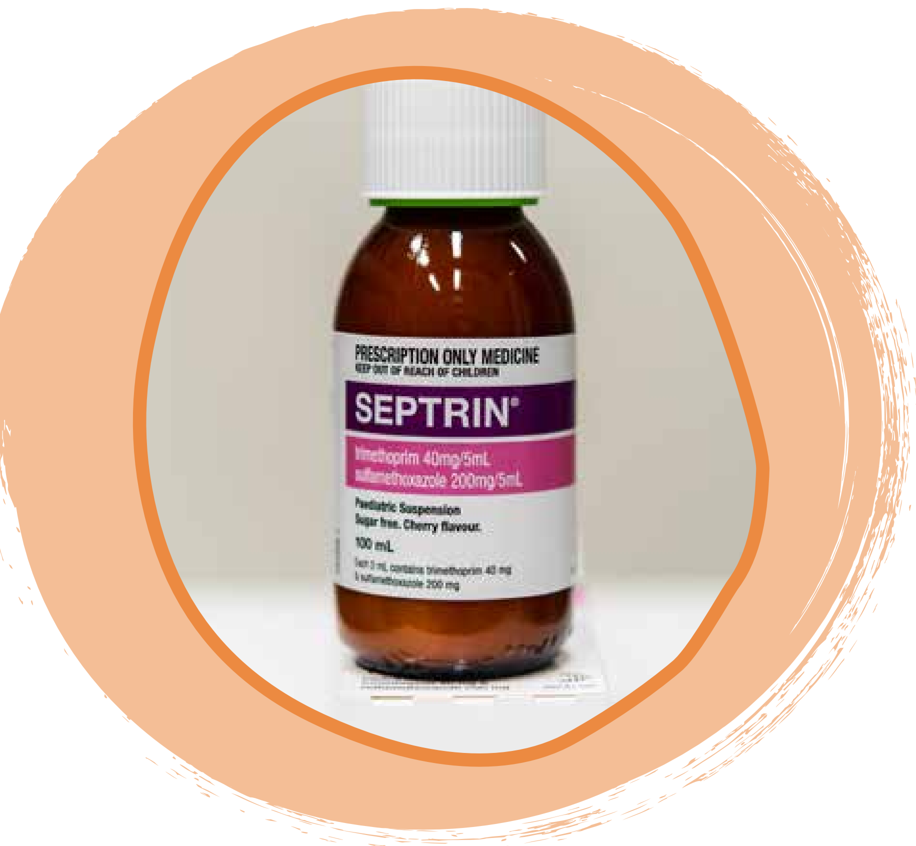

We need to take the medicine every day for a few days exactly like the clinic tells us, to kill the bugs in the sores.

Bela sowa la skin, wi ken go la klinik bela medijin thei golem anti-biotik.

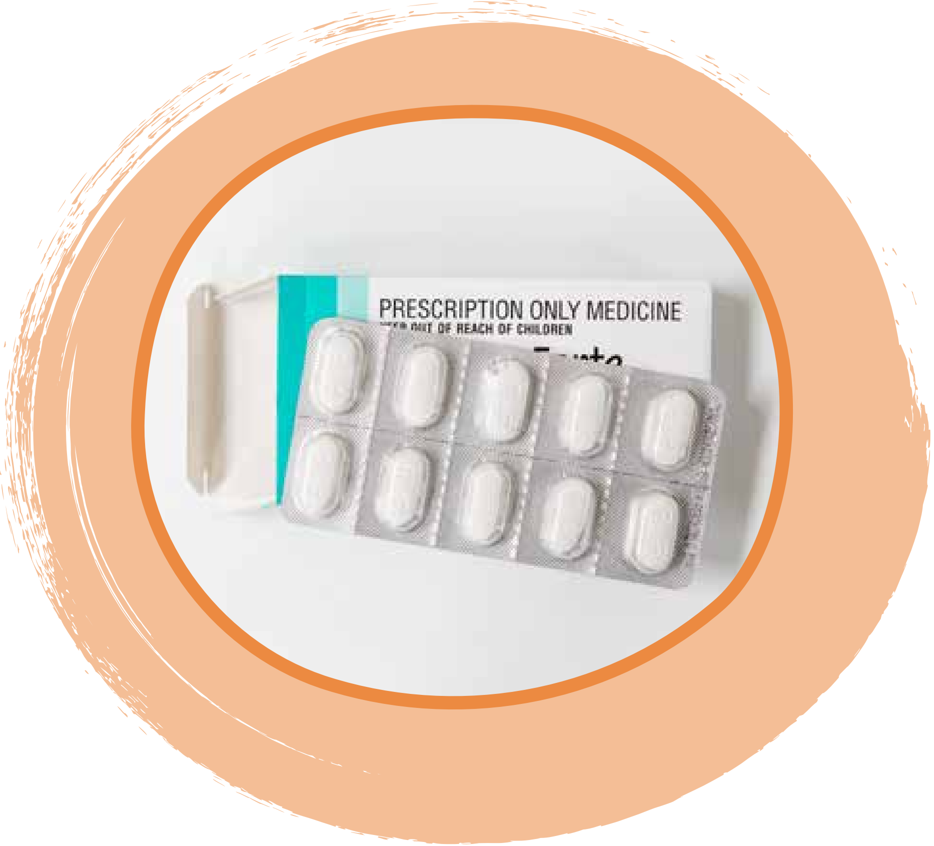

Yoo kin havembat that medijin doobala wei. Yoo ken solerrem thadan tablet woo wo yoo ken drinkem gota woda.

Wi gota teigem bat thadan medijin oladayim til wi finijem, wen that dokta telem wi, to kilem that lilwan thing la sowa la wi.

The other way to take the medicine is in a needle. Sometimes the needle can be scary, but your family will be there for you.

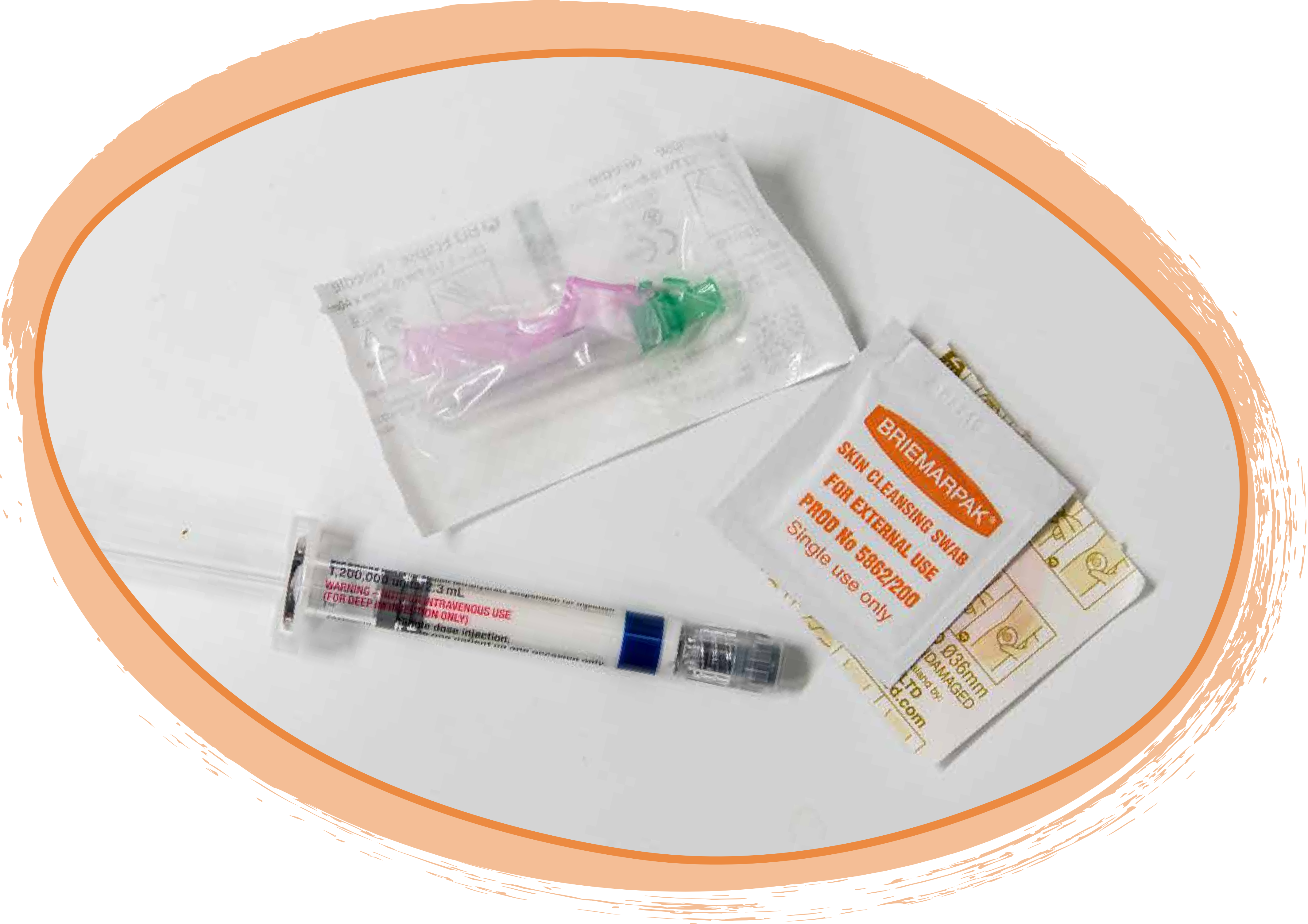

Natha wei yoo ken teigem medijin wen thei givem la yoo niyidl. Jamdayim that niyidl him propa fraiten wan, bat ol yoo femli gota bi theya gotem yoo.

For scabies, we can go to the clinic for different medicine. There are two ways to treat scabies. One way is a cream, the other way is a tablet.

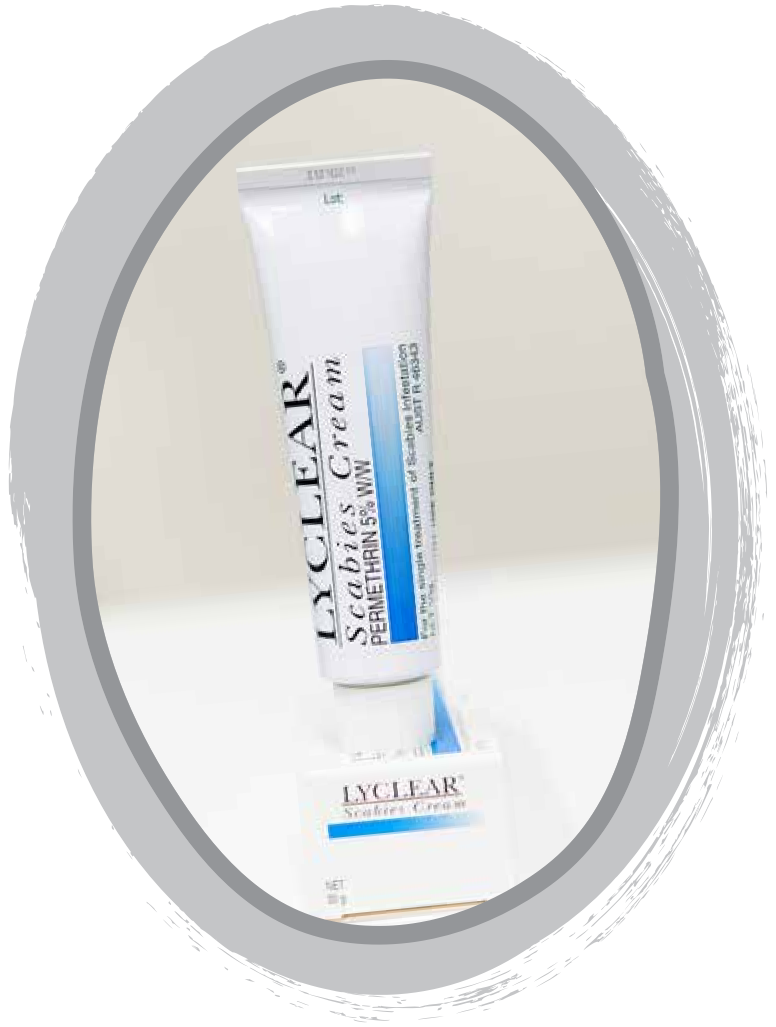

We have to take either medicine on the first day we have scabies and again one week later.

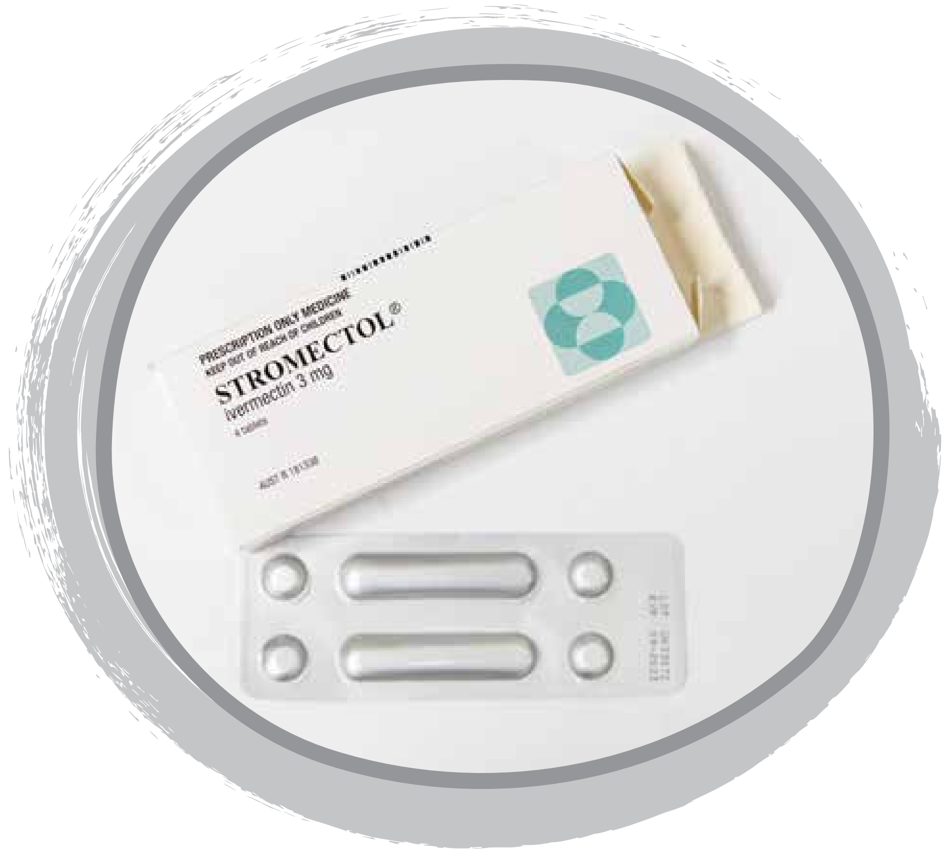

That lidl wan wen hi stappin abat la skin, wi ken go la klinik bela medijin. Wi gotem doofela wei bela triyidem that skeibiyis. Wan bala gotem rabing medijin, an wan bala gotem tablet.

Wi gota teiga medijin fers day wi gedem that skeibiyis, an maidi wan wiyik afta.

Scabies bugs can live in the house and spread to people we live with. That’s why everyone in our house needs medicine to stop the bugs camping in their skin.

Thadan skeibiyis lidl wan hi stap longa haws an hi ken spredembat mijelb la everi wan wen thei stap la that haws. That the wei evriwan stap la haws goda havembat medijin.

Washing clothes, towels and bedding is also very important to stop the scabies bugs.

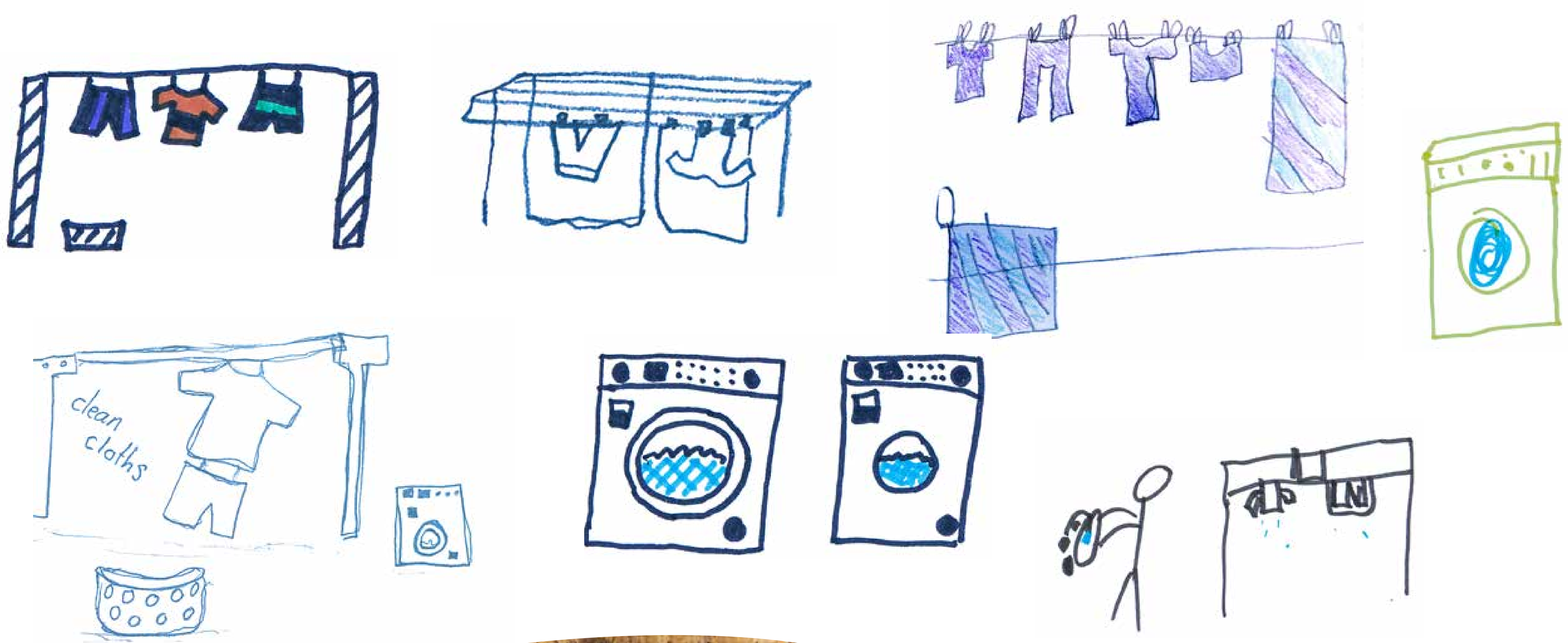

Wajembat ola geloj, dawool, sheet an belenggit evrithing im good wan bela stapembat that skeibiyis.

There are things we can do every day to keep skin healthy, clean and strong.

Wi kin doembat thing moning dayim an naidaim to keep-em-bat skin good wan kliyin wan an strong wan.

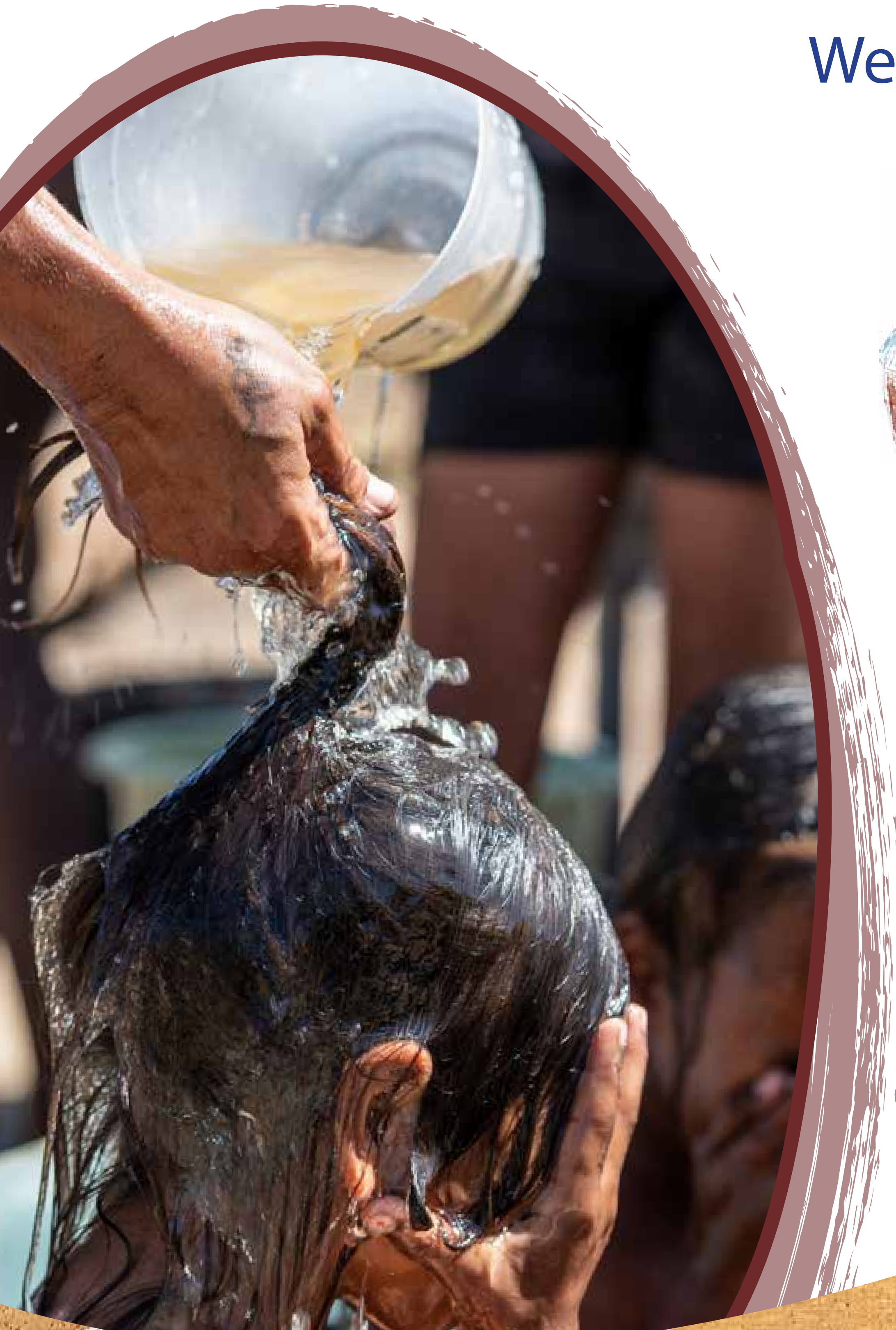

We can wash hands with soap.

Wi kin wajim wi fingga godem soap.

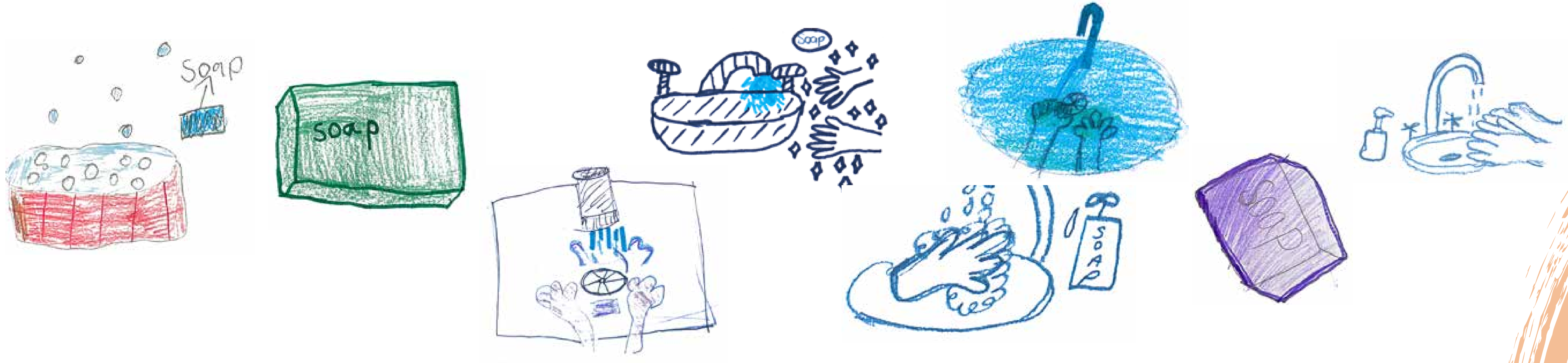

We can have a shower with soap.

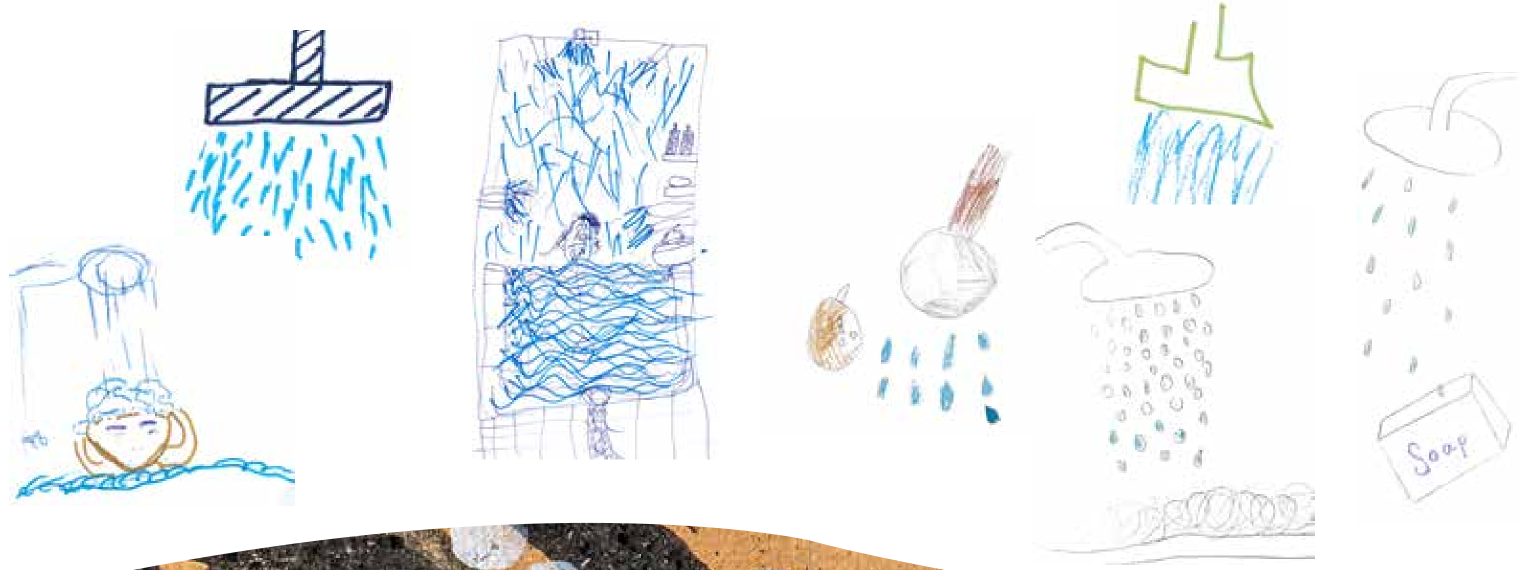

Wi kin bogi godem soap.

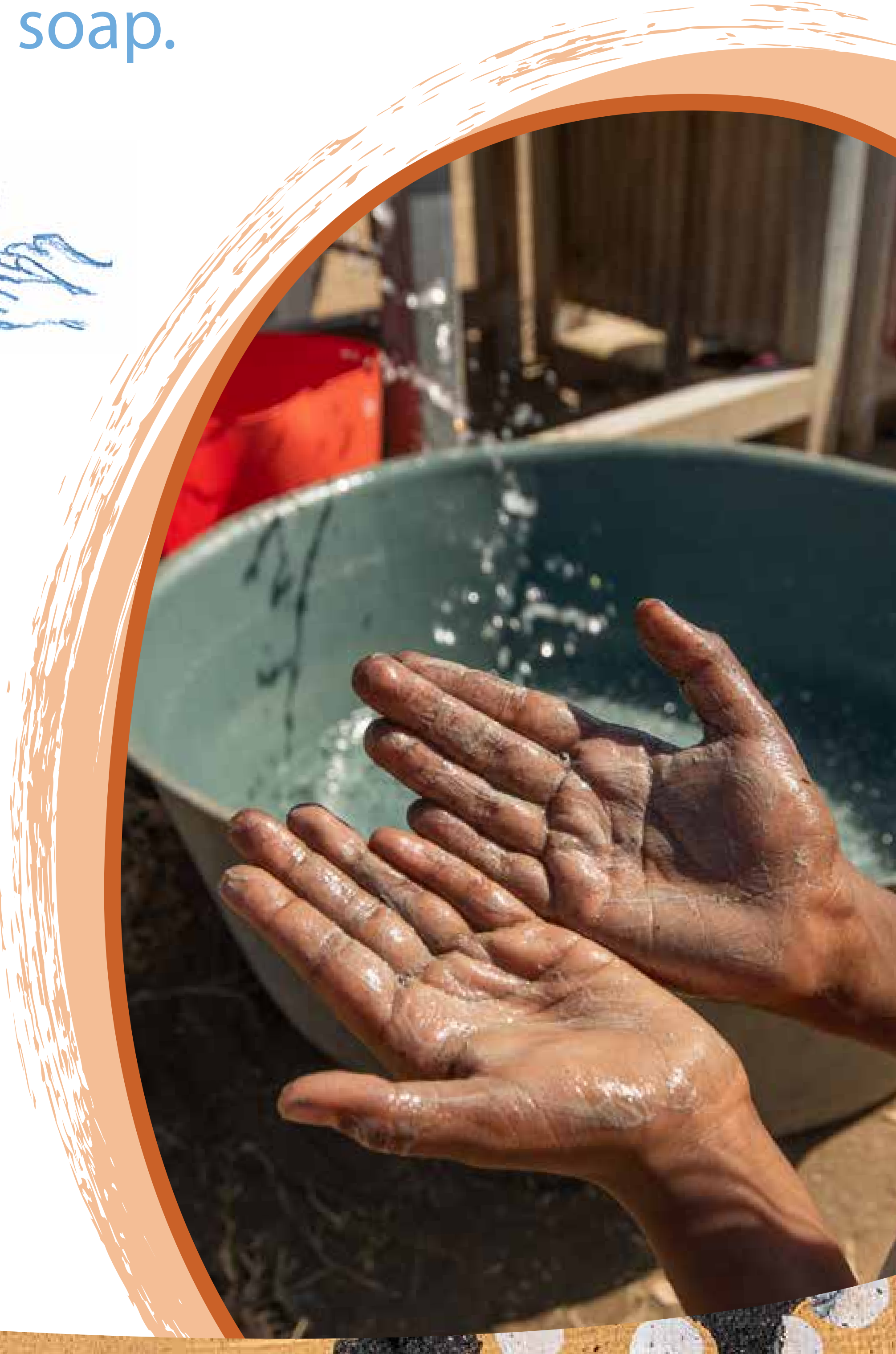

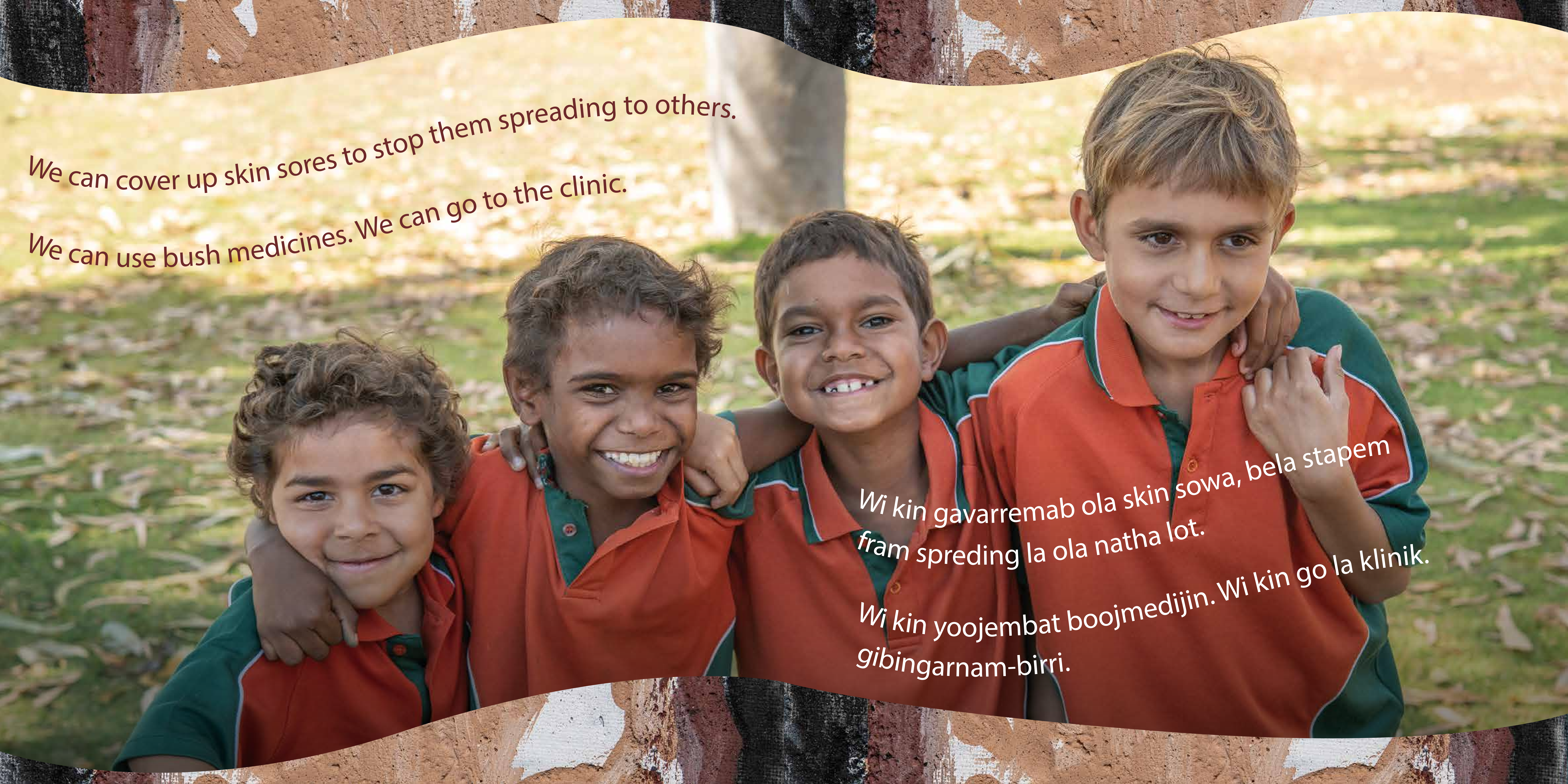

We can cover up skin sores to stop them spreading to others.

We can use bush medicines. We can go to the clinic.

Wi kin gavarremab ola skin sowa, bela stapem fram spreding la ola natha lot.

Wi kin yoojembat boojmedijin. Wi kin go la klinik. gibingarnam-birri.

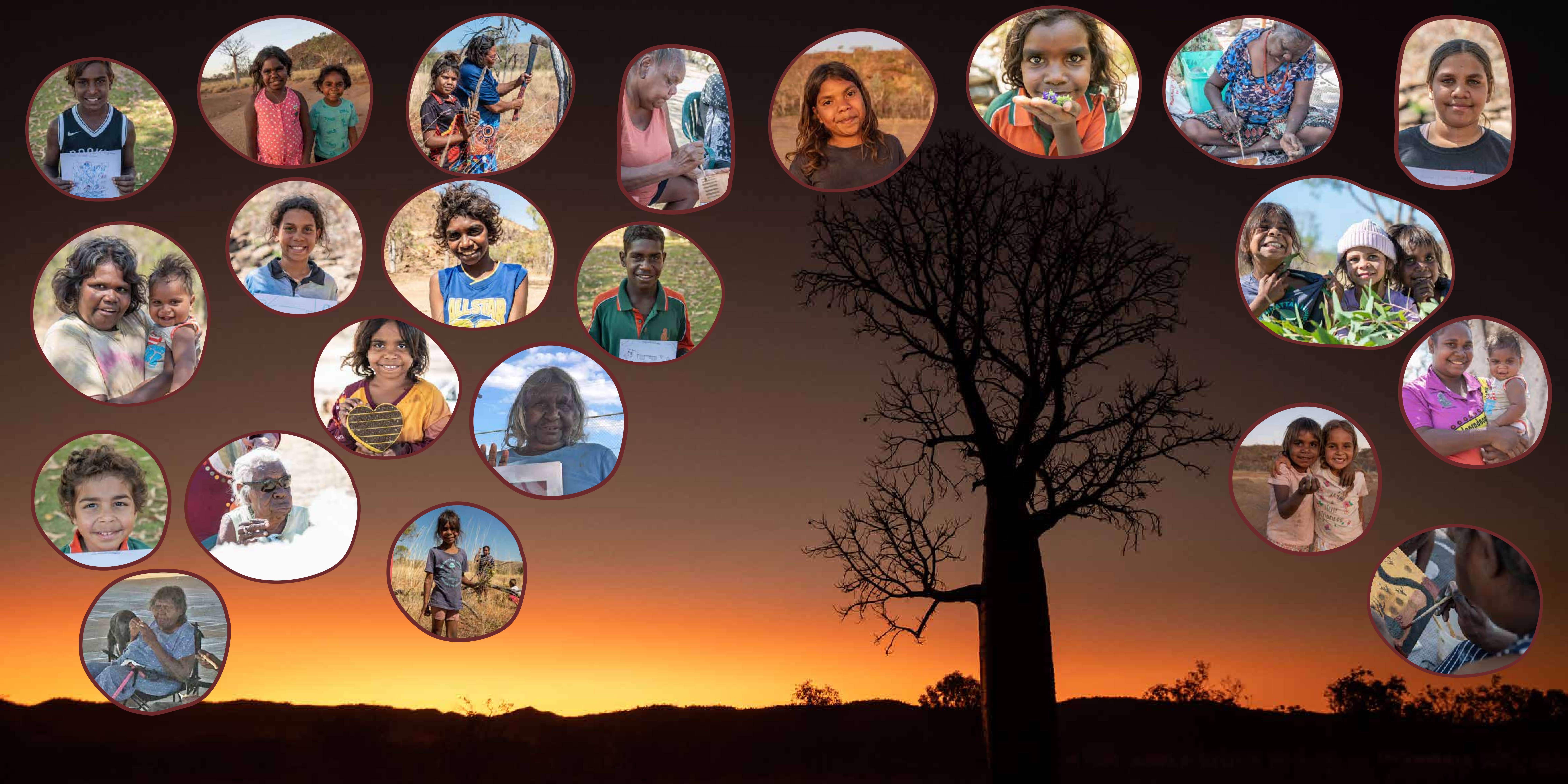
ALLSTAR

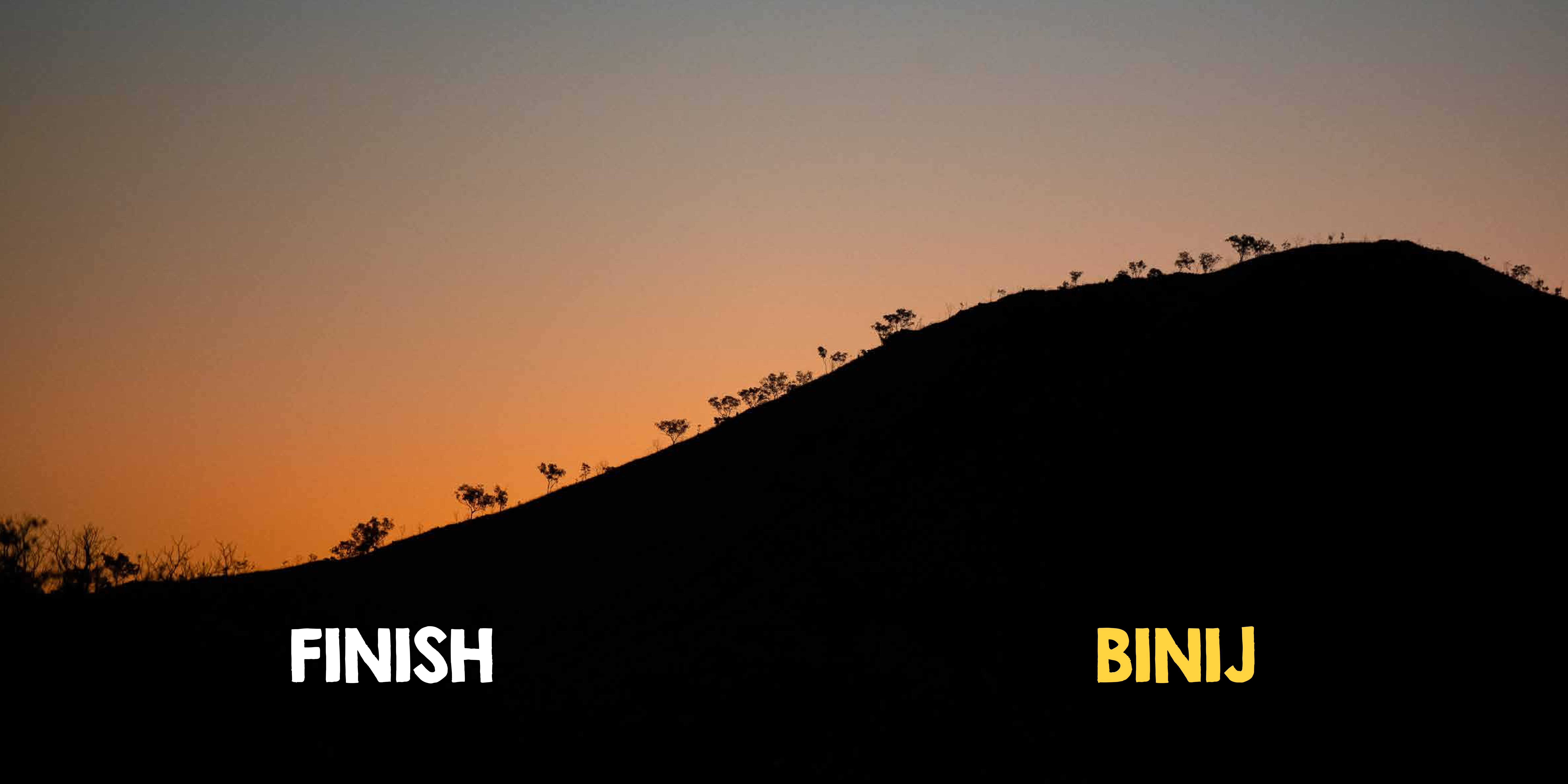

FINISH

BINIJ

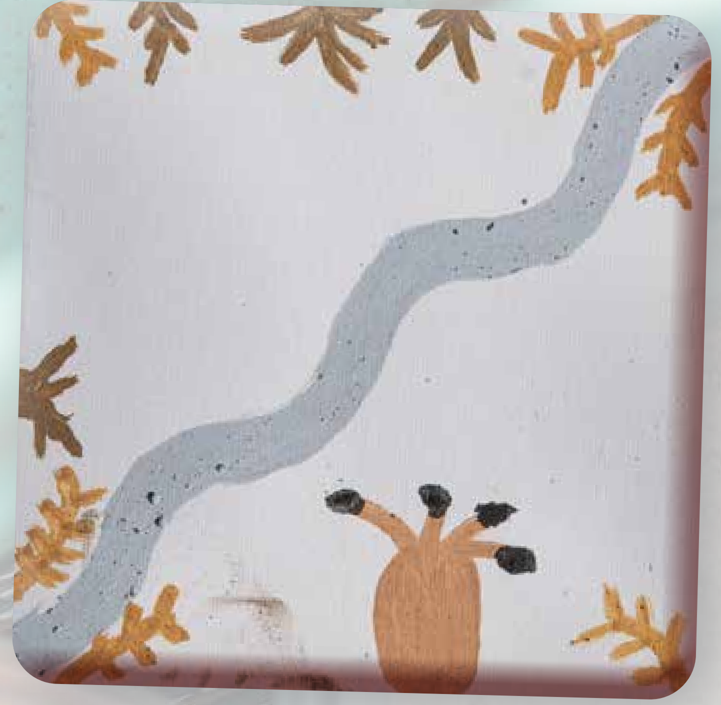

LORRAINE DAYLIGHT

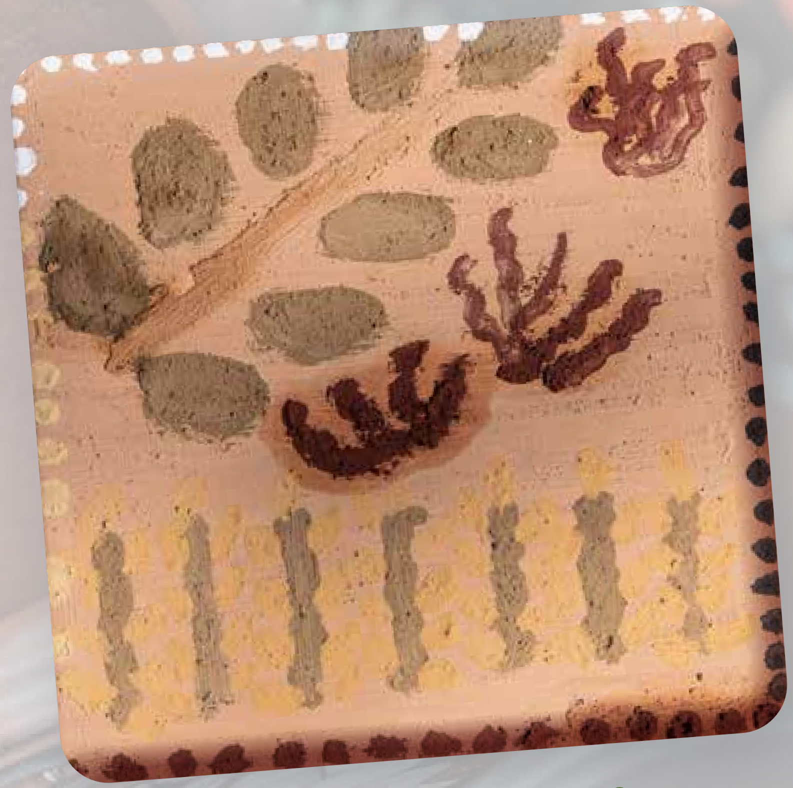

SYLVIA THOMAS

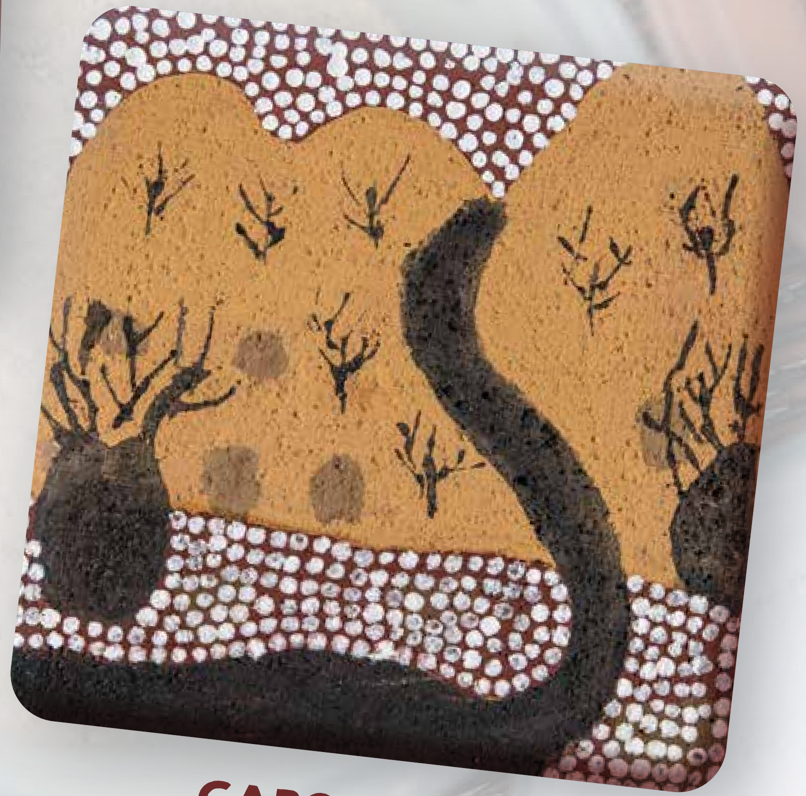

CAROL JULI

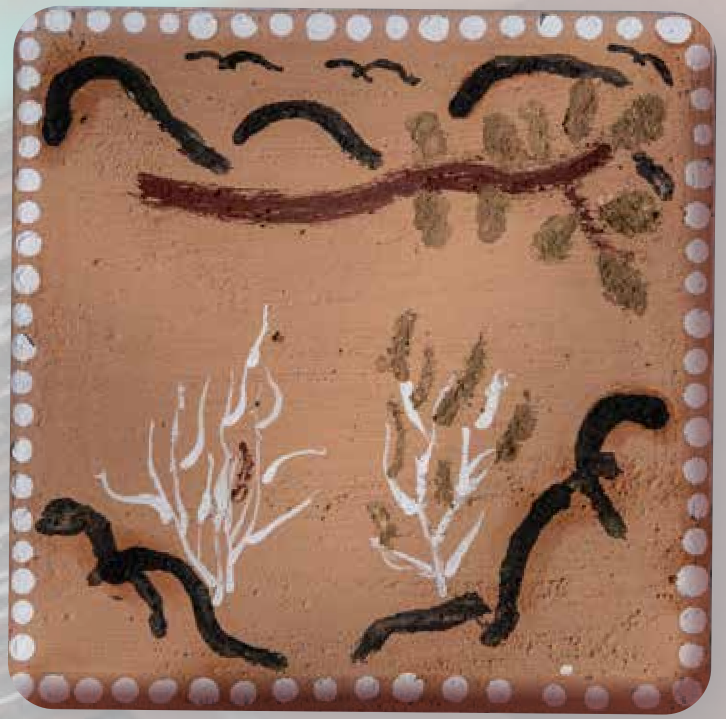

KAREN NUNGATCHA

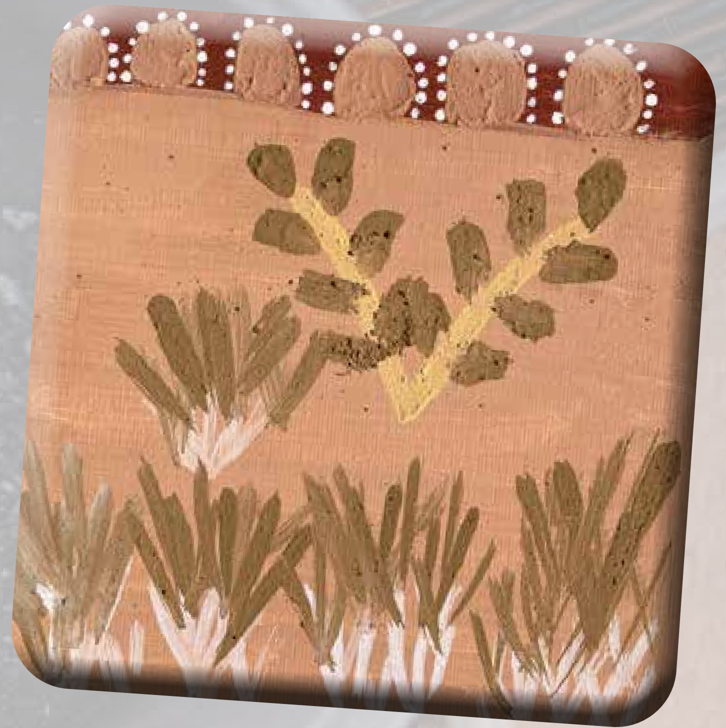

ROCHELLE PERIS

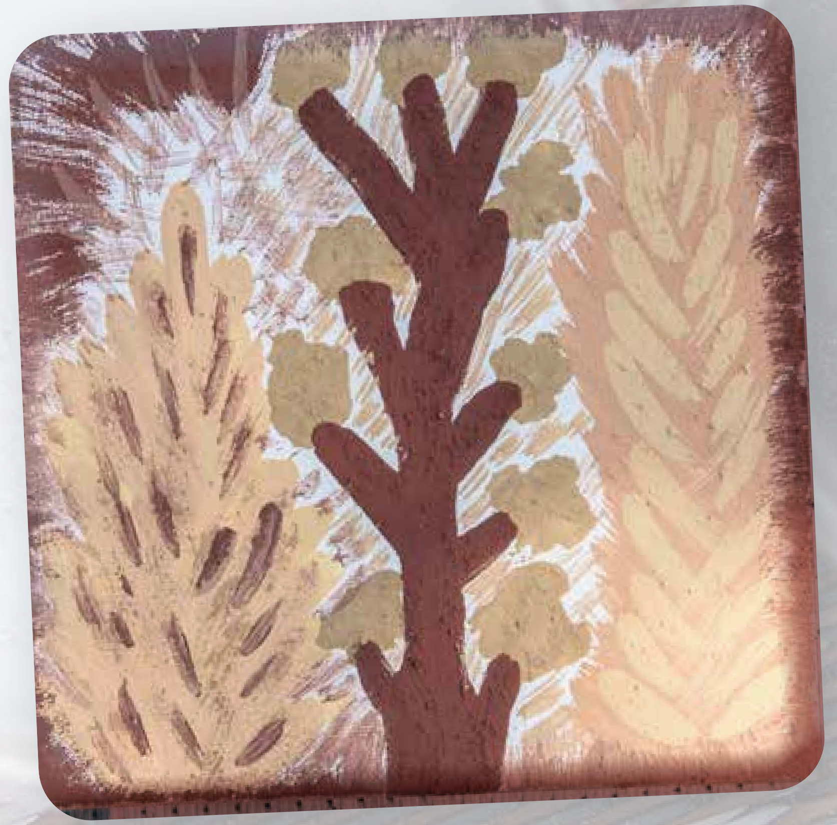

MADELINE PURDIE

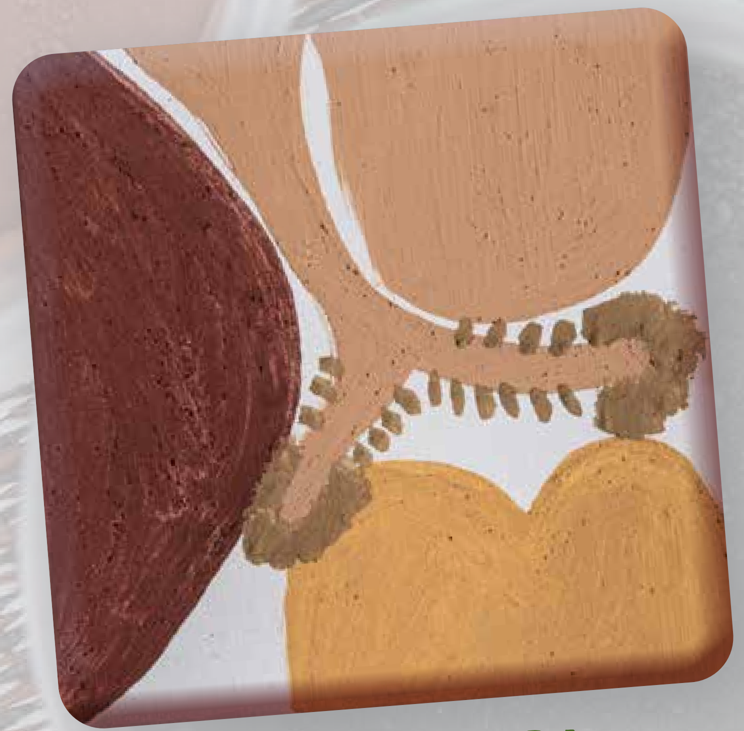

JANE YALUNGA

Background

The See, Treat, Prevent Skin Sores and Scabies (SToP) Trial is a shared project between researchers, service providers and communities to support skin health. In the early stages of the SToP Trial (2019 – 2021), the SToP Trial team sat with Warmun Elders and community members to understand the best way to promote healthy skin. The community conceived the idea of a healthy skin storybook featuring both traditional and Western approaches to supporting skin health.

In 2022, the SToP Trial team worked with Warmun community to co-design and co-develop this resource. The team was invited to attend the Two-Way Women's Healing camp, supported by the Warmun Local Drug Action Group, where stories and pictures of traditional bush medicines were shared and recorded. Students participated in skin health lessons organised by the health teacher, where they produced drawings of how they keep their skin strong. Finally, clinic staff members shared information about the clinic medicines commonly used to treat skin infections.

The language, art and traditional knowledge included in this resource is owned by Warmun community. Artists and translators were remunerated and gave permission for their work to be included in this resource.

The resulting resource is aligned with *Keeping Skin Healthy: A Handbook for Community Care Workers* (Pilbara, 2019) and *National Healthy Skin Guidelines* (2018).

The SToP Trial is a collaboration between Telethon Kids Institute, Kimberley Aboriginal Medical Services, WA Country Health Service - Kimberley and Nirrumbuk Environmental Health Services. The SToP Trial team sincerely thanks everyone who was involved in this project.

Ford A, Purdie M, Bray E, Koford F, Malgil R, Bray S, Sibosado S, McRae T, Gibbs T, Major A, Whelan A, Poore A, Thomas HMM, Bowen AC, *Dijan wen wi tokin bela propa good wan skin gota Gija (Gija Healthy Skin Story),* Telethon Kids Institute, Perth 2022

Copyright Telethon Kids Institute, 2022

Proudly supported by

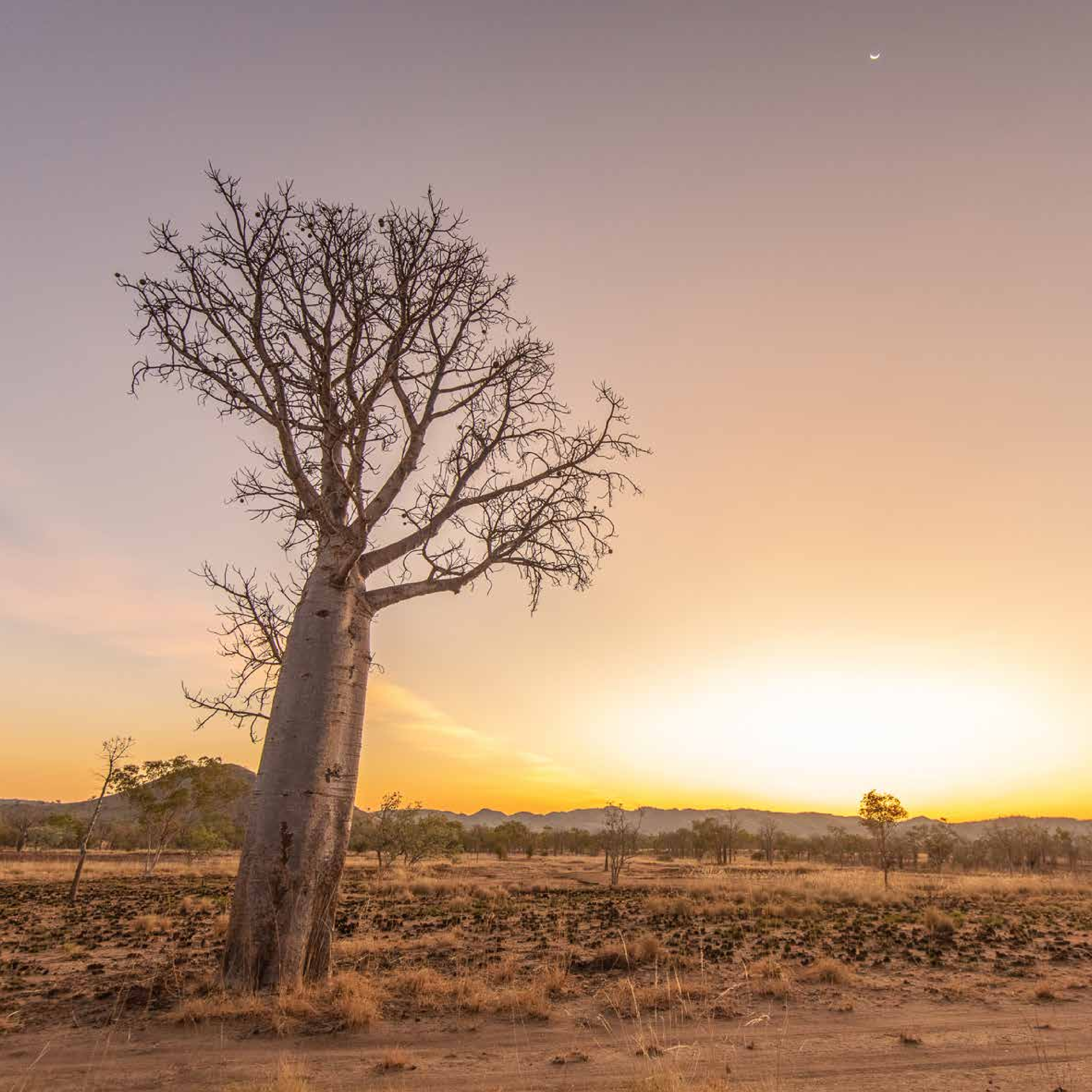

Supplement: Supplementary file 2 — Appendix 2: Dijan Wen Wi Tokin Bela Propa Good Wan Skin Gota Gija (Gija Healthy Skin Story). [file HPJA-36-0-s001.pdf]
